# ROC and confusion analysis of structure comparison methods identify the main causes of divergence from manual protein classification

**DOI:** 10.1186/1471-2105-7-206

**Published:** 2006-04-13

**Authors:** Vichetra Sam, Chin-Hsien Tai, Jean Garnier, Jean-Francois Gibrat, Byungkook Lee, Peter J Munson

**Affiliations:** 1 Mathematical and Statistical Computing Laboratory, DCB, CIT, NIH, DHHS, Bethesda, MD, USA; 2 Laboratory of Molecular Biology, CCR, NCI, NIH, DHHS, Bethesda, MD, USA; 3 Mathematique Informatique et Genome, INRA, Jouy-en-Josas, France

## Abstract

**Background:**

Current classification of protein folds are based, ultimately, on visual inspection of similarities. Previous attempts to use computerized structure comparison methods show only partial agreement with curated databases, but have failed to provide detailed statistical and structural analysis of the causes of these divergences.

**Results:**

We construct a map of similarities/dissimilarities among manually defined protein folds, using a score cutoff value determined by means of the Receiver Operating Characteristics curve. It identifies folds which appear to overlap or to be "confused" with each other by two distinct similarity measures. It also identifies folds which appear inhomogeneous in that they contain apparently dissimilar domains, as measured by both similarity measures. At a low (1%) false positive rate, 25 to 38% of domain pairs in the same SCOP folds do not appear similar. Our results suggest either that some of these folds are defined using criteria other than purely structural consideration or that the similarity measures used do not recognize some relevant aspects of structural similarity in certain cases. Specifically, variations of the "common core" of some folds are severe enough to defeat attempts to automatically detect structural similarity and/or to lead to false detection of similarity between domains in distinct folds. Structures in some folds vary greatly in size because they contain varying numbers of a repeating unit, while similarity scores are quite sensitive to size differences. Structures in different folds may contain similar substructures, which produce false positives. Finally, the common core within a structure may be too small relative to the entire structure, to be recognized as the basis of similarity to another.

**Conclusion:**

A detailed analysis of the entire available protein fold space by two automated similarity methods reveals the extent and the nature of the divergence between the automatically determined similarity/dissimilarity and the manual fold type classifications. Some of the observed divergences can probably be addressed with better structure comparison methods and better automatic, intelligent classification procedures. Others may be intrinsic to the problem, suggesting a continuous rather than discrete protein fold space.

## Background

A protein fold is often defined by the number, direction in space and connectivity (or topology) of its secondary structural elements[1] (alpha helices and beta strands). In two major fold databases, the definition of a fold is itself partially ambiguous. In SCOP[2], the definition is "same major number and direction of secondary structures with a same connectivity", without quantification of the term "major". In CATH[3], it is "overall shape and connectivity of the secondary structures", without a precise definition of "shape", although there is a degree of quantitation in this case since a structure comparison score is used to cluster domains in the same fold family. These "soft" definitions are required by the observed variations in the structures between proteins of identical biochemical function as amino acid sequence identities fall below 40%[4].

The situation is complicated by the presence of domains in protein structures. Their identification and delineation are not straightforward. Nevertheless, to have a better understanding of the effect of discrete classification as a description of the fold space, we analyzed the SCOP domain classification using two structure comparison methods applied directly to these domains. Numerous structure comparison methods exist [5-19] and some of them have been used to conduct such analyses. Shapiro & Brutlag[14], Ye & Godzik[17] and Kolodni et al[20] used the Receiver Operating Characteristic (ROC) curve[21,22] and Sierk & Pearson[23] a variant of it, mainly to compare their own method with other methods, using SCOP or CATH as the gold standard. Getz et al[24] devised an optimization algorithm to automatically classify new domains into existing SCOP folds or CATH topologies. They did not use the ROC curve, and only present the pairwise similarity score matrix. They also noted the existence of folds which are in twilight zone and difficult to classify. Hadley & Jones[25] and Day et al[26] compared 3 classifications: SCOP, CATH and FSSP ranging from completely manual to entirely automatic. They give the coverage, i.e. the percentage of pairs that are common between the 3 classifications, the percentages of pairs that are common to all 3 methods. Hadley and Jones[25] in their analysis briefly described a few examples of structural discrepancies between the automatic method FSSP and the manual and semi-manual SCOP and CATH classifications.

Here we use two structure comparison methods which are based on different principles and with which we are familiar. One, VAST[5,6], is based on only secondary structure elements in its first stage of comparison while the other, SHEBA[16], uses the amino acid sequence along with other structural properties of each residue in its initial step. We first construct the ROC curves using the SCOP fold definitions. We then generate the confusion matrix that results after setting a score cutoff value determined from the ROC curves. We analyze various aspects of this matrix to understand and extract the main properties of the fold space which cause the divergence with the automatic similarity assignment and the manual SCOP classification. Although some of the previous works[14,17,20,23-28] cover portions of what we describe here, none, as exhaustively analyzes and lists the fundamental mechanisms that produce the observed divergences.

## Results

### ROC curves

**Figure 1:**
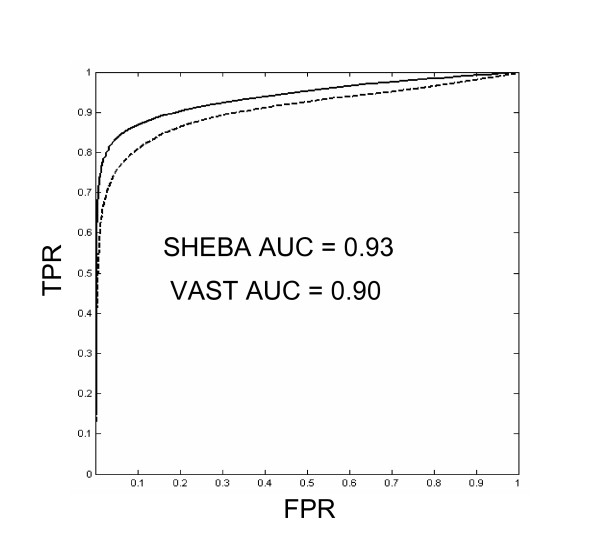
**ROC Curves**. ROC curves of VAST (dotted line) and SHEBA (solid line) obtained by plotting the True Positive Rate (*TPR*, eq. 1, see Methods) against the False Positive Rate (*FPR*, eq. 2, see Methods). Area Under the Roc Curve (AUC) for VAST is 0.90, AUC for SHEBA is 0.93.

The ROC curves of each method show that both VAST and SHEBA are generally successful in detecting when two domains are in the same SCOP fold (Figure 1). The ROC AUC (see Methods) is 0.93 for SHEBA, and 0.90 for VAST, indicating that SHEBA recognizes SCOP folds slightly better than does VAST. Also, the SHEBA ROC curve is above the VAST ROC curve at every point; there are no points of crossing, indicating that SHEBA is uniformly better than VAST at this recognition task. The ROC curve we present is actually an average of the curves obtained for each individual SCOP fold-recognition problem using a common cutoff value for all problems. For certain individual problems, VAST may dominate SHEBA or vice versa.

An optimal cutoff value for the binary decision of similarity can be determined from the ROC curve either by specifying the desired *FPR *(False Positive Rate, see Methods) or by specifying the desired *TPR *(True Positive Rate, see Methods). To reach a 1% *FPR*, the corresponding cutoff value is 2.5 for *Pcli *and 2.7 for *Zscore *(see Methods), with corresponding *TPR *values of 61.6% and 74.8% for VAST and SHEBA, respectively.

### Confusion matrix heat maps

**Figure 2:**
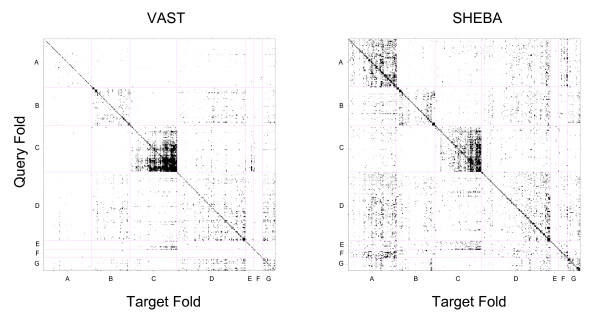
**Confusion matrix heat map**. Confusion matrix heat map for VAST with a *Pcli *cutoff value of 2.5 and for SHEBA with a *Zscore *cutoff value of 2.7. The cutoffs correspond to an overall average *FPR *of 0.01, and result in an overall average *TPR *of 0.616 and 0.748 for VAST and SHEBA respectively. The x (target folds) and y (query folds) axes of the heat maps are labeled by the SCOP folds, grouped into classes A, B, C, D, E, F and G. Each class is delimited by a vertical line (for the x axis) and a horizontal line (for the y axis). Each pixel within the heat maps represents a fold-specific true or false positive rate and takes value between 0 and 1. Diagonal and off-diagonal pixels correspond to fold-specific true positive rate *TPR*_*i*_(c) (eq. 4, see Methods) and fold-specific false positive rate *FPR*_*i*,*j*_(c) (eq. 3, see methods) respectively. To improve the visibility of the heat maps, rates between 0 and 0.2 are represented in grey scale where white corresponds to a rate of 0 and black to a rate at or above 0.2. For high resolution heat maps of VAST and SHEBA, [See Additional file 1].

Figure 2 shows the confusion matrix heat maps. An entry (*i*, *j*) of each matrix indicates the fraction of pairs of domains, one from each folds *i *and *j*, that are judged to be similar by the automatic similarity detection method using the cutoff value that produces 1% *FPR*. These maps constitute the basis for the analysis of the properties of the methods and the fold definition, conducted below. For high resolution heat maps of VAST and SHEBA, [See Additional file 1].

Neither the VAST nor the SHEBA heat map is strictly symmetric (Figure 2); the computed similarity measure depends on which domain is used as query and which as target. SHEBA gives a substantially more asymmetric heat map than VAST. Out of the total of M*(M-1) = 21,860,300 domain pairs (excluding identity pairs) on which the heat map is based, VAST and SHEBA have 11,007 and 189,551 asymmetric pairs, respectively. A domain pair similarity score is considered asymmetric if its similarity score exceeds the cutoff value in one comparison, but does not when the query and target structures are exchanged.

VAST uses an heuristic algorithm to find the maximal clique so the comparison of domain A with B may not select the same clique as the comparison of B with A when there are several near maximal cliques. The result is a slight asymmetry in the *Pcli *Score. The more noticeable asymmetry manifest by SHEBA is due to the *Zscore *computation which uses the average and the standard deviation of the distribution of *m*-scores between a fixed query domain and all other domains in the database, making the *m-score *distribution dependent on which domain, A or B, is declared the query domain. Since the average *m-score *similarity of a query domain A to the database may depend on parts of A which are not matched to B, the average similarity of B to the database might be quite different. Hence the *Zscore *becomes asymmetric.

### False negatives

**Figure 3:**
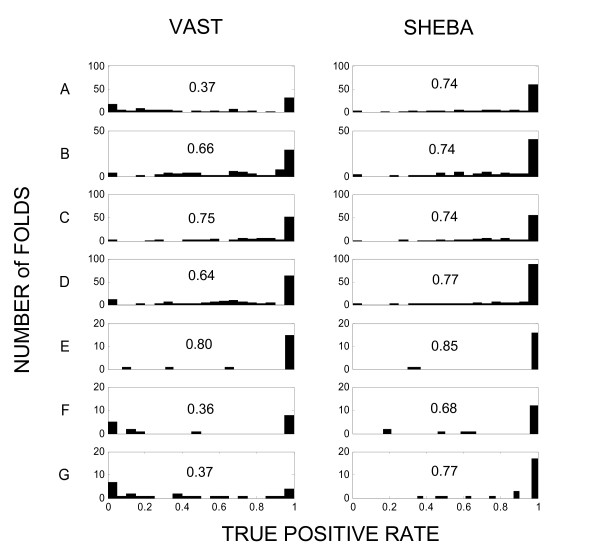
**Distribution of true positive rates**. Distribution of fold specific true positive rates within each SCOP class (A to G) for VAST and SHEBA. *TPR*_*i *_(eq. 4, see Methods) are obtained using same cutoff values as in Figure 2. The scale of the y axes for VAST and SHEBA distributions are the same within fold class. Histogram bar height represents the number of folds for a given range of *TPR*_*i*_. The x axis is divided in 20 bins. The class-specific average *TPR*_*i *_is reported within each subplot. For the list of *TPR*_*i *_obtained by each fold, with VAST and SHEBA, [See Additional file 2].

The true positive rate varies with fold class, as illustrated in Figure 3. SCOP similarity detection differs widely among folds within a class and between the two methods. We now seek explanations of this variation.

About 40% of the folds (216) achieve a fold specific true positive rate (*TPR*_*i*_) above 0.85 for both methods. All classes are nearly proportionally represented in this set. For the exhaustive list of *TPR*_*i *_obtained by each SCOP fold with VAST and SHEBA, [See Additional file 2].

To investigate why some domain pairs in the same SCOP fold are not detected as similar, we look at such domain pairs that belong to the same SCOP fold and for which the *Pcli *and *Zscore *values are below 1 and 1.6, respectively. These low cutoff values correspond to a *FPR *of 5%, and are chosen to exclude from consideration any borderline cases with computed similarity near but just below the original cutoffs. For all classes A to G, a total of 144 folds contain such extreme false negative domain pairs with 36 in A, 30 in B, 30 in C, 36 in D, 2 in E, 5 in F and 5 in G. The complete list of these folds is provided in Table 1.

Detailed analysis of these false negative pairs highlights some common factors which explain the varying success of automated methods in detecting the similarity among domains in a SCOP fold. Most of the false negatives can be explained by structural variation within a fold and to a lesser extend by structures made of repeating units.

#### Structural variation of the common core

**Figure 4:**
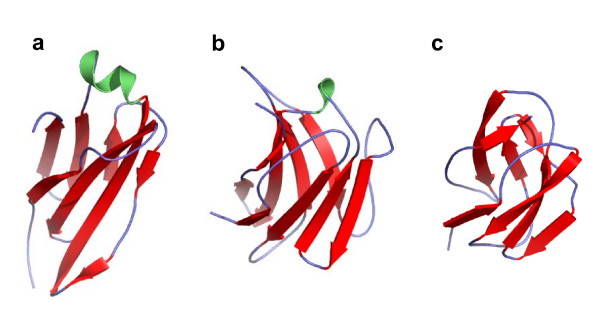
**Structural variations within fold b.1**. Domains (a) d1c5ch2, (b) d1akjd_, and (c) d1pama1 belong to the fold b.1 (Immunoglobulin-like beta-sandwich; 7 strands in 2 sheets greek-key, some members of the fold have additional strands). Domain pair (a) and (b) have *Pcli *= 4.7 and *Zscore *= 3.09; domain pair (b) and (c) have *Pcli *= 4.2 and *Zscore *= 3.34; and domain pair (a) and (c) have *Pcli*=-0.5 and *Zscore *= 0.11. Domains d1c5ch2, d1akjd_, and d1pama1 have 103, 114, and 86 residues, respectively. The helices are colored in green, the strands in red, and the other regions in blue. This and all other structure figures were prepared using Pymol [43].

In many cases, the structure of the *common core*[29] of a fold varies significantly from one domain to the next in the same SCOP fold. We observe this phenomenon in folds across all SCOP classes. Many of the extreme false negative domain pairs described above are examples of such cases. Figure 4 shows the three domains, d1c5ch2 (a), d1akjd_ (b), and d1pama1 (c), from fold b.1. The similarity for pairs (a, b) and (b, c) is detected by both VAST and SHEBA, while it is not for the pair (a, c). The relative orientations between the beta sheets which form the beta sandwich, in domains (a) and (c) vary from those in domain (b). This variation is important enough, with regard to thresholds admissible by VAST and SHEBA, to make superposition of the structures (a) and (c) difficult, and to prevent a similarity detection. This results in a loss of transitivity for automatic similarity detection.

#### Structures made of repeating units

**Figure 5:**
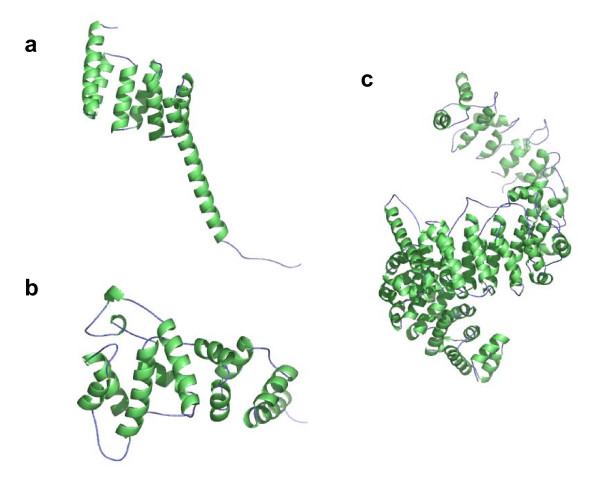
**Repeat of a structural motif within fold a.118**. The color scheme is the same as in Figure 4. Structures of domains (a) d1a17_, (b) d1kula_ and (c) d1qbkb_ from fold a.118 (alpha-alpha superhelix, multihelical; 2 (curved) layers: alpha/alpha; right-handed superhelix). Domains have 159 residues and 7 helices, 211 residues and 10 helices, and 888 residues and 48 helices, respectively. The VAST similarity score *Pcli *assigned to the domain pair (a) and (b) is -2.3, to (a) and (c) is -3, and to (b) and (c) is -8. The SHEBA *Zscores *are respectively 1.8, 1.3, and 1.3. The negative values reported for the *Pcli *should be interpreted as values very close or equal to zero (no similarity), and resulted from the use of an approximation for the computation of *Pcli*.

Automated similarity detection methods do not necessarily consider two structures similar if they contain the same simple structural motif but with a different number of repeats. The SCOP fold a.118 provides an extreme example. It is defined by domains that are comprised of repeated occurrences of a helix-loop-helix motif[30]. The number of occurrences of the helix-loop-helix motif varies greatly and is unspecified by the fold definition. Figure 5 shows three members in this fold and gives their pairwise similarity scores assigned by SHEBA and VAST. The d1qbkq_ (c) domain contains many repeats and is much larger (888 residues) than d1a17_ (a) and d1ku1a_ (b) domains (159 and 211 residues, respectively). Since both VAST and SHEBA look for global similarity, and since d1a17_ or d1kula_ would match at best only a small part of d1qbkq_, they yield the low Pcli and Zscore values. Size difference does not account for the low score between domains d1a17_ and d1ku1a_. Here, the reasons are that the helices of the repeated motifs vary in length and that the relative orientation of each motif varies between the structures. Thus, a multiple occurrence of locally similar motifs between two domains does not always produce a high global similarity score.

#### Decoration of the common core by many secondary structure elements

**Figure 6:**
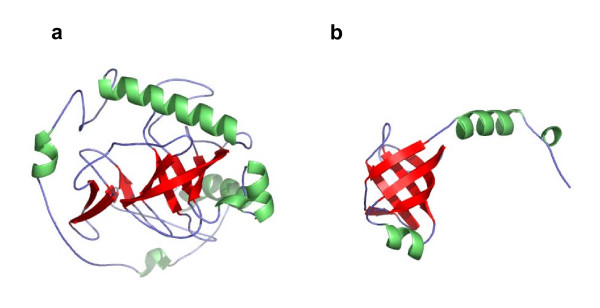
**Decoration of a common core**. Structures of domains d1e9ga_ (a) and d1enfa1 (b) of SCOP fold b.40 (barrel, closed or partly opened n = 5, S = 10 or S = 8; greek-key). Color scheme is the same as in Figure 4. Domain (a) has 284 residues, and (b) has 100 residues. *Pcli *and *Zscore *values assigned by VAST and SHEBA to this pair are 0.1 and -1.3, respectively.

Occasionally two proteins in the same SCOP fold share a common core but are different in overall shape. An extreme example is shown in Figure 6 for the domain pair d1e9ga_ and d1enfa1 in the fold b.40. They both contain a beta barrel, but the beta barrel in domain d1e9ga_ is only a small part of its entire structure. A match between this domain and domain d1enfa1, based on the conserved common core is thus not found sufficient to consider them to be similar by the automatic pair-wise structure comparison methods.

#### Miscellaneous cases

Some folds, such as fold d.184 or a.138, are described in SCOP as including a variety of structures. We also note the existence of several ambiguous fold definitions leading necessarily to a low *TPR*_*i*_. For instance, fold c.37 whose SCOP description is "3 layers: alpha/beta/alpha, parallel or mixed beta-sheets of variable sizes", can probably be split into at least 2 folds. We also spotted what appears to be a bookkeeping error by SCOP. Domains d1kkea2 and d1qiua2 of fold b.83 were not found to be similar either by VAST or SHEBA. The protein 1kke has two domains, which belong to two different folds. The N-terminal domain (residues 250–312) forms an extended structure belonging to the SCOP fold b.83 ("Triple beta-spiral"). The C-terminal domain (residues 313–455) forms a beta barrel belonging to the SCOP fold b.21 ("Virus attachment protein globular domain"). In SCOP and in the Astral database, the domain d1kkea1, which consists of the residues 250–312, is placed in the b.21 fold and d1kkea2, which consists of residues 313–455, is placed in the b.83 fold.

#### Differences between VAST and SHEBA

There are 27 folds with a *TPR*_*i *_below 0.05 by VAST yet above 0.9 by SHEBA. They are a.16, a.37, a.38, a.97, a.115, a.121, a.130, a.158, a.159, b.76, c.107, d.6, d.83, d.88, d.101, d.118, d.175, f.10, f.14, f.17, g.14, g.22, g.24, g.38, g.49, g.50, g.53. These are mainly small folds with only 2 domains each. No fold has been identified with a *TPR*_*i *_less than 0.05 by SHEBA but above 0.9 by VAST. Additionally, the class specific true positive rates reported in Figure 3, shows an important difference between VAST and SHEBA in the A class (0.37 for VAST and 0.74 for SHEBA).

Some of the differences observed between VAST and SHEBA are related to the calculation of the scoring function in VAST (see Appendix, Calculation of *Pcli*), and to the fact that structures sharing fewer than 3 secondary structure elements (SSEs) are often judged not significant by VAST. This latter factor also affects true positive rates computed by VAST in the A class, where the *TPR*_*i *_averages only 0.2 for folds with 2,3 or 4 SSEs, but rises to 0.7 when the fold has about 9 or more SSEs (data not shown). But at least one case could not be explained by the issue of the *Pcli *calculation. Domains d1h8pa1, d1l6ja3, d1pmla_, d2hpqp_and d2pf1_1, of fold g.14 defined as a disulphide-rich fold, scored low in VAST similarity but surprisingly high by SHEBA. These domains have particularly small SSEs, distributed sparsely over the backbone of the structure. It is quite understandable that VAST which relies on the SSEs, finds low similarity among them. It was also observed that pairs for which SHEBA *Zscore *was high also had a higher level of sequence homology than those for which the SHEBA *Zscore *was low (data not shown). This indicates that SHEBA benefited by using the sequence homology in finding the initial alignment (see the Methods).

### False positives

The off-diagonal pixels in the heat maps, on Figure 2, represent fold pairs having a non-zero fold-specific false positive rate *FPR*_*i*,*j*_. The confusion made by each method has different characteristics, shown by the difference in the distribution of the dark areas. There are a relatively small number of pixels between classes. In contrast, confusion within each class varies with the method and can be high.

The main confusion is within classes B, C and D, with respectively 37 folds out of 78 within B class, 80 folds out of 94 within class C, and 53 folds out of 139 within class D, involved in some type of confusion. VAST does not show a noticeable level of confusion within classes A, and F, although SHEBA does. The relatively high A-class confusion level for SHEBA is probably related to its use of the dynamic programming algorithm, without gap penalty, in finding the best alignment between a pair of superimposed structures[16].

Besides these global observations, more specific confusion trends can be determined by analyzing the predominant confusion patterns shown by the heat maps.

#### Intraclass confusion

Confused folds occur mainly near the diagonal of the sorted heat map, as a result of the hierarchal clustering and re-ordering of the folds within each fold class (see Methods).

Table 2 reports a number of clusters of confused folds within classes A, B, C and D common to VAST and SHEBA.

Confused folds in the A class include helix bundles of either identical or a similar number of helices in similar relative orientations. Examples are reported in Table 2, rows 1, 2 and 3. The close similarity of some domain pairs, for example d1m7ka_ and d1hs7a_ belonging to folds a.7 and a.47, respectively, indicates that these "confusions" appear to be cases wherein SCOP includes considerations other than purely structural similarity/dissimilarity.

**Figure 7:**
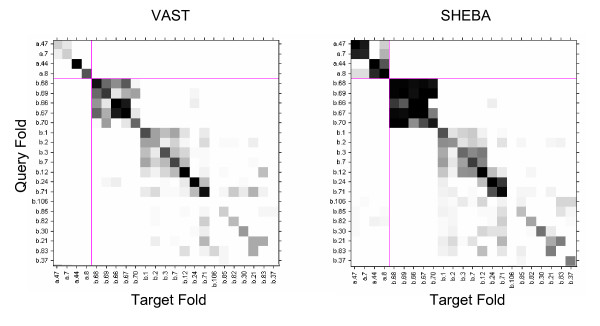
**Confusion matrix for the B class**. Confusion matrix heat map for VAST and SHEBA showing confusion among some SCOP folds of the class B, mainly beta domains. Fold identifiers appear on the x and y axis. Grey scale from white to black for positive rates from 0 to 1.

**Figure 8:**
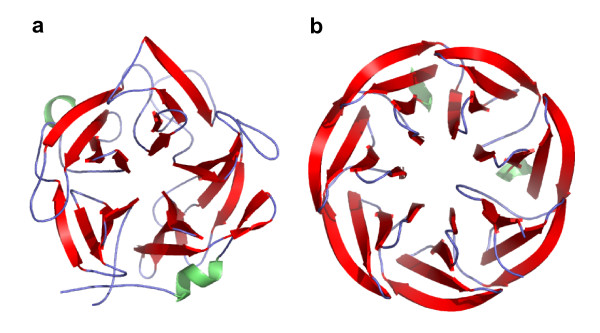
**Similar structures in different SCOP folds**. Structures of domains (a) d1gyha_ of fold b.67 and (b) d1loqa2 of fold b.69, with 318 residues and 295 residues respectively. They correspond to beta propeller domains with respectively 5 and 7 four-stranded blades. The *Pcli *and *Zscore *values are 5.2 and 7.2, respectively. Color scheme is the same as in Figure 4.

Figure 7 illustrates clusters of confused folds within class B, which are found in both the VAST and SHEBA heat maps. There are two large clusters of confused folds in Figure 7. The darkest area covers the five beta-propeller folds (Table 2, row 4), with each fold containing different number of blades ranging from 4 to 8. These tend to be highly confused with each other, more by SHEBA than by VAST. Figure 8 shows domains d1gyha_ (a) and d1loqa2 (b) from folds b.67 and b.69 respectively. Since the 7 bladed beta-propeller domain can have up to 5 blades common with the 5 bladed beta-propeller domain, pairs of domains from these separate folds tend to have a high similarity scores.

The next cluster of five folds in Figure 7 (Table 2, row 5), includes all beta sandwich immunoglobulin-like folds, with 7, 8 or 9 strands in 2 sheets with a Greek-key topology. Their confusion is caused by the sharing of the motif of the beta sandwich of the common core. Others confused sets of folds in the B class also involve mainly beta sandwich folds (Table 2, rows 6, 9, and 12), or beta barrel folds (Table 2, rows 7, 8, 10 and 11). The confusion among domains of these clusters of folds is similarly caused by a common beta sandwich or beta barrel motif. In the B class, where folds defined by the beta barrel or the beta sandwich motifs are frequent, confusion among folds of either motif is frequent as well.

**Figure 9:**
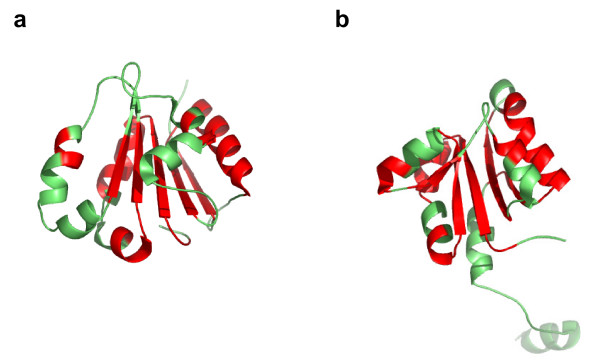
**Superposition of two structures**. Superposition by VAST of two structures from different 3 layers alpha/beta/alpha SCOP folds of class C. View of backbones of domains (a) d1a8p_2 and (b) d1a9xa2, from folds c.25 and c.24, respectively. The common parts of both structures superposed by VAST, are in red and the unmatched residues in green. The superposition aligned 71 residues; d1a8p_2 has 158 residues and d1a9xa2 has 138; RMSD = 2.7, *Pcli *= 6.0. SHEBA *Zscore *is 3.4. The SCOP definition of fold c.25 is: Methylglyoxal synthase-like; *3 layers, alpha/beta/alpha; parallel beta-sheet of 5 strands, order 32145*. The SCOP definition of fold c.24 is: Ferredoxin reductase-like, C-terminal NADP-linked domain; *3 layers, alpha/beta/alpha; parallel beta-sheet of 5 strands, order 32145*.

A large common confusion pattern among folds appears at the bottom right corner of the C class area of the heat map (Figure 2). A highly confused set, Table 2 row 17, from this large confused area consists of 3 layer alpha/beta/alpha folds with parallel beta sheets for some, and mixed beta sheets for other, and with 4, 5, 6 or 7 strands. The superposition of the domains d1a8p_2 (a) and d1a9xa2 (b), from folds c.25 and c.24 respectively (Figure 9), illustrates that confusion is caused by the presence of a common sub-structure. Examination of other confused folds from this large confused set within the C class in Table 2, rows 15, and 16, shows that they also involve 3-layer alpha/beta/alpha folds that share sub-structures of varying sizes that are similar. The large number of confusions in the C class can be attributed to the abundance of the 3-layer alpha/beta/alpha folding pattern, which get confused by a similar mechanism.

The C class also shows some small confused sets among folds with different architectures. For example, confused folds c.1 and c.6 (Table 2, row 13) correspond respectively to TIM beta-alpha barrel and variants having 7 strands or less. Confused folds c.8 and c.98 (Table 2, row 14) are described respectively as "3 layers: beta/beta/alpha; the central sheet is parallel, and the other one is anti-parallel" and "core: 3 layers, alpha/beta/alpha; parallel beta-sheet of 4 strands". The confusion occurs due to a common beta/alpha sub-structure.

**Figure 10:**
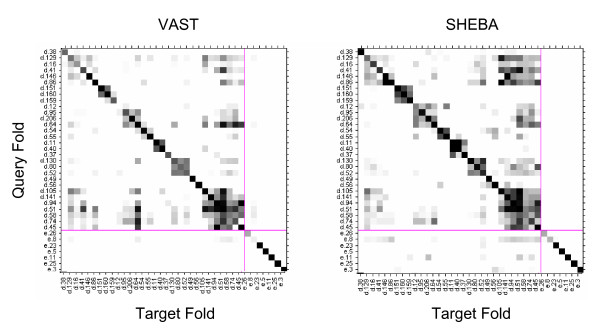
**Confusion matrix heat map for the D class**. Confusion matrix heat map for the D class for VAST and SHEBA showing clusters of confused SCOP folds. The fold identifiers appear on the x and y axis. Grey scale from white to black for positive rates from 0 to 1.

Figure 10 shows several clusters of varying size, common to VAST and SHEBA within the D class. They correspond to the sets of confused folds reported in Table 2, row 23, 24, 25, 26, 27. Folds in these sets share a common core structure consisting of a 2-layer alpha/beta sandwich. An analysis of the clusters reported in Table 2 for the D class shows that most of the confused folds are mainly variations of the 2-layer alpha/beta sandwich structures.

**Figure 11:**
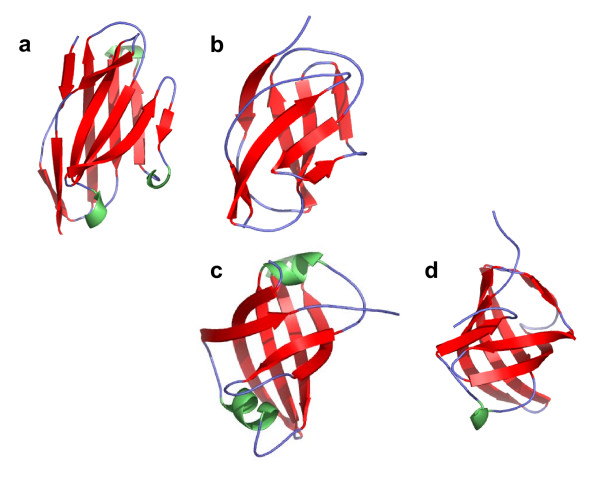
**Confusion between SCOP folds of class B**. Color scheme is the same as in Figure 4. Domain (a) d1tvda_ and domain (b) d1pama1 belong to the same fold, b.1 (sandwich; 7 strands in 2 sheets; greek-key), and are found similar with *Pcli *= 3 and *Zscor*e = 3.38. Domain (c) d1ep3b1 and domain (d) d1d2ea1 belong to the same fold, b.43 (barrel, closed; n = 6, S = 10; greek-key), and are found similar with *Pcli *= 4.5 and *Zscore *= 3.7. Domains (b) and (c) belong to folds defined by different folding patterns. Both VAST and SHEBA found them similar with *Pcli *= 3.1 and *Zscore *= 3.32. Domains (a) and (d) were found dissimilar by VAST and SHEBA with a *Pcli *= -1.8, and a *Zscore *= 0.73.

We have noticed confusions involving distinct motifs such as between the beta sandwich fold b.4 and beta barrel fold b.107, (Table 2, row 9). Beta sandwich and beta barrel motif folds are generally well separated, but false positives due to proximity of some extreme members of these respective folds can happen. Figure 11 reports a false positive between domains from the beta sandwich fold b.1 and beta barrel folds b.43, which are well distinguished on average. Domain d1pama1 (b) from fold b.1 is confused with domain d1ep3b1 (c) from fold b.43. Such confusion is caused by structural variation of the common core of the beta sandwich fold b.1 and barrel fold b.43 represented by prototypical domains d1tvda_ (a) and d1d2ea1 (d), respectively. Deformation causes the relative orientation between the beta sheets of domain (b) to become more similar to that of the barrel domain (c).

#### Interclass confusion

Finally, the heat maps also show off-diagonal grey or black pixels where members of a SCOP fold in one class are detected as similar to domains in another. Both heat maps present such confusion patterns. As apparent in Figure 2, the confusion between classes is very low for both methods. Nevertheless, it is still detectable between some classes, in particular, between classes B and D, C and D, C and E, and between D and G. For VAST, there is no additional noticeable confusion between classes. However SHEBA shows additional minor confusion of the class A with the classes B, C, D, F, and G, and between classes D and F.

The confusions involving the E-class ("Folds consisting of two or more domains belonging to different classes") are easily understandable. They all involve structures which contain a domain which shares similarity with another domain in a different class, mainly class C. Examples include fold e.24 confused with c.16, c.57, c.44, c.23 and c.5, (Table 2, row 28), and fold e.4 confused with folds c.48, c.2, c.32, c.33, c.34 and c.23, (Table 2, row 29).

Additionally, SHEBA confuses some folds from class A, with folds in classes D and F ("membrane proteins"). The most confused folds from the A and D classes, having more than 100 confused domain pairs, are: (a.118, d.211: 250 confused pairs), (a.60, d.58: 132), (a.1, d.58: 118), (a.77, d.58: 114), (a.6, d.58: 104), (a.4, d.95: 104). For confused folds a.118 and d.211, for example, even though VAST and SHEBA match a similar number of residues, the Sheba *Zscore *tends to be high while the VAST *Pcli *is below the cutoff value. A similar trend is observed between the A and F classes. The way the *Zscore *is computed has the tendency to increase the confusion, by over-emphasizing the significance of the match, compared to the number of matched residues when helices are matched.

## Discussion

The combined use of the ROC curve and the confusion matrix heat map has been the key in making this large scale analysis of protein classification. Several authors[14,17,20,23] have used the ROC curve to evaluate structure comparison methods using the CATH or SCOP protein classification database as the reference. In the most recent and comprehensive study, Kolodny *et al*[20] compared six different methods and found the highest true positive rate to be 50%, at 1% false positive rate, attained by the DALI method using the native DALI score and CATH as the reference. Our ROC analysis finds a true positive rate of 61.6% and 74.8% at 1% false positive rate, for the comparisons of VAST and SHEBA to SCOP, respectively. The differences between their result and ours might be explained by differences between the comparison methods (DALI, VAST, SHEBA), by differences between the definitions used for the false and true positive rates (they do not give explicit equations), and/or by the use of different databases of protein structures (CATH vs. SCOP). In particular, CATH groups domains into different numbers of folds than does SCOP, as noted by Hadley & Jones[25] and Day *et al*[26].

Aside from providing a global measure of the agreement, ROC curves are also useful because they provide a practical means to select a score cutoff value for deciding if a pair of structures is to be considered similar or not, by trading off true and false positive rates. Other approaches have used methods other than ROC analysis or have ignored that tradeoff entirely. In their comparison of several structure comparison methods with CATH, Sierk and Pearson[23] selected a decision level corresponding to the first 100 errors made by the program. Other approaches [24-28] do not use the ROC curve and often fail to properly acknowledge the obligatory trade off between false and true positive rates, making it difficult to compare the reported degree of agreement with others.

Although the ROC AUC varies somewhat by method, none of the reported values are high as desired. This raises a fundamental and important question: What mechanisms cause the automatic structural comparison methods to diverge so significantly from SCOP or CATH? To address this aspect of the problem, we need to descend from a global view of the database to a more detailed view of individual folds and finally of the domains comprising each fold. To investigate why structural comparison methods diverge from SCOP, we used the confusion matrix to distribute the 1% false positive comparisons to the individual fold pairs, resulting in a "false and true positive rates" map of the protein fold space. This can be distinguished from the map of the fold space constructed by Hou *et al.*[31,32] who applied multi-dimensional scaling to pair-wise similarity scores. The exploration of the fold space, guided by our map, leads directly and objectively to the areas or subsets of folds where divergence with structural comparison methods is most evident. In particular, it has allowed us to move from the areas of high false positive or negative rates to the corresponding properties of the fold space. False negative rates are seen to relate directly to the issues of core variation and repeated sub-structures within a fold, while false positive rates are linked to the sharing of a common sub-structure between folds. Since the mathematical quantities *FPR *and *TPR *are interdependent, so are the corresponding properties of the folds space.

In looking at a particular area of our heat map, we can calculate an index of how likely a method is to confuse those folds, as the ratio of the average of fold-specific false positive rates to the average fold-specific true positive rate in that area. A value near 1 indicates that the folds in this area cannot be distinguished by the structure comparison method, on the average. It is worth noting that this index is cutoff dependent, as expressed in terms of true and false positive rates, and can thus be obtained for more or less severe false positive rates. The index of confusion is related but distinct from the index of "gregariousness" in Harrison *et al.*[13] for the CATH folds (topology level), which is a property of a fold that measures the number of other folds that are similar to it as judged by comparing the score to that of an empirically established standard score distribution at a certain cutoff level. The substantial number of highly confused sets of folds listed in Table 2 allows us to examine in detail the source of the discrepancy between SCOP and our structure comparison methods.

### Causes of false negatives and false positives

In the Results section we presented several examples of false negative and false positive cases related in one way or another to the common core. SCOP defines the common core of domains in the same fold to have the "same secondary structure elements in the same arrangement with the same topological connections" (Brenner et al[29]), leaving open the possibility for some variation such as differences in length, relative orientations and/or number of the SSEs which we call variation of the common core.

Variation of the common core of domains within a fold, considered insignificant by SCOP, may still be large enough to cause VAST and SHEBA to find the domains dissimilar, giving rise to false negatives as in Figures 4 and 5. False negatives may also occur when the common core is so small compared to the whole structure that the overall structural similarity is unrecognizable, as in Figure 6. The evidence of structural variations of the common core of proteins within the same fold was shown in the work by Chothia & Lesk[4]. When the percentage of sequence identity between domains decreases much below 40%, their common cores tend to diverge structurally. The analysis of the confusion matrix shows that some false negatives for folds reported in Table 1 arise from such core structure variations.

When two domains share an apparent common core, but SCOP judges the core elements to be significantly different, SCOP places the domains in distinct folds. However, the automatic methods may find the domains similar, as in Figure 9 and 11, giving rise to false positives. Also, conversely to the case in Figure 5, when the repeats of a common motif are organized in a regular fashion in a domain, our methods may consider the domains similar, but SCOP may place them in distinct folds (see Figure 8). Table 2 enumerates a number of false positive cases arising from closely related common cores in distinct SCOP folds.

VAST and SHEBA decide on the similarity on the basis of the largest fraction of matching secondary structural elements or residues. However, visual inspection may allow the overall context of the matching and mismatching parts to play a role. If only a small part matches, but the matching part appears to be the core of each structure, then the match may appear more meaningful. If the number of repeats in a structure appears to be an important property of the structure, structures with different numbers of repeats may be placed in different folds. If, on the other hand, the precise number of repeats is not important for a structure, structures with different numbers of repeats are all placed in the same fold. If almost all parts match, but some important part, perhaps one critical beta-strand or even an irregular loop, is missing or placed differently in one structure, it may be placed in a different fold, etc.

It is possible that the problem is rooted in part, in the way structural alignment is currently conceived. Analogous to sequence alignment methodology, structural alignment maximizes the match between two structures, at the residue or secondary structure level, to infer a similarity relationship. On the other hand, the concept of similarity implicitly defined by SCOP, is focused on the sharing of higher level (above SSEs) motifs. This is in contrast to similarity measures based on the residue or SSE-level matches as defined by many structure comparison methods. We have shown examples (beta propellers, or alpha-solenoids) where occurrence of a motif is more appropriate for inferring similarity than is the maximum residue or SSE-level structural match. Although not evaluated directly here, we suspect that the structural comparison methods agree with SCOP when these two concepts agree, i.e. when the motif in question coincides with the maximum residue or SSE-level structural match, but disagree otherwise. Automatic structural similarity measures might thus be improved either by incorporating higher level structural motifs such as barrels or sheets, rather than remaining at the level of residues, strands or helices, or by weighting matching residues according to their structural context or functional importance.

Problems encountered by structural comparison methods might also be a reflection of intrinsic properties of the protein fold space. We have reported examples which tend to support the idea of structural drift [33], i.e. a series of gradual steps which connect one fold with another, and showed areas where folds were highly confused. In such sets of folds, some structures within the same fold are too dissimilar to be detectable by structural comparison methods, while those in different folds are not always completely distinct. This raises questions about the fold definition. We have observed, for example, that distinction between beta barrel and two layer beta-sandwich domains can be surprisingly difficult. As the relative orientations of the strands in the two beta sheets in a barrel departs from orthogonality, and become more parallel, the distinction between barrel and two layer beta sandwich motifs becomes fuzzy. Drawing the proper separations within a set of domains in which such phenomenon is observed is not obvious and necessarily introduces some arbitrariness. Should such diverse folds be sub-divided into two, three or more folds? If this decision is taken at some point in time, with the then available structures, how stable and universal will this distinction remain over time? VAST and SHEBA are generally well able to a major part reproduce the fold classification of SCOP, consistent with the notion that protein folds are well-defined, discrete entities. However, despite many attempts, SCOP folds or CATH topologies continue to elude precise quantitative or computational definition. We suggest therefore, that for some parts of the fold space, folds are not well separated entities but more nearly a continuum of structural arrangements as also observed in [1,3,34-36], with some regions more populous than others. Here, apparent "folds" may arise as much from density fluctuations in regions where experimentally determined structures are sparse, as from thermodynamic stability wells which would partition the fold space. We speculate that the idea of continuum will become more apparent as a larger number of new structures are solved by structural genomics projects[31]. In any case, the classification of structures into folds is probably a valuable and practical way of describing the fold space. When the fold space is continuous, this necessitates some arbitrary classification decisions, which may in fact not be completely reproducible by any automated approach.

## Conclusion

The results of this comprehensive comparison of VAST and SHEBA with the SCOP classification demonstrate that these two methods in their present form can reproduce at best 75% of the SCOP fold classification (for 1% false positive rate). Our detailed study of over 20 million pairs of protein domains underlines the difficulties encountered by automatic methods analyzing a classification of protein structures. A major difficulty arises from structural variation, which naturally accompanies amino acid sequence divergence, within the core of a defined fold. When severe enough, this can produce false negatives. When common cores of different folds are too similar, false positives result. Another, though less common, difficulty also arises when a motif is repeated several times within a single domain and in variable numbers. When the defining "common core" corresponds to only a small part of a whole structure, when the core is decorated extensively, automatic recognition of its similarity to other fold members becomes difficult. These divergences suggest a continuous rather than a discrete protein fold space, further complicating the problem of automatic classification. Clearly, improved algorithms of comparison must be developed and/or other types of classifications must be considered, and will be considered in future work.

## Methods

### Structural comparison methods

VAST is a method to superimpose and compare protein 3D structures. It consists of a two stage procedure. The first stage is based on a high-level description of protein structures. Secondary Structure Elements (SSEs) are represented by vectors and an algorithm based on a maximum clique search which finds the best one-to-one correspondence of a set of vectors in a query structure to a set of vectors in a target structure. Special care is paid to the significance of the one-to-one correspondence found between the two 3D structures. The method calculates the probability of generating a similar one-to-one correspondence by chance, and then correspondences are ranked and selected according to the value of this probability. Results of the first stage are used as seeds for the second stage.

In the second stage proteins are described using the alpha carbons (CAs) of the residues. The algorithm, based on a Gibbs-Monte Carlo procedure, tries to extend alignment of the initial seed to CAs belonging to the connecting loops. Usually, one wants to find the alignment that includes the maximum number of CAs yet with the smallest root mean square deviation (RMSD) value possible. Unfortunately, there is a correlation between the number of CAs included in the alignment and the value of RMSD: the larger the number of residues the higher the resulting RMSD value. The algorithm, in this second stage intended to solve this problem by answering questions such as: which alignment, one having 60 superimposed residues with a RMSD of 2.0, or one having 80 superimposed residues with a RMSD of 2.5 is the best one? This question is settled by choosing the alignment least likely to occur by chance, based on a *Z*-score calculation with respect to random distributions of the RMSD (see Appendix for more details). Please note that the VAST program we use is homologous to the version that can be downloaded at NCBI[37] (they both descent from a common ancestor[5,6]). The original VAST source code includes S+ subroutines. In this version, these subroutines were re-implemented in C language and regular PDB files can be used as input. It can be downloaded at VAST INRA server [38]. No other changes were made from the original version.

SHEBA is a protein structure comparison program which performs pairwise protein structure alignment in two steps. The initial alignment is made by maximizing the weighted sum of scores for the sequence homology, secondary structural similarity, and the similarity of the environment profile. The environment profile includes the solvent accessibility and polarity of the atoms around a given residue. The alignment is then iteratively refined in the second step, in which a new alignment is obtained from the three-dimensionally superimposed structures based on the current alignment, using a dynamic programming procedure that maximizes the number of residue pairs for which the CAs distance is less than 3.5 Å.

For each pair of proteins compared, SHEBA computes the *m *score defined as the number of matched residues divided by the mean length of the two protein domains. When one protein is compared to each protein in a database of target proteins, SHEBA also computes the *Zscore *computed from the score *m *as

Zscore=m−<m>σ(m)
 MathType@MTEF@5@5@+=feaafiart1ev1aaatCvAUfKttLearuWrP9MDH5MBPbIqV92AaeXatLxBI9gBaebbnrfifHhDYfgasaacH8akY=wiFfYdH8Gipec8Eeeu0xXdbba9frFj0=OqFfea0dXdd9vqai=hGuQ8kuc9pgc9s8qqaq=dirpe0xb9q8qiLsFr0=vr0=vr0dc8meaabaqaciaacaGaaeqabaqabeGadaaakeaacqWGAbGwcqWGZbWCcqWGJbWycqWGVbWBcqWGYbGCcqWGLbqzcqGH9aqpdaWcaaqaaiabd2gaTjabgkHiTiabgYda8iabd2gaTjabg6da+aqaaGGaciab=n8aZnaabmaabaGaemyBa0gacaGLOaGaayzkaaaaaaaa@4059@

where <*m*> and σ*(m) *are the mean and the standard deviation of the scores *m *between the same query domain and all other target domains in the database. SHEBA source code can be downloaded at SHEBA server[39].

### Analysis of structure comparison methods

We consider a set of protein structural domains, *D*, and a collection of *N *folds {*F*_*i*_} of the SCOP classification. Structural similarity of a query domain *q *to a target domain *t *is declared when *q *and *t *are members of the same SCOP fold. In other words, given a query *q*, we say that *q *is *similar to *(or *SCOP-similar to*) another domain *t *if and only if *q *∈ *F*_*i *_and *t *∈ *F*_*i*_, for some SCOP fold *F*_*i*_. Under this definition, structural similarity is an all-or-none phenomenon, as judged by SCOP, used as the reference.

Structure comparison methods are said to detect the structural similarity between a query domain *q *and a target domain *t *when the value of the computed similarity score *S*(*q*,*t*) (*Pcli *for VAST and *Zscore *for SHEBA) is above a pre-specified cutoff value. Formally, domain *q *is *detected as structurally similar *to *t *if and only if *S*(*q*, *t*) ≥ *c*, for some fixed cutoff value *c*.

We proceed as follow. First, the similarity scores *S*(*q*,*t*) for every *q *∈ *D *and every *t *∈ *D*, are calculated by VAST and SHEBA. Then, the overall accuracy of detection of structural similarity, compared to SCOP fold similarity is evaluated using ROC methodology, for structure comparison methods VAST and SHEBA, excluding similarity scores for *q*≡*t*. Finally, divergences between structural similarities measured by either structure comparison methods, and the SCOP classification are investigated using a heat map representation of the confusion matrix.

### ROC analysis

The four possible outcomes for a particular domain *q *evaluated against a particular target domain *t *are summarized in the Table 3.

The True Positive Rate, *TPR(c)*, the overall rate that a domain *q *is correctly detected to be similar to another domain is calculated first by comparing that domain to all other domains in its fold, then averaging this rate over all domains in the dataset *D*. Formally,

TPR(c)=1M∑i=1N∑q∈Fi∑t∈Fi,t≠qI(S(q,t)≥c)(ni−1)     (1)
 MathType@MTEF@5@5@+=feaafiart1ev1aaatCvAUfKttLearuWrP9MDH5MBPbIqV92AaeXatLxBI9gBaebbnrfifHhDYfgasaacH8akY=wiFfYdH8Gipec8Eeeu0xXdbba9frFj0=OqFfea0dXdd9vqai=hGuQ8kuc9pgc9s8qqaq=dirpe0xb9q8qiLsFr0=vr0=vr0dc8meaabaqaciaacaGaaeqabaqabeGadaaakeaacqWGubavcqWGqbaucqWGsbGudaqadaqaaiabdogaJbGaayjkaiaawMcaaiabg2da9maalaaabaGaeGymaedabaGaemyta0eaamaaqahabaWaaabuaeaadaWcaaqaamaaqafabaGaeeysaK0aaeWaaeaacqWGtbWudaqadaqaaiabdghaXjabcYcaSiabdsha0bGaayjkaiaawMcaaiabgwMiZkabdogaJbGaayjkaiaawMcaaaWcbaGaemiDaqNaeyicI4SaemOray0aaSbaaWqaaiabdMgaPbqabaWccqGGSaalcqWG0baDcqGHGjsUcqWGXbqCaeqaniabggHiLdaakeaadaqadaqaaiabd6gaUnaaBaaaleaacqWGPbqAaeqaaOGaeyOeI0IaeGymaedacaGLOaGaayzkaaaaaaWcbaGaemyCaeNaeyicI4SaemOray0aaSbaaWqaaiabdMgaPbqabaaaleqaniabggHiLdaaleaacqWGPbqAcqGH9aqpcqaIXaqmaeaacqWGobGta0GaeyyeIuoakiaaxMaacaWLjaWaaeWaaeaacqaIXaqmaiaawIcacaGLPaaaaaa@6851@

where *M *is the total number of structural domains in the dataset *D*, *N *is the number of folds (we consider only folds with *n*_*i *_>1), *n*_*i *_is the number of domains within a fold *F*_*i*_, and I(≺) is the indicator function, i.e. I(TRUE) = 1 and I(FALSE) = 0. *TPR(c) *is also the sensitivity of the method for SCOP.

Likewise, the False Positive Rate, the rate at which a domain *q *is falsely detected to be similar to another domain, is

FPR(c)=1M∑i=1N∑q∈Fi∑t∉FiI(S(q,t)≥c)M−ni     (2)
 MathType@MTEF@5@5@+=feaafiart1ev1aaatCvAUfKttLearuWrP9MDH5MBPbIqV92AaeXatLxBI9gBaebbnrfifHhDYfgasaacH8akY=wiFfYdH8Gipec8Eeeu0xXdbba9frFj0=OqFfea0dXdd9vqai=hGuQ8kuc9pgc9s8qqaq=dirpe0xb9q8qiLsFr0=vr0=vr0dc8meaabaqaciaacaGaaeqabaqabeGadaaakeaacqWGgbGrcqWGqbaucqWGsbGudaqadaqaaiabdogaJbGaayjkaiaawMcaaiabg2da9maalaaabaGaeGymaedabaGaemyta0eaamaaqahabaWaaabuaeaadaWcaaqaamaaqafabaGaeeysaK0aaeWaaeaacqWGtbWudaqadaqaaiabdghaXjabcYcaSiabdsha0bGaayjkaiaawMcaaiabgwMiZkabdogaJbGaayjkaiaawMcaaaWcbaGaemiDaqNaeyycI8SaemOray0aaSbaaWqaaiabdMgaPbqabaaaleqaniabggHiLdaakeaacqWGnbqtcqGHsislcqWGUbGBdaWgaaWcbaGaemyAaKgabeaaaaaabaGaemyCaeNaeyicI4SaemOray0aaSbaaWqaaiabdMgaPbqabaaaleqaniabggHiLdaaleaacqWGPbqAcqGH9aqpcqaIXaqmaeaacqWGobGta0GaeyyeIuoakiaaxMaacaWLjaWaaeWaaeaacqaIYaGmaiaawIcacaGLPaaaaaa@614B@

The specificity of the method for SCOP is [1 - *FPR*(*c*)].

### The ROC curve and the area under the ROC curve

The ROC curve is obtained by plotting the True Positive Rate *TPR*(*c*) against the False Positive Rate *FPR*(*c*), for the entire range of possible cutoff values, *c*. On this plot, the line through the origin with slope 1 would correspond to the performance of a similarity detection based on a random similarity score. A method which detects SCOP similarity better than randomly must show a ROC curve situated above this diagonal. The overall performance of either VAST or SHEBA in detecting SCOP fold similarity can be measured by the area under the ROC curve (*AUC*)[21,22], where a perfect detection method would yield *AUC *= 1 and a random prediction *AUC *= 0.5. The area is estimated using the trapezoid integration rule. Strictly speaking, we are presenting an average of the ROC curves for each individual fold-recognition problem, where the average is taken with the cutoff value, *c*, in common.

### Confusion matrix heat map

The performance of a similarity detection method can be studied within specific folds or fold pairs. Thus, we define a fold specific false positive rate between two different folds, *FPR*_*i*,*j*_(*c*) as the rate at which query domains in *F*_*i *_are detected to be similar to target domains in *F*_*j*_. We estimate this rate from our data as

FPRi,j(c)=∑q∈Fi∑t∈FjI(S(q,t)≥c)/ninj     (3)
 MathType@MTEF@5@5@+=feaafiart1ev1aaatCvAUfKttLearuWrP9MDH5MBPbIqV92AaeXatLxBI9gBaebbnrfifHhDYfgasaacH8akY=wiFfYdH8Gipec8Eeeu0xXdbba9frFj0=OqFfea0dXdd9vqai=hGuQ8kuc9pgc9s8qqaq=dirpe0xb9q8qiLsFr0=vr0=vr0dc8meaabaqaciaacaGaaeqabaqabeGadaaakeaadaWcgaqaaiabdAeagjabdcfaqjabdkfasnaaBaaaleaacqWGPbqAcqGGSaalcqWGQbGAaeqaaOWaaeWaaeaacqWGJbWyaiaawIcacaGLPaaacqGH9aqpdaaeqbqaamaaqafabaacbaGae8xsaK0aaeWaaeaacqWGtbWudaqadaqaaiabdghaXjabcYcaSiabdsha0bGaayjkaiaawMcaaiabgwMiZkabdogaJbGaayjkaiaawMcaaaWcbaGaemiDaqNaeyicI4SaemOray0aaSbaaWqaaiabdQgaQbqabaaaleqaniabggHiLdaaleaacqWGXbqCcqGHiiIZcqWGgbGrdaWgaaadbaGaemyAaKgabeaaaSqab0GaeyyeIuoaaOqaaiabd6gaUnaaBaaaleaacqWGPbqAaeqaaOGaemOBa42aaSbaaSqaaiabdQgaQbqabaaaaOGaaCzcaiaaxMaadaqadaqaaiabiodaZaGaayjkaiaawMcaaaaa@5D40@

We see this as a confusion in the similarity detection.

When *i *= *j*, we define the fold-specific true positive rate for domains in the same fold *F*_*i*_, estimated as

TPRi(c)=∑q∈Fi∑t∈Fi,t≠qI(S(q,t)≥c)/ni(ni−1)     (4)
 MathType@MTEF@5@5@+=feaafiart1ev1aaatCvAUfKttLearuWrP9MDH5MBPbIqV92AaeXatLxBI9gBaebbnrfifHhDYfgasaacH8akY=wiFfYdH8Gipec8Eeeu0xXdbba9frFj0=OqFfea0dXdd9vqai=hGuQ8kuc9pgc9s8qqaq=dirpe0xb9q8qiLsFr0=vr0=vr0dc8meaabaqaciaacaGaaeqabaqabeGadaaakeaadaWcgaqaaiabdsfaujabdcfaqjabdkfasnaaBaaaleaacqWGPbqAaeqaaOWaaeWaaeaacqWGJbWyaiaawIcacaGLPaaacqGH9aqpdaaeqbqaamaaqafabaacbaGae8xsaK0aaeWaaeaacqWGtbWudaqadaqaaiabdghaXjabcYcaSiabdsha0bGaayjkaiaawMcaaiabgwMiZkabdogaJbGaayjkaiaawMcaaaWcbaGaemiDaqNaeyicI4SaemOray0aaSbaaWqaaiabdMgaPbqabaWccqGGSaalcqWG0baDcqGHGjsUcqWGXbqCaeqaniabggHiLdaaleaacqWGXbqCcqGHiiIZcqWGgbGrdaWgaaadbaGaemyAaKgabeaaaSqab0GaeyyeIuoaaOqaaiabd6gaUnaaBaaaleaacqWGPbqAaeqaaOWaaeWaaeaacqWGUbGBdaWgaaWcbaGaemyAaKgabeaakiabgkHiTiabigdaXaGaayjkaiaawMcaaaaacaWLjaGaaCzcamaabmaabaGaeGinaqdacaGLOaGaayzkaaaaaa@6406@

The confusion matrix, defined by *TPR*_*i*_(*c*) for *i *= *j *and by *FPR*_*i*,*j*_(*c*) otherwise, for a particular value of *c*, can be visualized graphically as a heat map, with values of the matrix coded in grey scale (1 = black, 0 = white). To emphasize underlying patterns of confusion amongst the folds, the order of rows and columns (corresponding to the folds of SCOP) is permuted within fold class using hierarchical cluster analysis of the columns of the VAST heat map for class C and of the SHEBA heat map for the other classes. The hierarchical clustering was based on correlation between columns, and used Ward's method, as implemented in the Matlab Statistics toolbox (The Mathworks, Natick, MA). The same ordering of folds was then applied to the rows and columns of the confusion matrix heat map for both methods. Visually, the main diagonal of the heat map shows the agreement between SCOP and the structure comparison/similarity method, while the off-diagonal shows areas of confusion made by each structure comparison method in detecting SCOP fold similarity. The overall, global properties based on the entire set of SCOP folds may be appreciated by viewing the entire heat map, while particular properties of subsets of folds may be viewed by zooming into particular areas. Sets of confused folds may be quickly recognized and identified in this manner.

The *TPR*(*c*)*(eq. 1) *can be computed as the average of the *TPR*_*i*_*(c) *weighted by fold size, 1M∑i=1NniTPRi(c)
 MathType@MTEF@5@5@+=feaafiart1ev1aaatCvAUfKttLearuWrP9MDH5MBPbIqV92AaeXatLxBI9gBaebbnrfifHhDYfgasaacH8akY=wiFfYdH8Gipec8Eeeu0xXdbba9frFj0=OqFfea0dXdd9vqai=hGuQ8kuc9pgc9s8qqaq=dirpe0xb9q8qiLsFr0=vr0=vr0dc8meaabaqaciaacaGaaeqabaqabeGadaaakeaadaWcaaqaaiabigdaXaqaaiabd2eanbaadaaeWbqaaiabd6gaUnaaBaaaleaacqWGPbqAaeqaaOGaemivaqLaemiuaaLaemOuai1aaSbaaSqaaiabdMgaPbqabaGcdaqadaqaaiabdogaJbGaayjkaiaawMcaaaWcbaGaemyAaKMaeyypa0JaeGymaedabaGaemOta4eaniabggHiLdaaaa@406D@. Likewise the *FPR*(*c*)*(eq. 2) *can be computed as the fold size-weighted average of the fold specific false positive rate, 1M∑i=1NniM−ni(∑j=1;j≠iNnjFPRi,j(c))
 MathType@MTEF@5@5@+=feaafiart1ev1aaatCvAUfKttLearuWrP9MDH5MBPbIqV92AaeXatLxBI9gBaebbnrfifHhDYfgasaacH8akY=wiFfYdH8Gipec8Eeeu0xXdbba9frFj0=OqFfea0dXdd9vqai=hGuQ8kuc9pgc9s8qqaq=dirpe0xb9q8qiLsFr0=vr0=vr0dc8meaabaqaciaacaGaaeqabaqabeGadaaakeaadaWcaaqaaiabigdaXaqaaiabd2eanbaadaaeWbqaamaalaaabaGaemOBa42aaSbaaSqaaiabdMgaPbqabaaakeaacqWGnbqtcqGHsislcqWGUbGBdaWgaaWcbaGaemyAaKgabeaaaaaabaGaemyAaKMaeyypa0JaeGymaedabaGaemOta4eaniabggHiLdGcdaqadaqaamaaqahabaGaemOBa42aaSbaaSqaaiabdQgaQbqabaGccqWGgbGrcqWGqbaucqWGsbGudaWgaaWcbaGaemyAaKMaeiilaWIaemOAaOgabeaakmaabmaabaGaem4yamgacaGLOaGaayzkaaaaleaacqWGQbGAcqGH9aqpcqaIXaqmcqGG7aWocqWGQbGAcqGHGjsUcqWGPbqAaeaacqWGobGta0GaeyyeIuoaaOGaayjkaiaawMcaaaaa@585B@.

The true positive rate averaged over a subset of folds, *S*, is defined by

TPRS(c)=1MS∑i∈Sni⋅TPRi,
 MathType@MTEF@5@5@+=feaafiart1ev1aaatCvAUfKttLearuWrP9MDH5MBPbIqV92AaeXatLxBI9gBaebbnrfifHhDYfgasaacH8akY=wiFfYdH8Gipec8Eeeu0xXdbba9frFj0=OqFfea0dXdd9vqai=hGuQ8kuc9pgc9s8qqaq=dirpe0xb9q8qiLsFr0=vr0=vr0dc8meaabaqaciaacaGaaeqabaqabeGadaaakeaacqWGubavcqWGqbaucqWGsbGudaWgaaWcbaGaem4uamfabeaakmaabmaabaGaem4yamgacaGLOaGaayzkaaGaeyypa0ZaaSaaaeaacqaIXaqmaeaacqWGnbqtdaWgaaWcbaGaem4uamfabeaaaaGcdaaeqbqaaiabd6gaUnaaBaaaleaacqWGPbqAaeqaaOGaeyyXICTaemivaqLaemiuaaLaemOuai1aaSbaaSqaaiabdMgaPbqabaGccqGGSaalaSqaaiabdMgaPjabgIGiolabdofatbqab0GaeyyeIuoaaaa@4A67@

where *M*_*s *_is the total number of domains represented in set *S*. Likewise, the false positive rate averaged over a subset of folds is defined

FPRS(c)=1MS∑i∈SniMS−ni(∑j∈S;j≠injFPRi,j(c)).
 MathType@MTEF@5@5@+=feaafiart1ev1aaatCvAUfKttLearuWrP9MDH5MBPbIqV92AaeXatLxBI9gBaebbnrfifHhDYfgasaacH8akY=wiFfYdH8Gipec8Eeeu0xXdbba9frFj0=OqFfea0dXdd9vqai=hGuQ8kuc9pgc9s8qqaq=dirpe0xb9q8qiLsFr0=vr0=vr0dc8meaabaqaciaacaGaaeqabaqabeGadaaakeaacqWGgbGrcqWGqbaucqWGsbGudaWgaaWcbaGaem4uamfabeaakmaabmaabaGaem4yamgacaGLOaGaayzkaaGaeyypa0ZaaSaaaeaacqaIXaqmaeaacqWGnbqtdaWgaaWcbaGaem4uamfabeaaaaGcdaaeqbqaamaalaaabaGaemOBa42aaSbaaSqaaiabdMgaPbqabaaakeaacqWGnbqtdaWgaaWcbaGaem4uamfabeaakiabgkHiTiabd6gaUnaaBaaaleaacqWGPbqAaeqaaaaaaeaacqWGPbqAcqGHiiIZcqWGtbWuaeqaniabggHiLdGcdaqadaqaamaaqafabaGaemOBa42aaSbaaSqaaiabdQgaQbqabaGccqWGgbGrcqWGqbaucqWGsbGudaWgaaWcbaGaemyAaKMaeiilaWIaemOAaOgabeaakmaabmaabaGaem4yamgacaGLOaGaayzkaaaaleaacqWGQbGAcqGHiiIZcqWGtbWucqGG7aWocqWGQbGAcqGHGjsUcqWGPbqAaeqaniabggHiLdaakiaawIcacaGLPaaacqGGUaGlaaa@63A9@

A *fold confusion index *may be defined as the ratio *FPR*_*S*_/*TPR*_*S *_for a set *S *of folds. When the index has a value near 1.0, it means that domains in the same fold are no more distinguishable than domains in different folds, using that particular cutoff value.

An alternate, but straightforward definition of the *TPR(c) *could be obtained by counting all correctly detected similar-appearing pairs divided by the total number of pairs in the same SCOP folds. This alternate definition would in fact weight the fold-specific *TPR*_*i*_*(c) (eq. 4) *according to the square of the fold size, thus over-weighting the domains in large folds compared to those in small folds. Our preferred definition, given in the equation above, weights each domain, not each domain pair, equally. The confusion matrix computed for this study and the corresponding MATLAB code is available at MSCL server [40].

### Datasets

The set of SCOP domains considered here are drawn from ASTRAL[41] version 1.63, with less that 40% pairwise sequence identity. The total number of domains in that sample is 4948, classified by SCOP into 740 folds. As more than one domain is required to evaluate the fold-specific *TPR*_*i*_, we study only the reduced data set of 468 folds containing 2 or more domains, which together contain *M *= 4676 domains. The sum of the squares of their respective content ∑ini2
 MathType@MTEF@5@5@+=feaafiart1ev1aaatCvAUfKttLearuWrP9MDH5MBPbIqV92AaeXatLxBI9gBaebbnrfifHhDYfgasaacH8akY=wiFfYdH8Gipec8Eeeu0xXdbba9frFj0=OqFfea0dXdd9vqai=hGuQ8kuc9pgc9s8qqaq=dirpe0xb9q8qiLsFr0=vr0=vr0dc8meaabaqaciaacaGaaeqabaqabeGadaaakeaadaaeqbqaaiabd6gaUnaaDaaaleaacqWGPbqAaeaacqaIYaGmaaaabaGaemyAaKgabeqdcqGHris5aaaa@33FE@ is 226,900. The folds fall into classes A (all alpha helix proteins), B (all beta sheet proteins), C (alpha and beta proteins, alpha/beta), D(alpha and beta proteins, alpha+beta), E(multi-domain proteins, alpha and beta), F(membrane and cell surface proteins and peptides) and G(small proteins) with 97, 78, 94, 139, 18, 17 and 25 folds each, respectively. The classes hold 844, 1091, 1330, 1070, 80, 67 and 194 domains each respectively.

All domain pairs drawn from the reduced dataset were compared by both VAST and SHEBA, corresponding to a total number of pairs of M*(*M *- 1) = 21,860,300, excluding identity pairs. The calculations were made using the high-performance computational capabilities of the Biowulf PC/Linux cluster at the National Institutes of Health, Bethesda, Md.[42]. Data resulting from the computations were used to produce two large matrices containing the *Zscore*, the *Pcli *score, respectively. For pairs of domains for which VAST could not assign a quantitative measure of similarity the *Pcli *value was arbitrarily set to -10.

## Abbreviations

AUC Area Under the ROC Curve

CA Carbon Alpha

CATH Hierarchical classification of protein domain structures, which clusters proteins at four major levels, Class(**C**), Architecture(**A**), Topology(**T**) and Homologous superfamily (**H**).

DALI Distance mAtrix aLIgnment

FPR False Positive Rate

NCBI National Center for Biotechnology Information

PDB Protein Data Bank

RMSD Root Mean Square Deviation

ROC Receiver Operating Characteristic

SCOP Structural Classification of Proteins

SHEBA Structural Homology by Environment-Based Alignment

SSE Secondary Structure Element

TPR True Positive Rate

VAST Vector Alignment Search Tool

## Authors' contributions

VS, PM – execution of pairwise comparisons using VAST on Biowulf computer, application of ROC methodology, development of confusion matrix heat maps, statistical analysis JFG, JG – development of VAST program, interpretation of confused structure pairs CHT, BKL – development of SHEBA program, and pairwise comparisons, 3D structure visualization, interpretation of confused structure pairs

## Appendix. VAST statistics: calculation of Pcli

In the first stage of VAST we consider a "high" level description of proteins. Proteins are represented by their secondary structure elements (SSEs), more specifically by the endpoints of vectors going through these SSEs. The basic task of the algorithm is to find the best 3D common substructure.

A 3D common substructure is formally defined as a one-to-one correspondence between a subset of SSE vectors in the first protein and a subset of the SSE vectors in the second protein. This correspondence respects the type of SSE (i.e., helices are only paired with helices and strands with strands) and the topology. A correspondence {(*i*,*k*),(*j*,*l*)} of SSEs *i *and *j *in the first protein with SSEs *j *and *k *in the second, is said to respect the topology when *i*<*j *implies *k*<*l*. For instance, if SSEs 1 and 2 in the first protein and SSEs 4 and 7 in the second protein are paired 1–4 and 2–7, the correspondence respects the topology. The correspondence 1–7 and 2–4 does not.

### Computation of the score for a common 3D substructure

The problem of searching for 3D common substructures is next transformed into a graph theory problem. A "comparison" graph is formed whose vertices are made of pairs of vectors, one from each protein to be compared. Two such vertices are connected by an edge if the two vectors in the first protein have the same relative orientation and spacing, within some tolerance, as the two vectors in the second. Each edge is labeled by a score *s*, reflecting the quality of the superimposition of the 2 pairs of vectors. Finding the best 3D common substructure is solved by finding the clique (i.e., a subgraph for which each vertex is linked with all others) with the best overall score. The overall score is defined as a normalized sum of scores for all the edges within the clique. The *n *vertices of a clique (an *n*-clique) are labeled by *i *and *j*, and the overall score is defined:

score(n−clique)=2n∑i=1n∑j>1nSij     (1)
 MathType@MTEF@5@5@+=feaafiart1ev1aaatCvAUfKttLearuWrP9MDH5MBPbIqV92AaeXatLxBI9gBaebbnrfifHhDYfgasaacH8akY=wiFfYdH8Gipec8Eeeu0xXdbba9frFj0=OqFfea0dXdd9vqai=hGuQ8kuc9pgc9s8qqaq=dirpe0xb9q8qiLsFr0=vr0=vr0dc8meaabaqaciaacaGaaeqabaqabeGadaaakeaacqWGZbWCcqWGJbWycqWGVbWBcqWGYbGCcqWGLbqzdaqadaqaaiabd6gaUjabgkHiTiabdogaJjabdYgaSjabdMgaPjabdghaXjabdwha1jabdwgaLbGaayjkaiaawMcaaiabg2da9maalaaabaGaeGOmaidabaGaemOBa4gaamaaqahabaWaaabCaeaacqWGtbWudaWgaaWcbaGaemyAaKMaemOAaOgabeaaaeaacqWGQbGAcqGH+aGpcqaIXaqmaeaacqWGUbGBa0GaeyyeIuoaaSqaaiabdMgaPjabg2da9iabigdaXaqaaiabd6gaUbqdcqGHris5aOGaaCzcaiaaxMaadaqadaqaaiabigdaXaGaayjkaiaawMcaaaaa@58D8@

### Computation of the score for a 2-clique

The 2-clique score, *s*, is computed from the RMSD of the superimposition of the two corresponding pairs of vectors. The RMSD is normalized to an observed distribution of such values obtained from a large sample of random pairings of 2 SSEs from pairs of proteins drawn from a representative set of proteins. This empirical distribution represents the behavior of random pairing of two secondary structures, when there is presumably no overall structural similarity between the two proteins. By definition, we set *s *= -log_10 _(*P*) where *P *is the empirical cumulative distribution function (cdf) value associated with the particular RMSD value. For instance if the RMSD is found to be 5.8 Å when the 2 pairs of vectors are superimposed, the probability *P *that the RMSD is less than or equal to 5.8 can be read off the curve as, say, *P *= 0.2. The score is then defined as minus the log of this probability: *s *= -log_10_(.2) = 0.7. Therefore the smaller the RMSD between the 2 pairs of SSEs the larger the resulting score.

### Computing the probability distribution for the best n-clique score

In the previous section, *P *represented the cdf value of the RMSD of a random 2-clique. Accordingly, *P *has a uniform distribution and its score (-log_10_(*P*)/2.303) has a negative exponential distribution, after dividing by the natural logarithm of 10, ln(10) ≈ 2.303. Rewriting in terms of natural logarithms, we have *score*(*n *- *clique*) = 22.303n∑i=1n∑j>in−ln⁡(Pij)
 MathType@MTEF@5@5@+=feaafiart1ev1aaatCvAUfKttLearuWrP9MDH5MBPbIqV92AaeXatLxBI9gBaebbnrfifHhDYfgasaacH8akY=wiFfYdH8Gipec8Eeeu0xXdbba9frFj0=OqFfea0dXdd9vqai=hGuQ8kuc9pgc9s8qqaq=dirpe0xb9q8qiLsFr0=vr0=vr0dc8meaabaqaciaacaGaaeqabaqabeGadaaakeaadaWcaaqaaiabikdaYaqaaiabikdaYiabc6caUiabiodaZiabicdaWiabiodaZiabd6gaUbaadaaeWbqaamaaqahabaGaeyOeI0IagiiBaWMaeiOBa42aaeWaaeaacqWGqbaudaWgaaWcbaGaemyAaKMaemOAaOgabeaaaOGaayjkaiaawMcaaaWcbaGaemOAaOMaeyOpa4JaemyAaKgabaGaemOBa4ganiabggHiLdaaleaacqWGPbqAcqGH9aqpcqaIXaqmaeaacqWGUbGBa0GaeyyeIuoaaaa@4B71@. Since this is a sum of *n*(*n *- 1)/2 independent exponential variates, multiplied by the factor 2/2.303*n*, it follows a classical Gamma distribution with parameters α = *n*(*n *- 1)/2 and β = 2/2.303*n*.

For example, assume that we found a common 3D substructure between 2 proteins, and it is a 6-clique with a score of 9.6. In order to determine the significance of this score one must compare it with a distribution of scores for randomly generated 6-cliques. The mean of the 6-clique score distribution is given by α·β = (6-1)/2.303 ≈ 2.171, 5 times larger than the mean of a 2-clique score, 0.434. As *n *grows, the distributions become broader and more symmetric. Having calculated the score probability density distribution for a 6-clique, it is then easy to estimate the significance of the score obtained for our 6-clique found while comparing the 2 proteins. The corresponding cumulative probability distribution is calculated and used to compute the probability that a random score is = 9.6. This probability is written Q(*s*,*n*) = 1 - P(*s*,*n*) where *s *and *n *refer respectively to the score and the number of elements of the clique.

### Number of n-cliques that can be generated with a particular pair of proteins

For the sake of simplicity, we consider proteins having only one type of SSE, for instance, helices (when both proteins contain helices and strands, the problem of estimating *C*(*n*, *N*1, *N*2) can be formulated as a substring matching problem, for which a fast recursive algorithm exists). If we compare 2 proteins having 6 helices and we find that the best clique has 6 elements, there is only one possibility of generating this 6-clique. On the other hand, if both proteins have 12 helices, the number of 6-cliques that can be generated is given by (126)(126)=(12!6!6!)2
 MathType@MTEF@5@5@+=feaafiart1ev1aaatCvAUfKttLearuWrP9MDH5MBPbIqV92AaeXatLxBI9gBaebbnrfifHhDYfgasaacH8akY=wiFfYdH8Gipec8Eeeu0xXdbba9frFj0=OqFfea0dXdd9vqai=hGuQ8kuc9pgc9s8qqaq=dirpe0xb9q8qiLsFr0=vr0=vr0dc8meaabaqaciaacaGaaeqabaqabeGadaaakeaadaqadaqaauaabeqaceaaaeaacqaIXaqmcqaIYaGmaeaacqaI2aGnaaaacaGLOaGaayzkaaWaaeWaaeaafaqabeGabaaabaGaeGymaeJaeGOmaidabaGaeGOnaydaaaGaayjkaiaawMcaaiabg2da9maabmaabaWaaSaaaeaacqaIXaqmcqaIYaGmcqGGHaqiaeaacqaI2aGncqGGHaqicqaI2aGncqGGHaqiaaaacaGLOaGaayzkaaWaaWbaaSqabeaacqaIYaGmaaaaaa@3F82@ = 853776 cliques. It is thus much more probable to observe, just by chance, a 6-clique with a score of 9.6 with 2 proteins having 12 helices rather than with 2 proteins having 6 helices. Let us call C(*n*, *N1*, *N2*) the number of n-cliques that can be generated with a pair of proteins having respectively N1 and N2 SSEs.

### Calculation of Pcli for the best clique

Because C(*n*, *N1*, *N2*) independent *n*-cliques can be generated, in the best *n*-clique for randomly paired proteins, we may observe a score larger than one would expect from the empirical distribution. The corrected significance, termed EPcli, is the probability that any random score in C(*n*, *N1*, *N2*) trials would exceed our observed score *s*. The probability of finding one value s* higher than observed value *s*, by chance alone is

EPcli=Pr⁡{s*≥s for any trial}=1−[1−Q(s,n)]C(n,N1,N2)=1−[1−C(n,N1,N2)⋅Q(s,n)+higher order terms]≈C(n,N1,N2)⋅Q(s,n)     (2)
 MathType@MTEF@5@5@+=feaafiart1ev1aaatCvAUfKttLearuWrP9MDH5MBPbIqV92AaeXatLxBI9gBaebbnrfifHhDYfgasaacH8akY=wiFfYdH8Gipec8Eeeu0xXdbba9frFj0=OqFfea0dXdd9vqai=hGuQ8kuc9pgc9s8qqaq=dirpe0xb9q8qiLsFr0=vr0=vr0dc8meaabaqaciaacaGaaeqabaqabeGadaaakeaafaqaaeabbaaaaeaacqWGfbqrcqWGqbaucqWGJbWycqWGSbaBcqWGPbqAcqGH9aqpcyGGqbaucqGGYbGCdaGadaqaaiabdohaZjabcQcaQiabgwMiZkabdohaZjabbccaGiabbAgaMjabb+gaVjabbkhaYjabbccaGiabbggaHjabb6gaUjabbMha5jabbccaGiabbsha0jabbkhaYjabbMgaPjabbggaHjabbYgaSbGaay5Eaiaaw2haaaqaaiabg2da9iabigdaXiabgkHiTmaadmaabaGaeGymaeJaeyOeI0Iaemyuae1aaeWaaeaacqWGZbWCcqGGSaalcqWGUbGBaiaawIcacaGLPaaaaiaawUfacaGLDbaadaahaaWcbeqaaiabdoeadnaabmaabaGaemOBa4MaeiilaWIaemOta4KaeGymaeJaeiilaWIaemOta4KaeGOmaidacaGLOaGaayzkaaaaaaGcbaGaeyypa0JaeGymaeJaeyOeI0YaamWaaeaacqaIXaqmcqGHsislcqWGdbWqdaqadaqaaiabd6gaUjabcYcaSiabd6eaojabigdaXiabcYcaSiabd6eaojabikdaYaGaayjkaiaawMcaaiabgwSixlabdgfarnaabmaabaGaem4CamNaeiilaWIaemOBa4gacaGLOaGaayzkaaGaey4kaSIaeeiAaGMaeeyAaKMaee4zaCMaeeiAaGMaeeyzauMaeeOCaiNaeeiiaaIaee4Ba8MaeeOCaiNaeeizaqMaeeyzauMaeeOCaiNaeeiiaaIaeeiDaqNaeeyzauMaeeOCaiNaeeyBa0Maee4CamhacaGLBbGaayzxaaaabaGaeyisISRaem4qam0aaeWaaeaacqWGUbGBcqGGSaalcqWGobGtcqaIXaqmcqGGSaalcqWGobGtcqaIYaGmaiaawIcacaGLPaaacqGHflY1cqWGrbqudaqadaqaaiabdohaZjabcYcaSiabd6gaUbGaayjkaiaawMcaaaaacaWLjaGaaCzcamaabmaabaGaeGOmaidacaGLOaGaayzkaaaaaa@B0B6@

This approximation is valid when *C*(*n*, *N*1, *N*2)·*Q*(*s*, *n*)<<1. With the above definition of EPcli, notice that the smaller the value of EPcli the more significant the clique. Finally, we define

Pcli = -log_10_(EPcli).

### Remark on the calculation of Pcli

Two types of problems occur. The first one is related to the number of elements of the clique with respect to the number of secondary structure elements (SSEs) found in the 2 domains being compared. To illustrate let us consider the 7-element clique that is generated when comparing domains d1amx_ and d1h6fa_ (fold b.2) having 14 and 19 SSEs, respectively. The score of this 7-clique, calculated according to Eq. 1 is 8.6. The probability of generating a 7-clique having a score *s *= 8.6 is given by P(*s*, *n*)= 10^-5.3^. The number of 7-cliques that can be generated with the above two domains is C(*7, 14, 19*) = 10^+7^. This leads to an approximation of EPcli > 1, and hence *Pcli*<0. The match found between the 2 domains is thus not significant. The second problem occurs with small proteins having few (3 or 4) SSEs. As shown in Eq. 1 the score of a clique depends on the number of elements. Small cliques will have relatively small scores and will appear more likely to have been generated by chance than large scores. This is analogous to the problem of detecting similarities for small peptides with sequence comparison methods: although a perfect match might be found, the resulting score, due to the size of the query sequence, will always be small and thus will appear not significant.

## Supplementary Material

Additional File 1 • VAST and SHEBA heat maps • Complete heat map of VAST and SHEBA, obtained for a *Pcli *cutoff value of 2.5 and a *Zscore *cutoff value of 2.7, respectively. The cutoffs correspond to an overall average *FPR *of 0.01, and result in an overall average *TPR *of 0.616 and 0.748 for VAST and SHEBA respectively. The x (target folds) and y (query folds) axes of the heat maps are labeled by the SCOP folds, grouped into the different classes A, B, C, D, E, F and G. Each pixel within the heat maps represents a fold-specific true or false positive rate and takes value between 0 and 1. Diagonal and off-diagonal pixels correspond to fold-specific true positive rate *TPR*_*i*_(c) (eq. 4, see Methods) and fold-specific false positive rate *FPR*_*i*,*j*_(c) (eq. 3, see Methods) respectively. Click here for file

Additional File 2 • Fold-specific True Positive Rates (see Methods) at 1% False Positive Rate, for VAST and SHEBA, for 468 SCOP Folds in the order of the Heat Map. • Rows 1 to 7 correspond respectively to: the row number, the SCOP fold identifier, the number of domains within a fold, *TPR*_*i *_value obtained by the fold with VAST, *TPR*_*i *_value obtained by the fold with SHEBA, SCOP name of the fold, and SCOP description of the fold. Click here for file

## Figures and Tables

**Table 1:** Folds having domain pairs with undetected similarity by both VAST and SHEBA.

**Class**	**List of folds**
A	a.4(1576/13572), a.118(777/2550), a.39(282/1640), a.60(238/812), a.138(166/272), a.24(77/930), a.1(62/930), a.2(47/272), a.100(39/90), a.25(37/182), a.3(37/992), a.29(25/132), a.26(20/650), a.23(10/20), a.28(10/72), a.69(9/20), a.7(9/342), a.93(8/42), a.102(7/600), a.112(4/20), a.127(4/30), a.35(4/110), a.61(4/30), a.5(3/90), a.55(3/20), a.74(3/272), a.116(2/20), a.126(2/30), a.133(2/20), a.137(2/6), a.64(2/20), a.128(1/42), a.144(1/12), a.27(1/72), a.48(1/6), a.6(1/42).
B	b.1(2973/57840), b.40(1382/7482), b.34(436/2652), b.82(341/930), b.10(323/1640), b.2(164/702), b.29(163/1056), b.85(91/156), b.43(69/702), b.84(49/182), b.30(32/110), b.50(16/132), b.18(14/552), b.13(12/110), b.35(11/72), b.7(11/182), b.19(10/30), b.6(8/1406), b.80(8/110), b.92(7/56), b.3(6/110), b.60(6/420), b.106(5/6), b.52(4/132), b.49(3/12), b.58(3/20), b.21(2/6), b.45(2/12), b.53(2/6), b.83(2/2).
C	c.37(6218/14762), c.1(1152/32942), c.55(929/2756), c.26(255/1722), c.52(228/506), c.2(197/9702), c.23(161/4160), c.69(92/2550), c.94(90/600), c.66(87/1190), c.56(38/552), c.47(17/2550), c.58(16/110), c.92(16/110), c.3(13/2070), c.10(12/306), c.53(12/72), c.8(12/90), c.14(9/110), c.51(9/156), c.72(6/210), c.43(4/42), c.61(3/272), c.36(2/342), c.19(1/6), c.63(1/20), c.78(1/132), c.87(1/30), c.9(1/2), c.97(1/12).
D	d.58(2052/17556), d.92(235/552), d.3(221/380), d.142(164/380), d.15(104/3080), d.169(74/552), d.26(74/306), d.17(59/552), d.81(54/210), d.153(49/600), d.166(42/90), d.211(40/132), d.144(33/650), d.110(26/306), d.129(23/182), d.68(23/90), d.2(22/132), d.14(14/240), d.79(14/210), d.108(12/210), d.16(12/182), d.87(10/156), d.4(8/12), d.104(5/210), d.109(4/182), d.122(4/110), d.143(4/6), d.41(4/90), d.67(4/20), d.10(3/20), d.50(3/72), d.184(2/2), d.52(2/90), d.18(1/2), d.74(1/56), d.82(1/6).
E	e.8(110/182), e.26(3/6)
F	f.1(58/110), f.4(46/182), f.21(12/42), f.23(5/20), f.7(4/6).
G	g.3(357/1406), g.41(96/420), g.15(5/90), g.17(4/132), g.39(2/132).

Folds from classes A, B, C, D, E, F and G are reported in rows labeled by the name of the class. Reported folds within a given class are ordered by decreasing number of domain pairs with undetected similarity they contain. The number of such pairs within a fold and the total number of pairs are indicated for each fold in parenthesis. Similarity between domains of a pair was considered undetected when their *Pcli *and *Zscore *were below the 5% *FPR *cutoffs of 1 for *Pcli *and 1.6 for *Zscore*.

**Table 2:** Sets of folds confused by both VAST and SHEBA.

	Sets of confused folds, S	Number of domains in S	Sheba *FPR*_*S *_(%)	Sheba *TPR*_*S *_(%)	**Sheba FPR**_**S**_/**TPR**_**S **_**(%)**	Vast *FPR*_*S *_(%)	Vast *TPR*_*S *_(%)	**Vast FPR**_**S**_/**TPR**_**S **_**(%)**	Explanation for confusion
1	a.28, a.39	50	29	57	**51**	10	16	**64**	4 helix bundle up-and-down (a.28), and 4 helix array of 2 hairpins folds. Confusion is caused by match of helices oriented similarly. Folds confused mostly by SHEBA.
2	a.46, a.52	9	45	97	**46**	7	36	**20**	4 helix bundle left and right-handed super helix (a.46), and 4 helix right-handed super helix folds. Confusion is caused by match of helices oriented similarly. Folds confused mostly by SHEBA.
3	a.47, a.7	24	87	88	**98**	8	20	**40**	3 helix bundle (a.7) and 4 helix bundle (a.47) folds. Confusion due to match of very similar structure. Folds confused mostly by SHEBA.
4	b.68, b.69, b.66, b.67, b.70	45	92	98	**94**	40	83	**48**	Beta-propellers (repetitive 4-stranded blades) folds, of 4, 5, 6, 7 or 8 blades depending on the fold. Confusion is caused by match of several 4-stranded blades among domains of these folds.
5	b.1, b.2, b.3, b.7, b.12.	297	19	66	**29**	32	68	**48**	Beta sandwich folds of 7, 8, 9 stranded-sheet, with Greek-key topology. The motif causing the confusion among folds is a sandwich, which is rather well matched between domains of these folds.
6	b.24, b.71	24	69	97	**72**	27	93	**29**	Sandwich fold, with 10 strands in 2 sheets, and "folded meander topology" fold (b.24), and folded sheet with Greek-key topology. Confusion is due to match of parts of the sheets of the common core of these folds.
7	b.60, b.61	30	63	90	**70**	57	78	**74**	Closed barrel, with meander topology. Confusion caused by good match of between barrel motifs of the common core.
8	b.43, b.49, b.58, b.44	39	42	71	**59**	32	72	**44**	Folds of closed barrel with Greek-key topology. Confusion is due the match of substantial part of the barrel common core, among domains of these folds.
9	b.107, b.4	4	100	100	**100**	25	100	**25**	Sandwich fold (b.4), and closed barrel fold (b.107). Confusion is caused by the good match between a deformed barrel motif and a sandwich motif.
10	b.34, b.38	62	69	67	**103**	19	49	**39**	Barrel folds, with meander topology. Confusion is caused by the match between the barrel common cores.
11	b.38, b.56	12	52	100	**52**	65	93	**70**	Open barrel (b.38) and closed barrel (b.56) folds. Confusion is caused by the match of the barrel.
12	b.10, b.19, b.13, b.18, b.22, b.23	91	42	76	**55**	16	54	**29**	Folds with common core motif of beta sandwich; the 2 sheets are made of 8, 9 or 10 strands depending on the fold, and with jelly roll topology. The confusion among these folds is caused by the match of the strands of the beta sandwich common core.
13	c.1, c.6	185	62	75	**83**	78	87	**90**	TIM barrel (c.1) and variant of beta/alpha barrel, with closed parallel beta-sheet barrel (c.6) folds. Confusion is caused by the match of almost the whole TIM barrel.
14	c.8, c.98	14	50	75	**68**	30	54	**56**	3 layer beta/beta/alpha (c.8) and 3 layer alpha/beta/alpha (c.98) folds. Confusion is caused by the match between common beta/alpha layers.
15	c.84, c.95	19	65	91	**71**	55	92	**60**	3 layer alpha/beta/alpha of 4 strands (c.84), and of 5 strands (c.95) folds. Match of the 3 layer alpha/beta/alpha common core causes the confusion.
16	c.101, c.73, c.27	7	11	100	**11**	49	100	**49**	3 layer alpha/beta/alpha folds, with 5, 6 or 8 strands depending on the fold. Confusion is caused by the match of the 3 layer alpha/beta/alpha common core.
17	c.100, c.28, c.25, c.24, c.30, c.78, c.108, c.116, c.31, c.114, c.3, c.4, c.49, c.59, c.16, c.57, c.44, c.48, c.2, c.33, c.32, c.34, c.23, c.62, c.65, c.5	334	24	80	**31**	51	92	**56**	3 layer alpha/beta/alpha folds, with beta sheet of 4, 5, 6 or 7 strands depending of the fold. 3 layer beta/beta/alpha with central of 5 strands for c.3. Confusion among 3 layer alpha/beta/alpha folds is caused by the match of the 3 layer alpha/beta/alpha common core. Confusion between 3 layer alpha/beta/alpha and beta/beta/alpha is caused by the match of the 2 layer beta/alpha.
18	d.13, d.173	7	26	93	**28**	43	86	**50**	Fold containing the 3 layer alpha/beta/alpha common core (d.130 and unusual fold containing a common core of beta-alpha-beta-alpha-beta-alpha-beta (d.173). Confusion caused by the match of some strands and helices.
19	d.65, d.67	7	47	46	**102**	60	64	**93**	2 layer alpha/beta sandwich fold. Confusion caused by the match of 2 layer alpha/beta sandwich common core.
20	d.181, d.212	5	50	60	**83**	17	60	**28**	Folds containing beta-alpha-beta units. Confusion caused by match on the alpha/beta layers.
21	d.10, d.50	14	34	66	**51**	40	61	**66**	2 layer alpha/beta folds. Confusion caused by match on the 2 layer alpha/beta common cores.
22	d.140, d.68	12	34	68	**51**	40	52	**77**	Fold with 2 layer beta/alpha sandwich common core. Confusion is caused by match of the 2 layer beta/alpha sandwich.
23	d.151, d.160	7	75	100	**75**	58	100	**58**	Beta-sandwich; duplication of alpha+beta (d.151), 4 layers: alpha/beta/beta/alpha; mixed beta sheets (d.160) folds. Confusion due to match of the alpha beta sandwich.
24	d.95, d.206, d.64	12	18	96	**18**	34	79	**43**	2 layer alpha/beta sandwich folds. Confusion caused by the match of the 2 layer alpha/beta sandwich.
25	d.11, d.40	5	100	100	**100**	67	100	**67**	2 layer alpha/beta sandwich folds. Confusion caused by match of the 2 layer alpha/beta sandwich.
26	d.130, d.80, d.52	19	53	90	**59**	51	62	**82**	2 layer alpha/beta sandwich folds. Confusion is caused by the match of the 2 layer alpha/beta sandwich.
27	d.45, d.74, d.58, d.51, d.94, d.141, d.105	160	43	58	**74**	48	59	**81**	2 layer alpha/beta sandwich, and two beta-sheets and one alpha-helix packed around single core (d.141) folds. Confusion caused by match of the sheet and strands of the 2 layer alpha/beta sandwich core motif.
28	e.24, c.16, c.57, c.44, c.23, c.5	79	47	73	**64**	68	85	**80**	A domain component of a "multi-domain" domain of fold e.24 can matches the full domain of another fold which does not belong to the E class
29	e.4, c.48, c.2, c.32, c.33, c.34, c.23	178	35	74	**48**	74	87	**85**	A domain component of a "multi-domain" domain of fold e.4 matches the full domain of another fold which does not belong to the E class

Clusters of confused folds in VAST and SHEBA heat maps are reported. Rows 1 to 27 are intra-class clusters of confused folds found along the diagonal of the heat map. Only confusions in classes A, B, C and D are reported. Rows 28 and 29 are two off-diagonal clusters involving multi domains. Clusters and confused folds are listed in the order of appearance in the heat map. The heat maps of both methods obtained at 1% overall *FPR *were used to determined these clusters. Column 3 is the total number of domains within the set S. Columns 4 to 6 report the *FPR*_*S*_, *TPR*_*S *_(see Methods) and their ratios (in bold), for SHEBA, respectively, similarly, columns 7 to 9, report *FPR*_*S*_, *TPR*_*S *_and their ratios (in bold), for VAST, respectively.

**Table 3:** The four possible outcomes of ROC analysis for a particular domain.

	Domain *q *is in the same SCOP fold as *t*	Domain *q *is **not **in the same fold as *t*
Domain *q *is detected as similar to *t*	***True Positive***	***False Positive***
Domain *q *is **not **detected as similar to *t*	***False Negative***	***True Negative***

## References

[B1] Richardson JS (1981). The anatomy and taxonomy of protein structure. Advance protein chemistry.

[B2] Murzin AG, Brenner SE, Hubbard T, Chothia C (1995). SCOP: a structural classification of proteins database for investigation of sequences and structures. Journal of Molecular Biology.

[B3] Orengo C, Michie AD, Jones S, Jones DT, Swindells MB, Thornton JM (1997). CATH-a hierarchic classification of protein domains structures. Structures.

[B4] Chothia C, Lesk AM (1986). The relation between the divergence of sequence and structure in proteins. The EMBO journal.

[B5] Gibrat JF, Madej T, Bryant SS (1996). Surprising similarities in structure comparison. Current Opinion in Structural Biology.

[B6] Madej T, Gibrat JF, Bryant SH (1995). Threading a database of protein cores. Protein: Structure, Function, and Genetics.

[B7] Ortiz AR, Strauss C, Olmea O (2002). MAMMOTH (Matching Molecular Models Obtained from Theory): An automated method for model comparison. Protein Science.

[B8] Zemla A (2003). LGA: a method for finding 3D similarities in protein structures. Nucleic Acids Research.

[B9] Goldsmith-Fischman S, Honig B (2003). Structural genomics: computational methods for structure analysis.. Protein Science.

[B10] Koehl P (2001). Protein structure similarities. Current Opinion in Structural Biology.

[B11] Subbiah S, Laurents DV, Levitt M (1993). Structural similarity of DNA-binding domains of bacteriophage repressors and the globin core. Current Biology.

[B12] Shindyalov IN, Bourne PE (1998). Protein structure alignment by incremental combinatorial extension (CE) of the optimal path. Protein Engineering.

[B13] Harrison A, Pearl F, Mott R, Thornton J, Orengo C (2002). Quantifying the similarities within fold space. Journal of Molecular Biology.

[B14] Shapiro J, Brutlag D (2004). FoldMiner: Structural motif discovery using an improved superposition algorithm. Protein Science.

[B15] Yang AS, Honig B (2000). An integrated approach to the analysis and modeling of protein sequences and structures. Protein structural alignment and a quantitative measure for protein structural distance. Journal of Molecular Biology.

[B16] Jung J, Lee B (2000). Protein structure alignment using environmental profiles. Protein Engineering.

[B17] Ye Y, Godzik A (2004). Database searching by flexible protein structure alignment. Protein Science.

[B18] Shindyalov I, Bourne PE (2000). An alternative view of protein fold space. Proteins: Structure, Function and Genetics.

[B19] Holm L, Sander C (1993). Protein structure comparison by alignment of distance matrices. Journal of Molecular Biology.

[B20] Kolodny R, Koehl P, Levitt M (2005). Comprehensive evaluation of protein structure alignment methods: scoring by geometric measures. Journal of Molecular Biology.

[B21] Hanley JA, McNeil BJ (1982). The meaning of the area under the Receiver Operationg Characteristic (ROC) Curve. Radiology.

[B22] Bradley AP (1997). The use of the area under the ROC curve in the evaluation of machine learning algorithms. Pattern Recognition.

[B23] Sierk ML, Pearson WR (2004). Sensitivity and selectivity in protein structure comparison. Protein Science(2004).

[B24] Getz G, Vendruscolo M, Sachs D, Domany E (2002). Automated Assignment of SCOP and CATH Protein Structure Classifications from FSSP. Proteins: Structure, Function and Genetics.

[B25] Hadley C, Jones D (1999). A systematic comparison of protein structure classifications: SCOP, CATH and FSSP. Structures.

[B26] Day R, Beck D, Armen R, Daggett V (2003). A consensus view of fold space: combining SCOP, CATH, and Dali Domain Dictionnary. Protein Science.

[B27] Gerstein M, Levitt M (1998). Comprehensive assessment of automatic structural alignment against a manual standard, the SCOP classification of proteins. Protein Science.

[B28] Novotny M, Madsen D, Kleywegt GJ (2004). Evaluation of protein fold comparison servers. PROTEINS: Structure, Function and Bioinformatics.

[B29] Brenner SE, Chothia C, Hubbard TJP, Murzin AG (1996). Understanding protein structure: using SCOP for fold interpretation. Methods in Enzymology.

[B30] Kajava A (2002). What curves alpha-solenoids ? Evidence for an alpha-helical toroid structure of Rpn1 and Rpn2 proteins of the 26 S proteasome. The Journal of Biological Chemistry.

[B31] Hou J, Sims GE, Zhang C, Kim SH (2003). A global representation of the protein fold space. PNAS.

[B32] Hou J, Jun SR, Zhang C, Kim SH (2005). Global mapping of protein structure space and application in structure-based inference of protein function. PNAS.

[B33] Krishna SS, Grishin NV (2005). Structural drift: a possible path to protein fold change. Bioinformatics.

[B34] Domingues FS, Koppensteiner WA, Sippl MJ (2000). The role of protein structure in genomics. FEBS Letters.

[B35] Holm L, Sander C (1998). Touring protein fold space with Dali/FSSP. Nucleic Acids Research.

[B36] Efimov AV (1997). Structural trees for protein superfamilies. PROTEINS: Structure, Function and Genetics.

[B37] Marchler-Bauer A, Anderson JB, Cherukuri PF, DeWeese-Scott C, Ly G, Gwadz M, He S, Hurwitz DI, Jackson JD, Ke Z, Lanczycki CJ, Liebert CA, Liu C, Lu F, Marchler GH, Mullokandov M, Shoemaker BA, Simonyan V, JS JSS, Thiessen PA, Yamashita RA, Yin JJ, Zhang D, Bryant SH (2005). CDD: a Conserved Domain Database for protein classification. Nucleic Acids Research.

[B38] VAST INRA server. http://www-mig.jouy.inra.fr.

[B39] SHEBA server. http://lmbbi.nci.nih.gov.

[B40] MSCL server. http://abs.cit.nih.gov/strcomp.

[B41] Chandonia JM, Hon G, Walker NS, Conte LL, Koehl P, Levitt M, Brenner SE (2004). The ASTRAL compendium in 2004. Nucleic Acids Research.

[B42] Biowulf cluster. http://biowulf.nih.gov.

[B43] DeLano WL The PyMOL Molecular Graphics System. (2002) DeLano Scientific, San Carlos, CA, USA.

