# Supplementary material for: ROC and confusion analysis of structure comparison methods identify the main causes of divergence from manual protein classification
Source: BMC Bioinformatics. 2006 Apr 13;7:206. doi: 10.1186/1471-2105-7-206 (PMC1513609; doi:10.1186/1471-2105-7-206)
Supplement: Additional File 1 — • VAST and SHEBA heat maps • Complete heat map of VAST and SHEBA, obtained for a Pcli cutoff value of 2.5 and a Zscore cutoff value of 2.7, respectively. The cutoffs correspond to an overall average FPR of 0.01, and result in an overall average TPR of 0.616 and 0.748 for VAST and SHEBA respectively. The x (target folds) and y (query folds) axes of the heat maps are labeled by the SCOP folds, grouped into the different classes A, B, C, D, E, F and G. Each pixel within the heat maps represents a fold-specific true or false positive rate and takes value between 0 and 1. Diagonal and off-diagonal pixels correspond to fold-specific true positive rate TPRi(c) (eq. 4, see Methods) and fold-specific false positive rate FPRi,j(c) (eq. 3, see Methods) respectively. [file 1471-2105-7-206-S1.pdf]

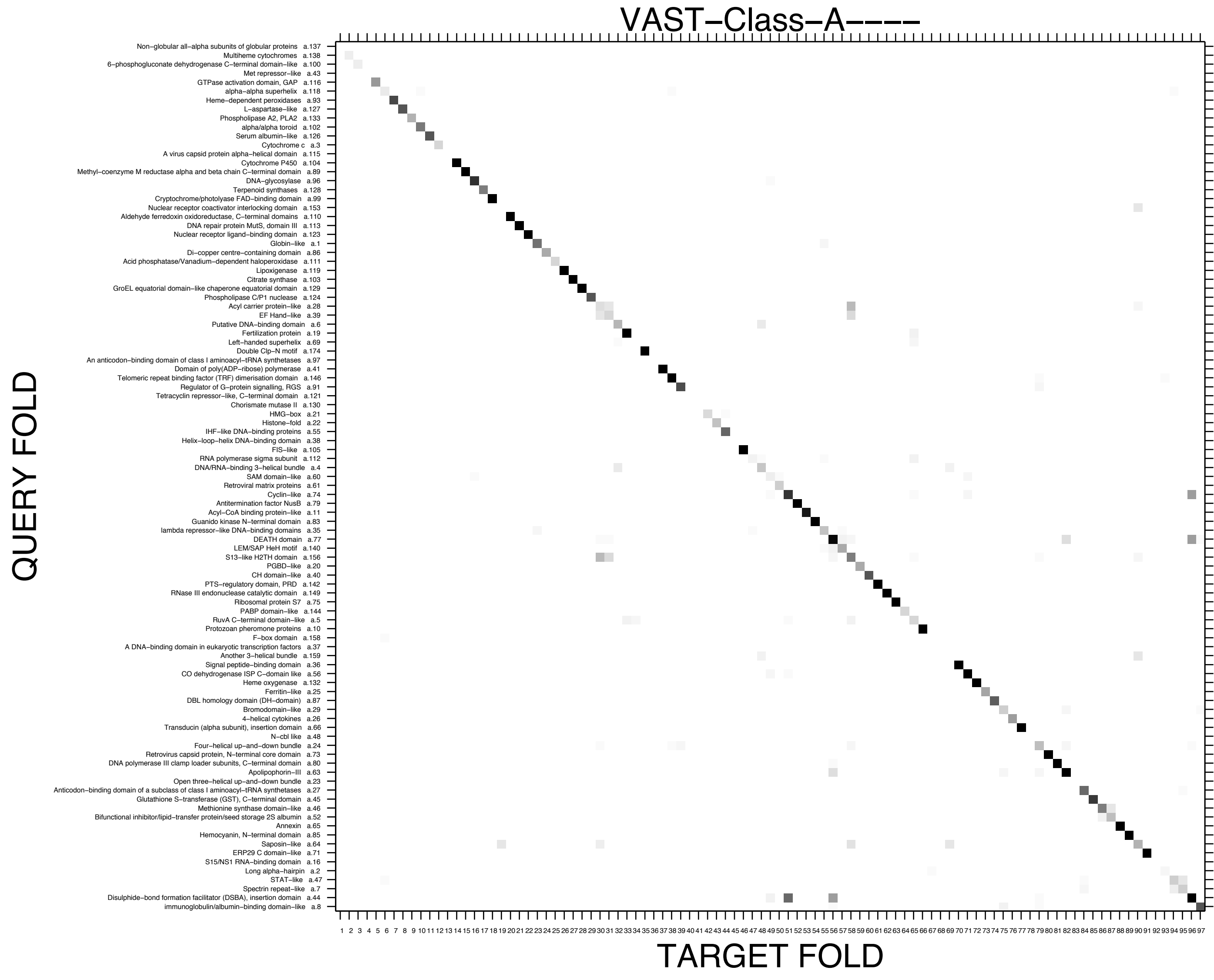
VAST−Class−A−−−−−
QUERY FOLD
Non−globular all−alpha subunits of globular proteins a.137
Multiheme cytochromes a.138
6−phosphogluconate dehydrogenase C−terminal domain−like a.100
Met repressor−like a.43
GTPase activation domain, GAP a.116
alpha−alpha superhelix a.118
Heme−dependent peroxidases a.93
L−aspartase−like a.127
Phospholipase A2, PLA2 a.133
alpha/alpha toroid a.102
Serum albumin−like a.126
Cytochrome c a.3
A virus capsid protein alpha−helical domain a.115
Cytochrome P450 a.104
Methyl−coenzyme M reductase alpha and beta chain C−terminal domain a.89
DNA−glycosylase a.96
Terpenoid synthases a.128
Cryptochrome/photolyase FAD−binding domain a.99
Nuclear receptor coactivator interlocking domain a.153
Aldehyde ferredoxin oxidoreductase, C−terminal domains a.110
DNA repair protein MutS, domain III a.113
Nuclear receptor ligand−binding domain a.123
Globin−like a.1
Di−copper centre−containing domain a.86
Acid phosphatase/Vanadium−dependent haloperoxidase a.111
Lipoxigenase a.119
Citrate synthase a.103
GroEL equatorial domain−like chaperone equatorial domain a.129
Phospholipase C/P1 nuclease a.124
Acyl carrier protein−like a.28
EF Hand−like a.39
Putative DNA−binding domain a.6
Fertilization protein a.19
Left−handed superhelix a.69
Double Clp−N motif a.174
An anticodon−binding domain of class I aminoacyl−tRNA synthetases a.97
Domain of poly(ADP−ribose) polymerase a.41
Telomeric repeat binding factor (TRF) dimerisation domain a.146
Regulator of G−protein signalling, RGS a.91
Tetracyclin repressor−like, C−terminal domain a.121
Chorismate mutase II a.130
HMG−box a.21
Histone−fold a.22
IHF−like DNA−binding proteins a.55
Helix−loop−helix DNA−binding domain a.38
FIS−like a.105
RNA polymerase sigma subunit a.112
DNA/RNA−binding 3−helical bundle a.4
SAM domain−like a.60
Retroviral matrix proteins a.61
Cyclin−like a.74
Antitermination factor NusB a.79
Acyl−CoA binding protein−like a.11
Guanido kinase N−terminal domain a.83
lambda repressor−like DNA−binding domains a.35
DEATH domain a.77
LEM/SAP HeH motif a.140
S13−like H2TH domain a.156
PGBD−like a.20
CH domain−like a.40
PTS−regulatory domain, PRD a.142
RNase III endonuclease catalytic domain a.149
Ribosomal protein S7 a.75
PABP domain−like a.144
RuvA C−terminal domain−like a.5
Protozoan pheromone proteins a.10
F−box domain a.158
A DNA−binding domain in eukaryotic transcription factors a.37
Another 3−helical bundle a.159
Signal peptide−binding domain a.36
CO dehydrogenase ISP C−domain like a.56
Heme oxygenase a.132
Ferritin−like a.25
DBL homology domain (DH−domain) a.87
Bromodomain−like a.29
4−helical cytokines a.26
Transducin (alpha subunit), insertion domain a.66
N−cbl like a.48
Four−helical up−and−down bundle a.24
Retrovirus capsid protein, N−terminal core domain a.73
DNA polymerase III clamp loader subunits, C−terminal domain a.80
Apolipophorin−III a.63
Open three−helical up−and−down bundle a.23
Anticodon−binding domain of a subclass of class I aminoacyl−tRNA synthetases a.27
Glutathione S−transferase (GST), C−terminal domain a.45
Methionine synthase domain−like a.46
Bifunctional inhibitor/lipid−transfer protein/seed storage 2S albumin a.52
Annexin a.65
Hemocyanin, N−terminal domain a.85
Saposin−like a.64
ERP29 C domain−like a.71
S15/NS1 RNA−binding domain a.16
Long alpha−hairpin a.2
STAT−like a.47
Spectrin repeat−like a.7
Disulphide−bond formation facilitator (DSBA), insertion domain a.44
immunoglobulin/albumin−binding domain−like a.8
1 2 3 4 5 6 7 8 9 10 11 12 13 14 15 16 17 18 19 20 21 22 23 24 25 26 27 28 29 30 31 32 33 34 35 36 37 38 39 40 41 42 43 44 45 46 47 48 49 50 51 52 53 54 55 56 57 58 59 60 61 62 63 64 65 66 67 68 69 70 71 72 73 74 75 76 77 78 79 80 81 82 83 84 85 86 87 88 89 90 91 92 93 94 95 96 97
TARGET FOLD

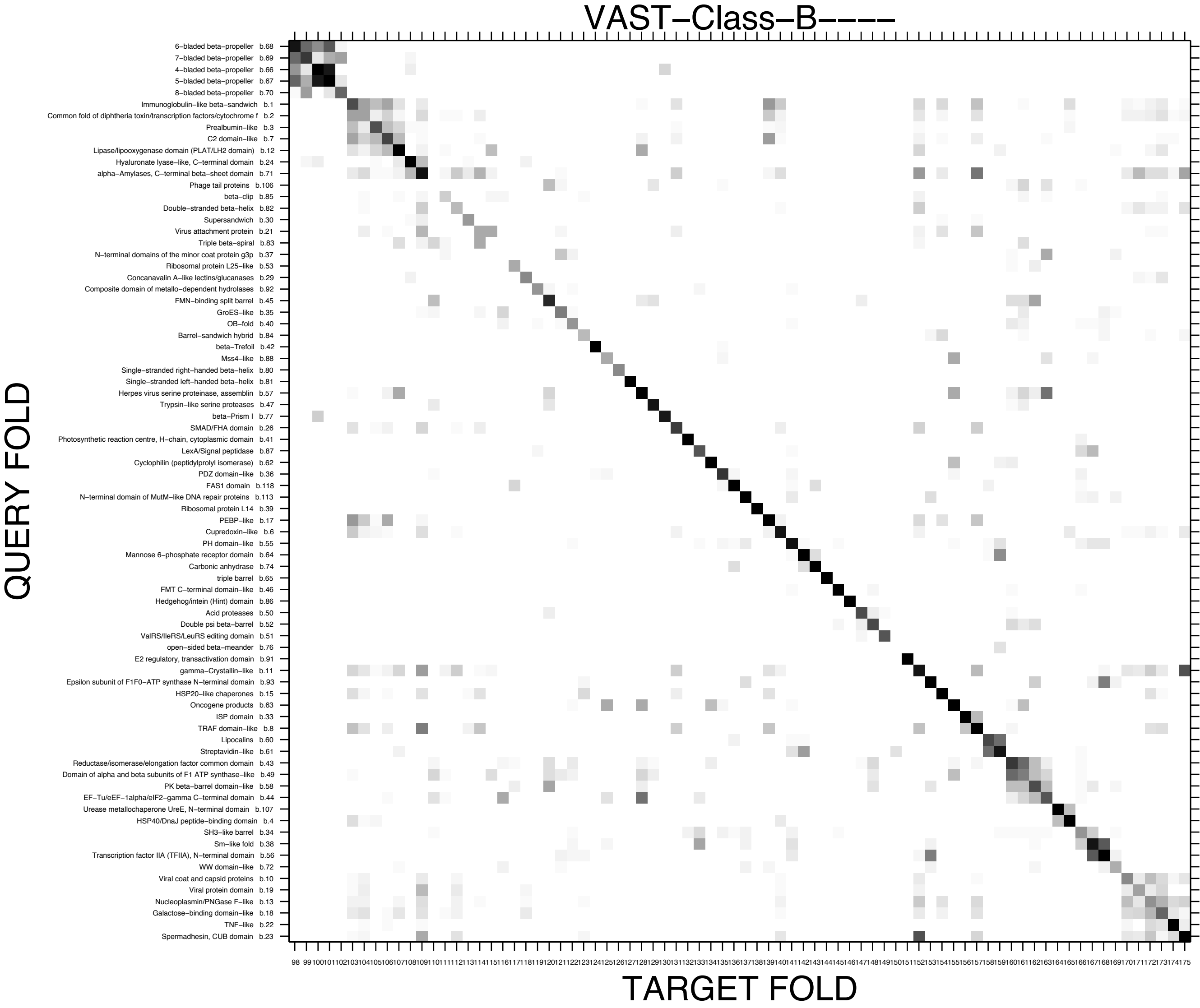

VAST−Class−B−−−−−
QUERY FOLD
TARGET FOLD
6−bladed beta−propeller b.68
7−bladed beta−propeller b.69
4−bladed beta−propeller b.66
5−bladed beta−propeller b.67
8−bladed beta−propeller b.70
Immunoglobulin−like beta−sandwich b.1
Common fold of diphtheria toxin/transcription factors/cytochrome f b.2
Prealbumin−like b.3
C2 domain−like b.7
Lipase/lipooxygenase domain (PLAT/LH2 domain) b.12
Hyaluronate lyase−like, C−terminal domain b.24
alpha−Amylases, C−terminal beta−sheet domain b.71
Phage tail proteins b.106
beta−clip b.85
Double−stranded beta−helix b.82
Supersandwich b.30
Virus attachment protein b.21
Triple beta−spiral b.83
N−terminal domains of the minor coat protein g3p b.37
Ribosomal protein L25−like b.53
Concanavalin A−like lectins/glucanases b.29
Composite domain of metallo−dependent hydrolases b.92
FMN−binding split barrel b.45
GroES−like b.35
OB−fold b.40
Barrel−sandwich hybrid b.84
beta−Trefoil b.42
Mss4−like b.88
Single−stranded right−handed beta−helix b.80
Single−stranded left−handed beta−helix b.81
Herpes virus serine proteinase, assemblin b.57
Trypsin−like serine proteases b.47
beta−Prism I b.77
SMAD/FHA domain b.26
Photosynthetic reaction centre, H−chain, cytoplasmic domain b.41
LexA/Signal peptidase b.87
Cyclophilin (peptidylprolyl isomerase) b.62
PDZ domain−like b.36
FAS1 domain b.118
N−terminal domain of MutM−like DNA repair proteins b.113
Ribosomal protein L14 b.39
PEBP−like b.17
Cupredoxin−like b.6
PH domain−like b.55
Mannose 6−phosphate receptor domain b.64
Carbonic anhydrase b.74
triple barrel b.65
FMT C−terminal domain−like b.46
Hedgehog/intein (Hint) domain b.86
Acid proteases b.50
Double psi beta−barrel b.52
ValRS/IleRS/LeuRS editing domain b.51
open−sided beta−meander b.76
E2 regulatory, transactivation domain b.91
gamma−Crystallin−like b.11
Epsilon subunit of F1F0−ATP synthase N−terminal domain b.93
HSP20−like chaperones b.15
Oncogene products b.63
ISP domain b.33
TRAF domain−like b.8
Lipocalins b.60
Streptavidin−like b.61
Reductase/isomerase/elongation factor common domain b.43
Domain of alpha and beta subunits of F1 ATP synthase−like b.49
PK beta−barrel domain−like b.58
EF−Tu/eEF−1alpha/eIF2−gamma C−terminal domain b.44
Urease metallochaperone UreE, N−terminal domain b.107
HSP40/DnaJ peptide−binding domain b.4
SH3−like barrel b.34
Sm−like fold b.38
Transcription factor IIA (TFIIA), N−terminal domain b.56
WW domain−like b.72
Viral coat and capsid proteins b.10
Viral protein domain b.19
Nucleoplasmin/PNGase F−like b.13
Galactose−binding domain−like b.18
TNF−like b.22
Spermadhesin, CUB domain b.23
98 99 100 101 102 103 104 105 106 107 108 109 110 111 112 113 114 115 116 117 118 119 120 121 122 123 124 125 126 127 128 129 130 131 132 133 134 135 136 137 138 139 140 141 142 143 144 145 146 147 148 149 150 151 152 153 154 155 156 157 158 159 160 161 162 163 164 165 166 167 168 169 170 171 172 173 174 175

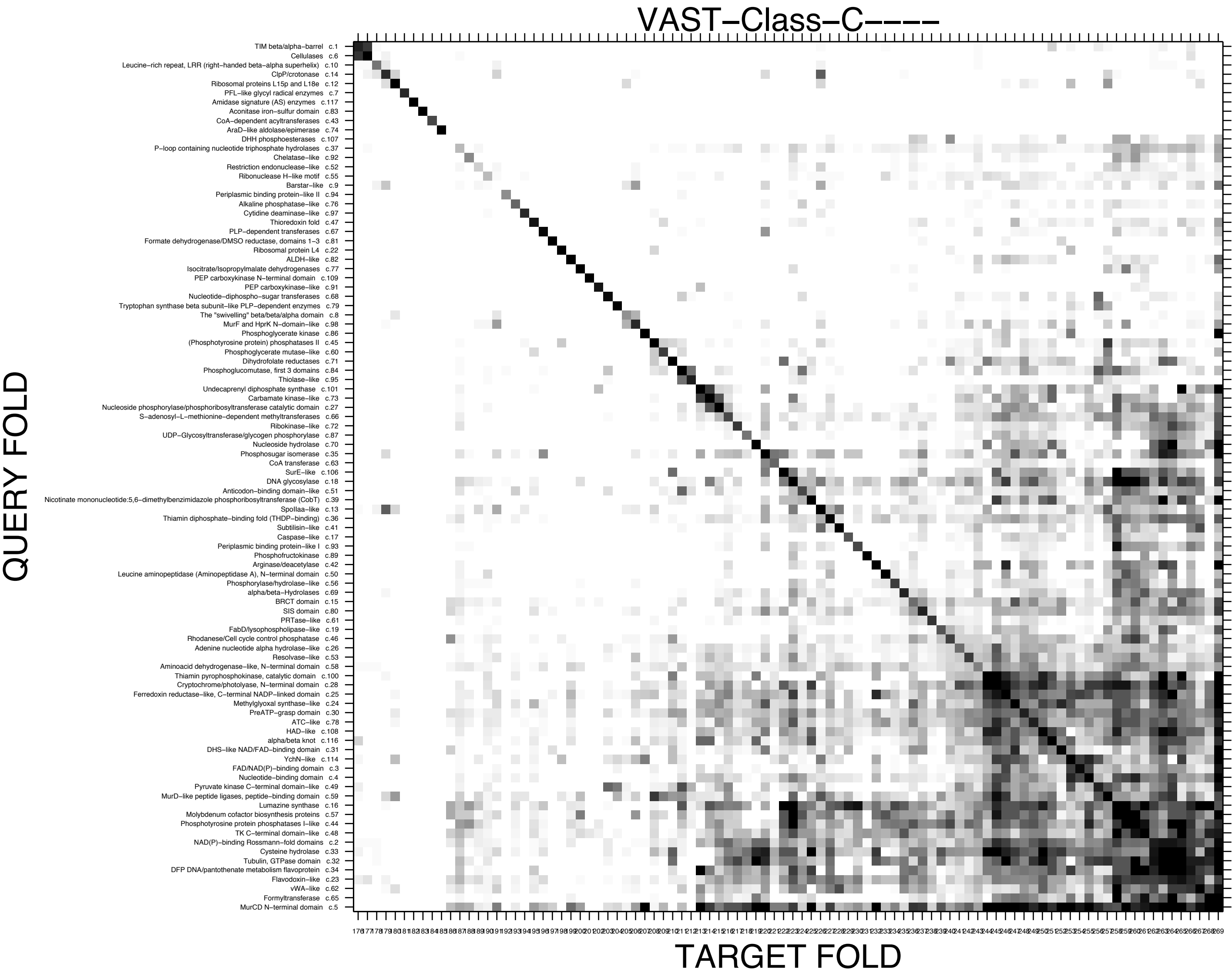
VAST–Class–C––––
QUERY FOLD
TARGET FOLD
TIM beta/alpha–barrel c.1
Cellulases c.6
Leucine–rich repeat, LRR (right–handed beta–alpha superhelix) c.10
ClpP/crotonase c.14
Ribosomal proteins L15p and L18e c.12
PFL–like glycyl radical enzymes c.7
Amidase signature (AS) enzymes c.117
Aconitase iron–sulfur domain c.83
CoA–dependent acyltransferases c.43
AraD–like aldolase/epimerase c.74
DHH phosphoesterases c.107
P–loop containing nucleotide triphosphate hydrolases c.37
Chelatase–like c.92
Restriction endonuclease–like c.52
Ribonuclease H–like motif c.55
Barstar–like c.9
Periplasmic binding protein–like II c.94
Alkaline phosphatase–like c.76
Cytidine deaminase–like c.97
Thioredoxin fold c.47
PLP–dependent transferases c.67
Formate dehydrogenase/DMSO reductase, domains 1–3 c.81
Ribosomal protein L4 c.22
ALDH–like c.82
Isocitrate/Isopropylmalate dehydrogenases c.77
PEP carboxykinase N–terminal domain c.109
PEP carboxykinase–like c.91
Nucleotide–diphospho–sugar transferases c.68
Tryptophan synthase beta subunit–like PLP–dependent enzymes c.79
The "swivelling" beta/beta/alpha domain c.8
MurF and HprK N–domain–like c.98
Phosphoglycerate kinase c.86
(Phosphotyrosine protein) phosphatases II c.45
Phosphoglycerate mutase–like c.60
Dihydrofolate reductases c.71
Phosphoglucomutase, first 3 domains c.84
Thiolase–like c.95
Undecaprenyl diphosphate synthase c.101
Carbamate kinase–like c.73
Nucleoside phosphorylase/phosphoribosyltransferase catalytic domain c.27
S–adenosyl–L–methionine–dependent methyltransferases c.66
Ribokinase–like c.72
UDP–Glycosyltransferase/glycogen phosphorylase c.87
Nucleoside hydrolase c.70
Phosphosugar isomerase c.35
CoA transferase c.63
SurE–like c.106
DNA glycosylase c.18
Anticodon–binding domain–like c.51
Nicotinate mononucleotide:5,6–dimethylbenzimidazole phosphoribosyltransferase (CobT) c.39
SpoIIaa–like c.13
Thiamin diphosphate–binding fold (THDP–binding) c.36
Subtilisin–like c.41
Caspase–like c.17
Periplasmic binding protein–like I c.93
Phosphofructokinase c.89
Arginase/deacetylase c.42
Leucine aminopeptidase (Aminopeptidase A), N–terminal domain c.50
Phosphorylase/hydrolase–like c.56
alpha/beta–Hydrolases c.69
BRCT domain c.15
SIS domain c.80
PRTase–like c.61
FabD/lysophospholipase–like c.19
Rhodanese/Cell cycle control phosphatase c.46
Adenine nucleotide alpha hydrolase–like c.26
Resolvase–like c.53
Aminoacid dehydrogenase–like, N–terminal domain c.58
Thiamin pyrophosphokinase, catalytic domain c.100
Cryptochrome/photolyase, N–terminal domain c.28
Ferredoxin reductase–like, C–terminal NADP–linked domain c.25
Methylglyoxal synthase–like c.24
PreATP–grasp domain c.30
ATC–like c.78
HAD–like c.108
alpha/beta knot c.116
DHS–like NAD/FAD–binding domain c.31
YchN–like c.114
FAD/NAD(P)–binding domain c.3
Nucleotide–binding domain c.4
Pyruvate kinase C–terminal domain–like c.49
MurD–like peptide ligases, peptide–binding domain c.59
Lumazine synthase c.16
Molybdenum cofactor biosynthesis proteins c.57
Phosphotyrosine protein phosphatases I–like c.44
TK C–terminal domain–like c.48
NAD(P)–binding Rossmann–fold domains c.2
Cysteine hydrolase c.33
Tubulin, GTPase domain c.32
DFP DNA/pantothenate metabolism flavoprotein c.34
Flavodoxin–like c.23
vWA–like c.62
Formyltransferase c.65
MurCD N–terminal domain c.5

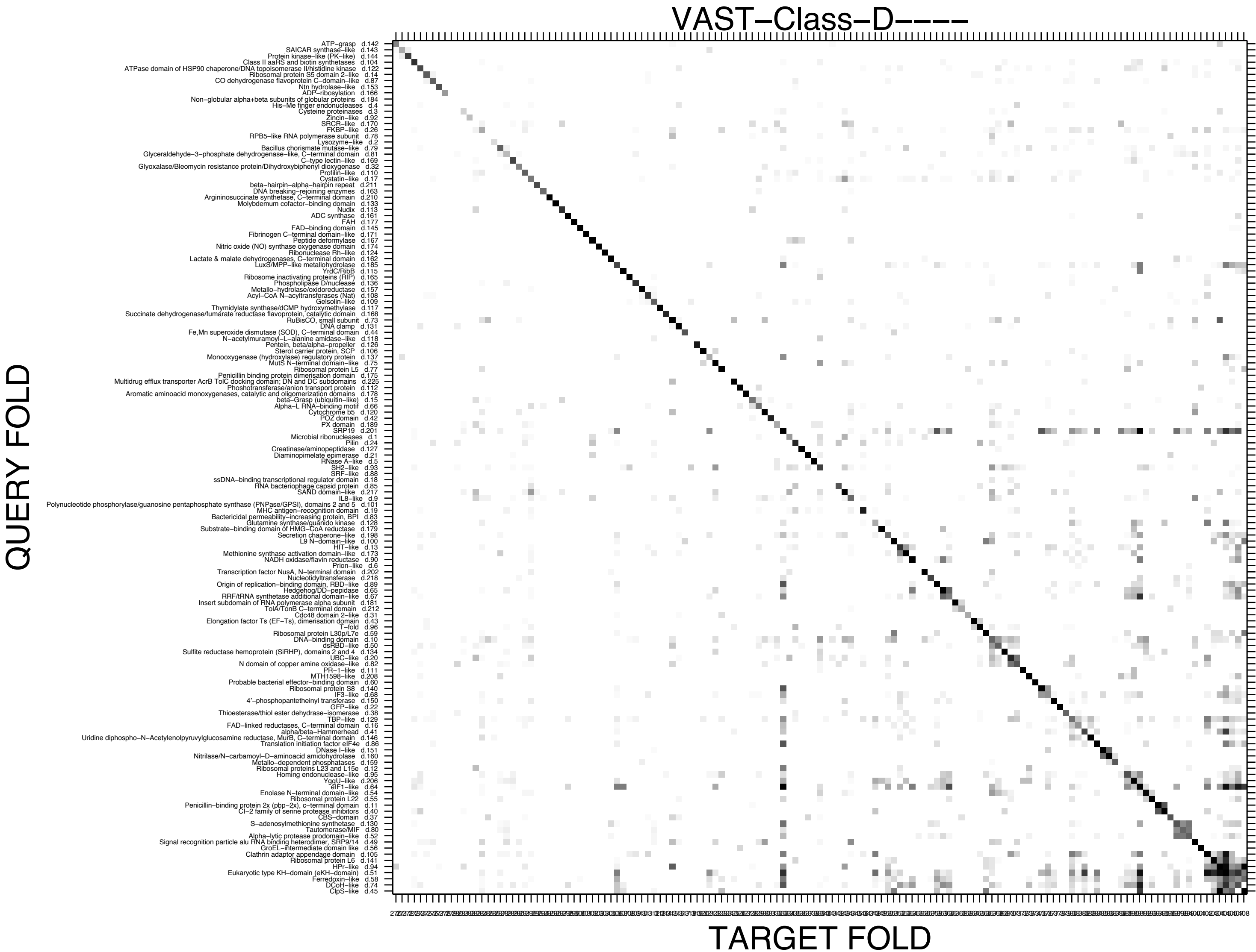

VAST–Class–D––––
QUERY FOLD
TARGET FOLD
ATP–grasp d.142
SAICAR synthase–like d.143
Protein kinase–like (PK–like) d.144
Class II aaRS and biotin synthetases d.104
ATPase domain of HSP90 chaperone/DNA topoisomerase II/histidine kinase d.122
Ribosomal protein S5 domain 2–like d.14
CO dehydrogenase flavoprotein C–domain–like d.87
Ntn hydrolase–like d.153
ADP–ribosylation d.166
Non–globular alpha+beta subunits of globular proteins d.184
His–Me finger endonucleases d.4
Cysteine proteinases d.3
Zincin–like d.92
SRCR–like d.170
FKBP–like d.26
RPB5–like RNA polymerase subunit d.78
Lysozyme–like d.2
Bacillus chorismate mutase–like d.79
Glyceraldehyde–3–phosphate dehydrogenase–like, C–terminal domain d.81
C–type lectin–like d.169
Glyoxalase/Bleomycin resistance protein/Dihydroxybiphenyl dioxygenase d.32
Profilin–like d.110
Cystatin–like d.17
beta–hairpin–alpha–hairpin repeat d.211
DNA breaking–rejoining enzymes d.163
Argininosuccinate synthetase, C–terminal domain d.210
Molybdemum cofactor–binding domain d.133
Nudix d.113
ADC synthase d.161
FAH d.177
FAD–binding domain d.145
Fibrinogen C–terminal domain–like d.171
Peptide deformylase d.167
Nitric oxide (NO) synthase oxygenase domain d.174
Ribonuclease Rh–like d.124
Lactate & malate dehydrogenases, C–terminal domain d.162
LuxS/MPP–like metallohydrolase d.185
YrdC/RibB d.115
Ribosome inactivating proteins (RIP) d.165
Phospholipase D/nuclease d.136
Metallo–hydrolase/oxidoreductase d.157
Acyl–CoA N–acyltransferases (Nat) d.108
Gelsolin–like d.109
Thymidylate synthase/dCMP hydroxymethylase d.117
Succinate dehydrogenase/fumarate reductase flavoprotein, catalytic domain d.168
RuBisCO, small subunit d.73
DNA clamp d.131
Fe,Mn superoxide dismutase (SOD), C–terminal domain d.44
N–acetylmuramoyl–L–alanine amidase–like d.118
Pentein, beta/alpha–propeller d.126
Sterol carrier protein, SCP d.106
Monooxygenase (hydroxylase) regulatory protein d.137
MutS N–terminal domain–like d.75
Ribosomal protein L5 d.77
Penicillin binding protein dimerisation domain d.175
Multidrug efflux transporter AcrB TolC docking domain; DN and DC subdomains d.225
Phoshotransferase/anion transport protein d.112
Aromatic aminoacid monoxygenases, catalytic and oligomerization domains d.178
beta–Grasp (ubiquitin–like) d.15
Alpha–L RNA–binding motif d.66
Cytochrome b5 d.120
POZ domain d.42
PX domain d.189
SRP19 d.201
Microbial ribonucleases d.1
Pilin d.24
Creatinase/aminopeptidase d.127
Diaminopimelate epimerase d.21
RNase A–like d.5
SH2–like d.93
SRF–like d.88
ssDNA–binding transcriptional regulator domain d.18
RNA bacteriophage capsid protein d.85
SAND domain–like d.217
IL8–like d.9
Polynucleotide phosphorylase/guanosine pentaphosphate synthase (PNPase/GPSI), domains 2 and 5 d.101
MHC antigen–recognition domain d.19
Bactericidal permeability–increasing protein, BPI d.83
Glutamine synthase/guanido kinase d.128
Substrate–binding domain of HMG–CoA reductase d.179
Secretion chaperone–like d.198
L9 N–domain–like d.100
HIT–like d.13
Methionine synthase activation domain–like d.173
NADH oxidase/flavin reductase d.90
Prion–like d.6
Transcription factor NusA, N–terminal domain d.202
Nucleotidyltransferase d.218
Origin of replication–binding domain, RBD–like d.89
Hedgehog/DD–pepidase d.65
RRF/tRNA synthetase additional domain–like d.67
Insert subdomain of RNA polymerase alpha subunit d.181
TolA/TonB C–terminal domain d.212
Cdc48 domain 2–like d.31
Elongation factor Ts (EF–Ts), dimerisation domain d.43
T–fold d.96
Ribosomal protein L30p/L7e d.59
DNA–binding domain d.10
dsRBD–like d.50
Sulfite reductase hemoprotein (SiRHP), domains 2 and 4 d.134
UBC–like d.20
N domain of copper amine oxidase–like d.82
PR–1–like d.111
MTH1598–like d.208
Probable bacterial effector–binding domain d.60
Ribosomal protein S8 d.140
IF3–like d.68
4'–phosphopantetheinyl transferase d.150
GFP–like d.22
Thioesterase/thiol ester dehydrase–isomerase d.38
TBP–like d.129
FAD–linked reductases, C–terminal domain d.16
alpha/beta–Hammerhead d.41
Uridine diphospho–N–Acetylenolpyruvylglucosamine reductase, MurB, C–terminal domain d.146
Translation initiation factor eIF4e d.86
DNase I–like d.151
Nitrilase/N–carbamoyl–D–aminoacid amidohydrolase d.160
Metallo–dependent phosphatases d.159
Ribosomal proteins L23 and L15e d.12
Homing endonuclease–like d.95
YggU–like d.206
eIF1–like d.64
Enolase N–terminal domain–like d.54
Ribosomal protein L22 d.55
Penicillin–binding protein 2x (pbp–2x), c–terminal domain d.11
CI–2 family of serine protease inhibitors d.40
CBS–domain d.37
S–adenosylmethionine synthetase d.130
Tautomerase/MIF d.80
Alpha–lytic protease prodomain–like d.52
Signal recognition particle alu RNA binding heterodimer, SRP9/14 d.49
GroEL–intermediate domain like d.56
Clathrin adaptor appendage domain d.105
Ribosomal protein L6 d.141
HPr–like d.94
Eukaryotic type KH–domain (eKH–domain) d.51
Ferredoxin–like d.58
DCoH–like d.74
ClpS–like d.45

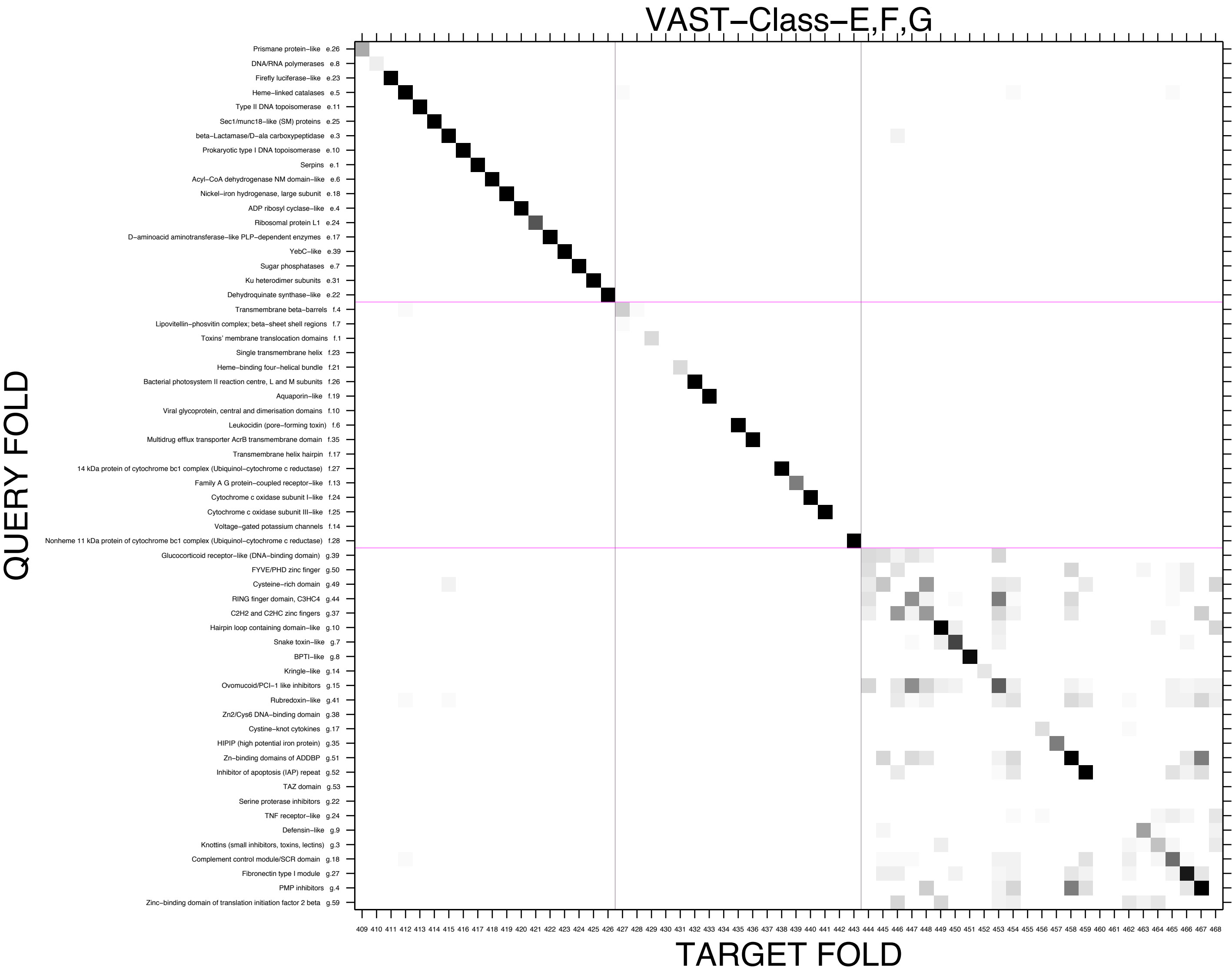

VAST-Class-E,F,G
QUERY FOLD
TARGET FOLD
Prismane protein-like e.26
DNA/RNA polymerases e.8
Firefly luciferase-like e.23
Heme-linked catalases e.5
Type II DNA topoisomerase e.11
Sec1/munc18-like (SM) proteins e.25
beta-Lactamase/D-ala carboxypeptidase e.3
Prokaryotic type I DNA topoisomerase e.10
Serpins e.1
Acyl-CoA dehydrogenase NM domain-like e.6
Nickel-iron hydrogenase, large subunit e.18
ADP ribosyl cyclase-like e.4
Ribosomal protein L1 e.24
D-aminoacid aminotransferase-like PLP-dependent enzymes e.17
YebC-like e.39
Sugar phosphatases e.7
Ku heterodimer subunits e.31
Dehydroquinate synthase-like e.22
Transmembrane beta-barrels f.4
Lipovitellin-phosvitin complex; beta-sheet shell regions f.7
Toxins' membrane translocation domains f.1
Single transmembrane helix f.23
Heme-binding four-helical bundle f.21
Bacterial photosystem II reaction centre, L and M subunits f.26
Aquaporin-like f.19
Viral glycoprotein, central and dimerisation domains f.10
Leukocidin (pore-forming toxin) f.6
Multidrug efflux transporter AcrB transmembrane domain f.35
Transmembrane helix hairpin f.17
14 kDa protein of cytochrome bc1 complex (Ubiquinol-cytochrome c reductase) f.27
Family A G protein-coupled receptor-like f.13
Cytochrome c oxidase subunit I-like f.24
Cytochrome c oxidase subunit III-like f.25
Voltage-gated potassium channels f.14
Nonheme 11 kDa protein of cytochrome bc1 complex (Ubiquinol-cytochrome c reductase) f.28
Glucocorticoid receptor-like (DNA-binding domain) g.39
FYVE/PHD zinc finger g.50
Cysteine-rich domain g.49
RING finger domain, C3HC4 g.44
C2H2 and C2HC zinc fingers g.37
Hairpin loop containing domain-like g.10
Snake toxin-like g.7
BPTI-like g.8
Kringle-like g.14
Ovomucoid/PCI-1 like inhibitors g.15
Rubredoxin-like g.41
Zn2/Cys6 DNA-binding domain g.38
Cystine-knot cytokines g.17
HIPIP (high potential iron protein) g.35
Zn-binding domains of ADDBP g.51
Inhibitor of apoptosis (IAP) repeat g.52
TAZ domain g.53
Serine protease inhibitors g.22
TNF receptor-like g.24
Defensin-like g.9
Knottins (small inhibitors, toxins, lectins) g.3
Complement control module/SCR domain g.18
Fibronectin type I module g.27
PMP inhibitors g.4
Zinc-binding domain of translation initiation factor 2 beta g.59
409 410 411 412 413 414 415 416 417 418 419 420 421 422 423 424 425 426 427 428 429 430 431 432 433 434 435 436 437 438 439 440 441 442 443 444 445 446 447 448 449 450 451 452 453 454 455 456 457 458 459 460 461 462 463 464 465 466 467 468

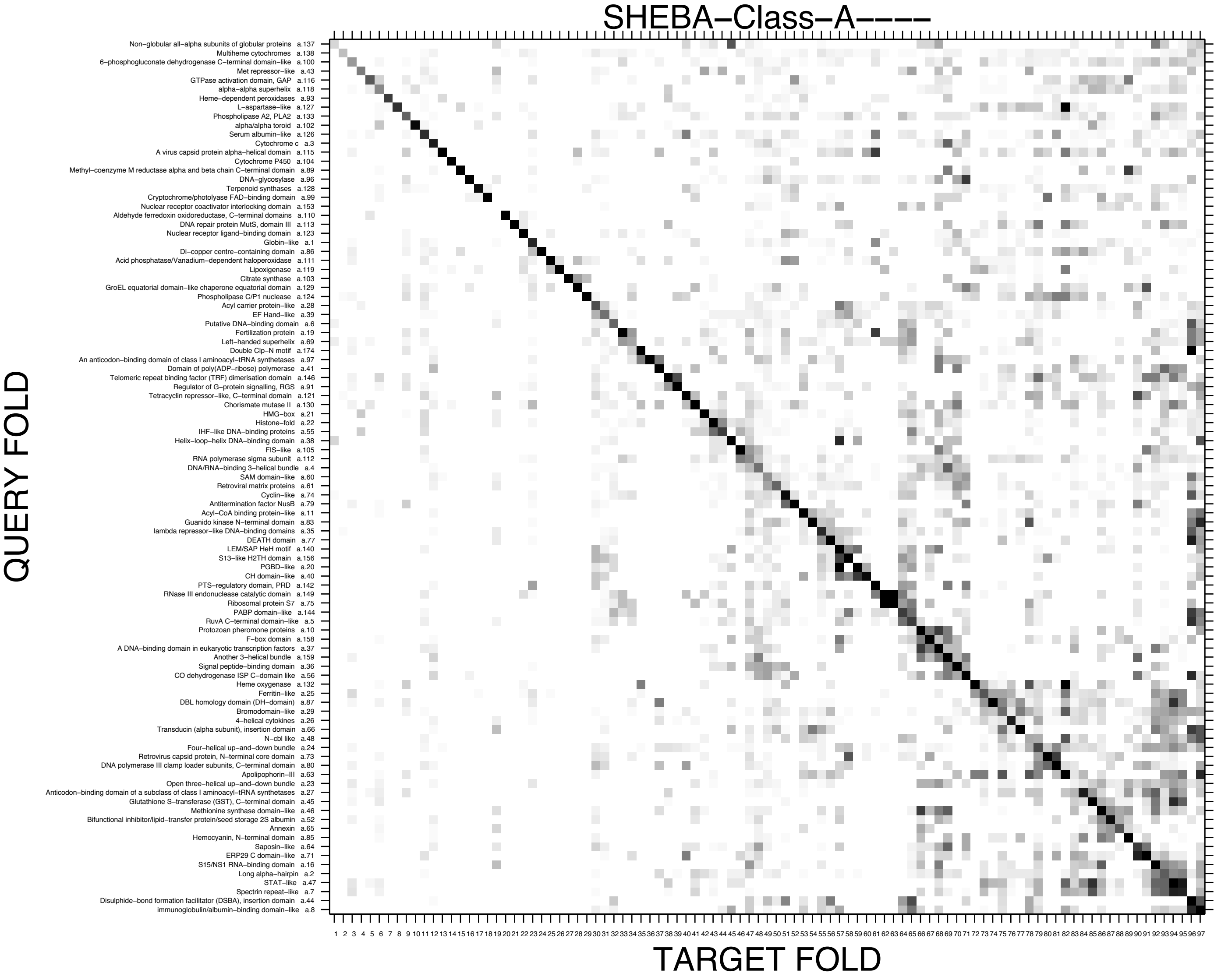
SHEBA−Class−A−−−−
QUERY FOLD
TARGET FOLD
Non−globular all−alpha subunits of globular proteins a.137
Multiheme cytochromes a.138
6−phosphogluconate dehydrogenase C−terminal domain−like a.100
Met repressor−like a.43
GTPase activation domain, GAP a.116
alpha−alpha superhelix a.118
Heme−dependent peroxidases a.93
L−aspartase−like a.127
Phospholipase A2, PLA2 a.133
alpha/alpha toroid a.102
Serum albumin−like a.126
Cytochrome c a.3
A virus capsid protein alpha−helical domain a.115
Cytochrome P450 a.104
Methyl−coenzyme M reductase alpha and beta chain C−terminal domain a.89
DNA−glycosylase a.96
Terpenoid synthases a.128
Cryptochrome/photolyase FAD−binding domain a.99
Nuclear receptor coactivator interlocking domain a.153
Aldehyde ferredoxin oxidoreductase, C−terminal domains a.110
DNA repair protein MutS, domain III a.113
Nuclear receptor ligand−binding domain a.123
Globin−like a.1
Di−copper centre−containing domain a.86
Acid phosphatase/Vanadium−dependent haloperoxidase a.111
Lipoxigenase a.119
Citrate synthase a.103
GroEL equatorial domain−like chaperone equatorial domain a.129
Phospholipase C/P1 nuclease a.124
Acyl carrier protein−like a.28
EF Hand−like a.39
Putative DNA−binding domain a.6
Fertilization protein a.19
Left−handed superhelix a.69
Double Clp−N motif a.174
An anticodon−binding domain of class I aminoacyl−tRNA synthetases a.97
Domain of poly(ADP−ribose) polymerase a.41
Telomeric repeat binding factor (TRF) dimerisation domain a.146
Regulator of G−protein signalling, RGS a.91
Tetracyclin repressor−like, C−terminal domain a.121
Chorismate mutase II a.130
HMG−box a.21
Histone−fold a.22
IHF−like DNA−binding proteins a.55
Helix−loop−helix DNA−binding domain a.38
FIS−like a.105
RNA polymerase sigma subunit a.112
DNA/RNA−binding 3−helical bundle a.4
SAM domain−like a.60
Retroviral matrix proteins a.61
Cyclin−like a.74
Antitermination factor NusB a.79
Acyl−CoA binding protein−like a.11
Guanido kinase N−terminal domain a.83
lambda repressor−like DNA−binding domains a.35
DEATH domain a.77
LEM/SAP HeH motif a.140
S13−like H2TH domain a.156
PGBD−like a.20
CH domain−like a.40
PTS−regulatory domain, PRD a.142
RNase III endonuclease catalytic domain a.149
Ribosomal protein S7 a.75
PABP domain−like a.144
RuvA C−terminal domain−like a.5
Protozoan pheromone proteins a.10
F−box domain a.158
A DNA−binding domain in eukaryotic transcription factors a.37
Another 3−helical bundle a.159
Signal peptide−binding domain a.36
CO dehydrogenase ISP C−domain like a.56
Heme oxygenase a.132
Ferritin−like a.25
DBL homology domain (DH−domain) a.87
Bromodomain−like a.29
4−helical cytokines a.26
Transducin (alpha subunit), insertion domain a.66
N−cbl like a.48
Four−helical up−and−down bundle a.24
Retrovirus capsid protein, N−terminal core domain a.73
DNA polymerase III clamp loader subunits, C−terminal domain a.80
Apolipophorin−III a.63
Open three−helical up−and−down bundle a.23
Anticodon−binding domain of a subclass of class I aminoacyl−tRNA synthetases a.27
Glutathione S−transferase (GST), C−terminal domain a.45
Methionine synthase domain−like a.46
Bifunctional inhibitor/lipid−transfer protein/seed storage 2S albumin a.52
Annexin a.65
Hemocyanin, N−terminal domain a.85
Saposin−like a.64
ERP29 C domain−like a.71
S15/NS1 RNA−binding domain a.16
Long alpha−hairpin a.2
STAT−like a.47
Spectrin repeat−like a.7
Disulphide−bond formation facilitator (DSBA), insertion domain a.44
immunoglobulin/albumin−binding domain−like a.8
1 2 3 4 5 6 7 8 9 10 11 12 13 14 15 16 17 18 19 20 21 22 23 24 25 26 27 28 29 30 31 32 33 34 35 36 37 38 39 40 41 42 43 44 45 46 47 48 49 50 51 52 53 54 55 56 57 58 59 60 61 62 63 64 65 66 67 68 69 70 71 72 73 74 75 76 77 78 79 80 81 82 83 84 85 86 87 88 89 90 91 92 93 94 95 96 97

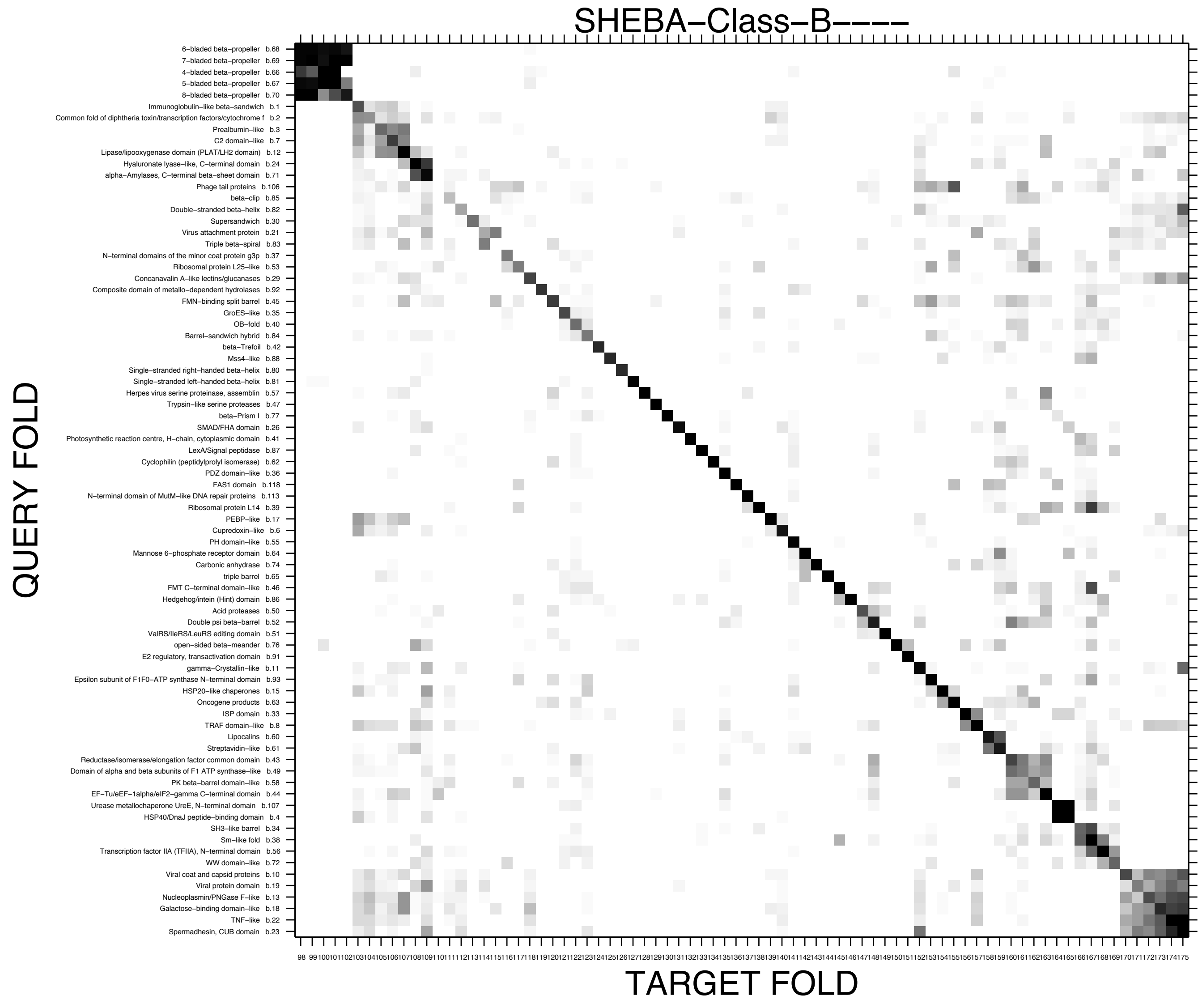
SHEBA–Class–B––––
QUERY FOLD
TARGET FOLD
6–bladed beta–propeller b.68
7–bladed beta–propeller b.69
4–bladed beta–propeller b.66
5–bladed beta–propeller b.67
8–bladed beta–propeller b.70
Immunoglobulin–like beta–sandwich b.1
Common fold of diphtheria toxin/transcription factors/cytochrome f b.2
Prealbumin–like b.3
C2 domain–like b.7
Lipase/lipooxygenase domain (PLAT/LH2 domain) b.12
Hyaluronate lyase–like, C–terminal domain b.24
alpha–Amylases, C–terminal beta–sheet domain b.71
Phage tail proteins b.106
beta–clip b.85
Double–stranded beta–helix b.82
Supersandwich b.30
Virus attachment protein b.21
Triple beta–spiral b.83
N–terminal domains of the minor coat protein g3p b.37
Ribosomal protein L25–like b.53
Concanavalin A–like lectins/glucanases b.29
Composite domain of metallo–dependent hydrolases b.92
FMN–binding split barrel b.45
GroES–like b.35
OB–fold b.40
Barrel–sandwich hybrid b.84
beta–Trefoil b.42
Mss4–like b.88
Single–stranded right–handed beta–helix b.80
Single–stranded left–handed beta–helix b.81
Herpes virus serine proteinase, assemblin b.57
Trypsin–like serine proteases b.47
beta–Prism I b.77
SMAD/FHA domain b.26
Photosynthetic reaction centre, H–chain, cytoplasmic domain b.41
LexA/Signal peptidase b.87
Cyclophilin (peptidylprolyl isomerase) b.62
PDZ domain–like b.36
FAS1 domain b.118
N–terminal domain of MutM–like DNA repair proteins b.113
Ribosomal protein L14 b.39
PEBP–like b.17
Cupredoxin–like b.6
PH domain–like b.55
Mannose 6–phosphate receptor domain b.64
Carbonic anhydrase b.74
triple barrel b.65
FMT C–terminal domain–like b.46
Hedgehog/intein (Hint) domain b.86
Acid proteases b.50
Double psi beta–barrel b.52
ValRS/IleRS/LeuRS editing domain b.51
open–sided beta–meander b.76
E2 regulatory, transactivation domain b.91
gamma–Crystallin–like b.11
Epsilon subunit of F1F0–ATP synthase N–terminal domain b.93
HSP20–like chaperones b.15
Oncogene products b.63
ISP domain b.33
TRAF domain–like b.8
Lipocalins b.60
Streptavidin–like b.61
Reductase/isomerase/elongation factor common domain b.43
Domain of alpha and beta subunits of F1 ATP synthase–like b.49
PK beta–barrel domain–like b.58
EF–Tu/eEF–1alpha/eIF2–gamma C–terminal domain b.44
Urease metallochaperone UreE, N–terminal domain b.107
HSP40/DnaJ peptide–binding domain b.4
SH3–like barrel b.34
Sm–like fold b.38
Transcription factor IIA (TFIIA), N–terminal domain b.56
WW domain–like b.72
Viral coat and capsid proteins b.10
Viral protein domain b.19
Nucleoplasmin/PNGase F–like b.13
Galactose–binding domain–like b.18
TNF–like b.22
Spermadhesin, CUB domain b.23

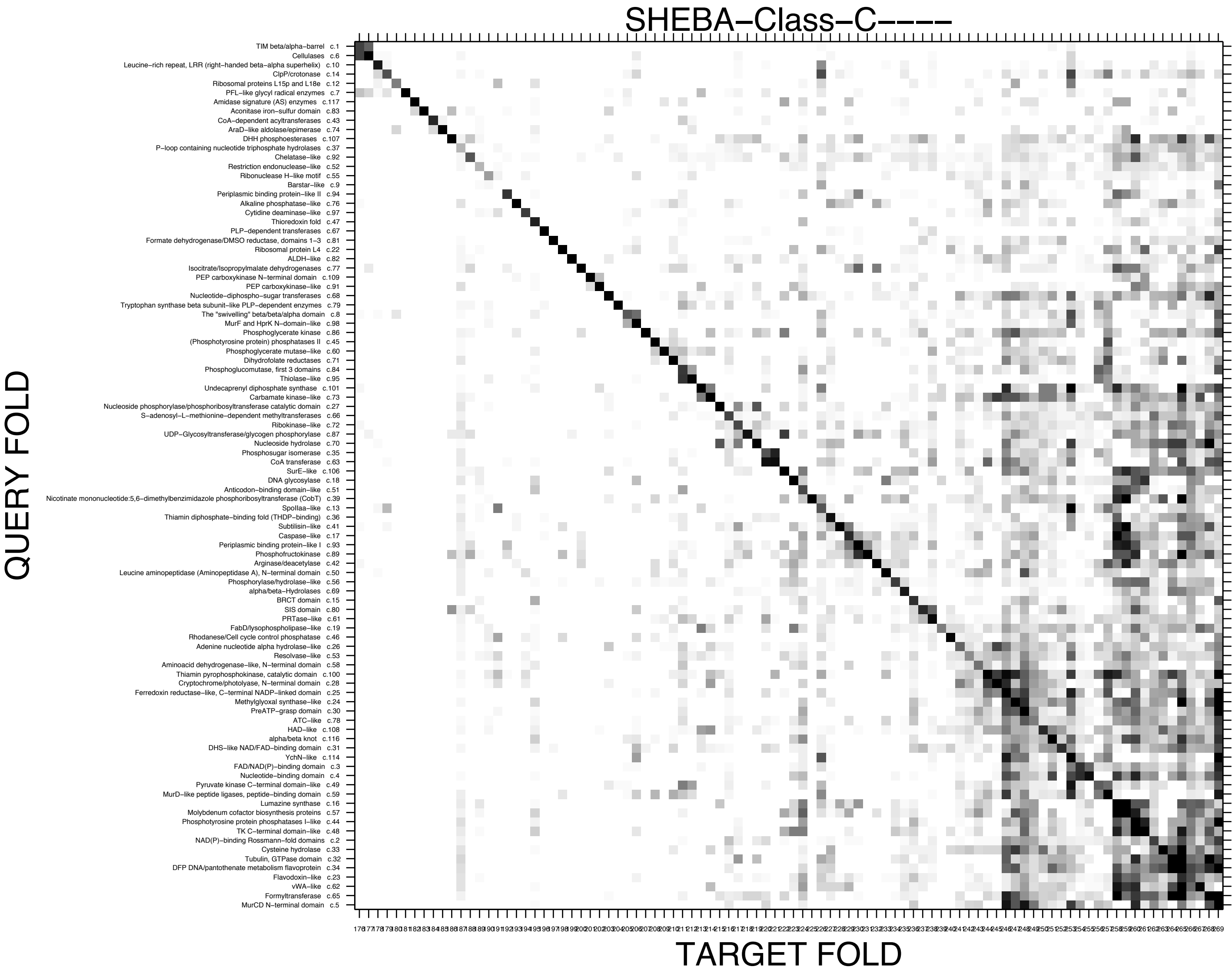

SHEBA−Class−C−−−−−
QUERY FOLD
TARGET FOLD
TIM beta/alpha−barrel c.1
Cellulases c.6
Leucine−rich repeat, LRR (right−handed beta−alpha superhelix) c.10
ClpP/crotonase c.14
Ribosomal proteins L15p and L18e c.12
PFL−like glycyl radical enzymes c.7
Amidase signature (AS) enzymes c.117
Aconitase iron−sulfur domain c.83
CoA−dependent acyltransferases c.43
AraD−like aldolase/epimerase c.74
DHH phosphoesterases c.107
P−loop containing nucleotide triphosphate hydrolases c.37
Chelatase−like c.92
Restriction endonuclease−like c.52
Ribonuclease H−like motif c.55
Barstar−like c.9
Periplasmic binding protein−like II c.94
Alkaline phosphatase−like c.76
Cytidine deaminase−like c.97
Thioredoxin fold c.47
PLP−dependent transferases c.67
Formate dehydrogenase/DMSO reductase, domains 1−3 c.81
Ribosomal protein L4 c.22
ALDH−like c.82
Isocitrate/Isopropylmalate dehydrogenases c.77
PEP carboxykinase N−terminal domain c.109
PEP carboxykinase−like c.91
Nucleotide−diphospho−sugar transferases c.68
Tryptophan synthase beta subunit−like PLP−dependent enzymes c.79
The "swivelling" beta/beta/alpha domain c.8
MurF and HprK N−domain−like c.98
Phosphoglycerate kinase c.86
(Phosphotyrosine protein) phosphatases II c.45
Phosphoglycerate mutase−like c.60
Dihydrofolate reductases c.71
Phosphoglucomutase, first 3 domains c.84
Thiolase−like c.95
Undecaprenyl diphosphate synthase c.101
Carbamate kinase−like c.73
Nucleoside phosphorylase/phosphoribosyltransferase catalytic domain c.27
S−adenosyl−L−methionine−dependent methyltransferases c.66
Ribokinase−like c.72
UDP−Glycosyltransferase/glycogen phosphorylase c.87
Nucleoside hydrolase c.70
Phosphosugar isomerase c.35
CoA transferase c.63
SurE−like c.106
DNA glycosylase c.18
Anticodon−binding domain−like c.51
Nicotinate mononucleotide:5,6−dimethylbenzimidazole phosphoribosyltransferase (CobT) c.39
SpoIIaa−like c.13
Thiamin diphosphate−binding fold (THDP−binding) c.36
Subtilisin−like c.41
Caspase−like c.17
Periplasmic binding protein−like I c.93
Phosphofructokinase c.89
Arginase/deacetylase c.42
Leucine aminopeptidase (Aminopeptidase A), N−terminal domain c.50
Phosphorylase/hydrolase−like c.56
alpha/beta−Hydrolases c.69
BRCT domain c.15
SIS domain c.80
PRTase−like c.61
FabD/lysophospholipase−like c.19
Rhodanese/Cell cycle control phosphatase c.46
Adenine nucleotide alpha hydrolase−like c.26
Resolvase−like c.53
Aminoacid dehydrogenase−like, N−terminal domain c.58
Thiamin pyrophosphokinase, catalytic domain c.100
Cryptochrome/photolyase, N−terminal domain c.28
Ferredoxin reductase−like, C−terminal NADP−linked domain c.25
Methylglyoxal synthase−like c.24
PreATP−grasp domain c.30
ATC−like c.78
HAD−like c.108
alpha/beta knot c.116
DHS−like NAD/FAD−binding domain c.31
YchN−like c.114
FAD/NAD(P)−binding domain c.3
Nucleotide−binding domain c.4
Pyruvate kinase C−terminal domain−like c.49
MurD−like peptide ligases, peptide−binding domain c.59
Lumazine synthase c.16
Molybdenum cofactor biosynthesis proteins c.57
Phosphotyrosine protein phosphatases I−like c.44
TK C−terminal domain−like c.48
NAD(P)−binding Rossmann−fold domains c.2
Cysteine hydrolase c.33
Tubulin, GTPase domain c.32
DFP DNA/pantothenate metabolism flavoprotein c.34
Flavodoxin−like c.23
vWA−like c.62
Formyltransferase c.65
MurCD N−terminal domain c.5

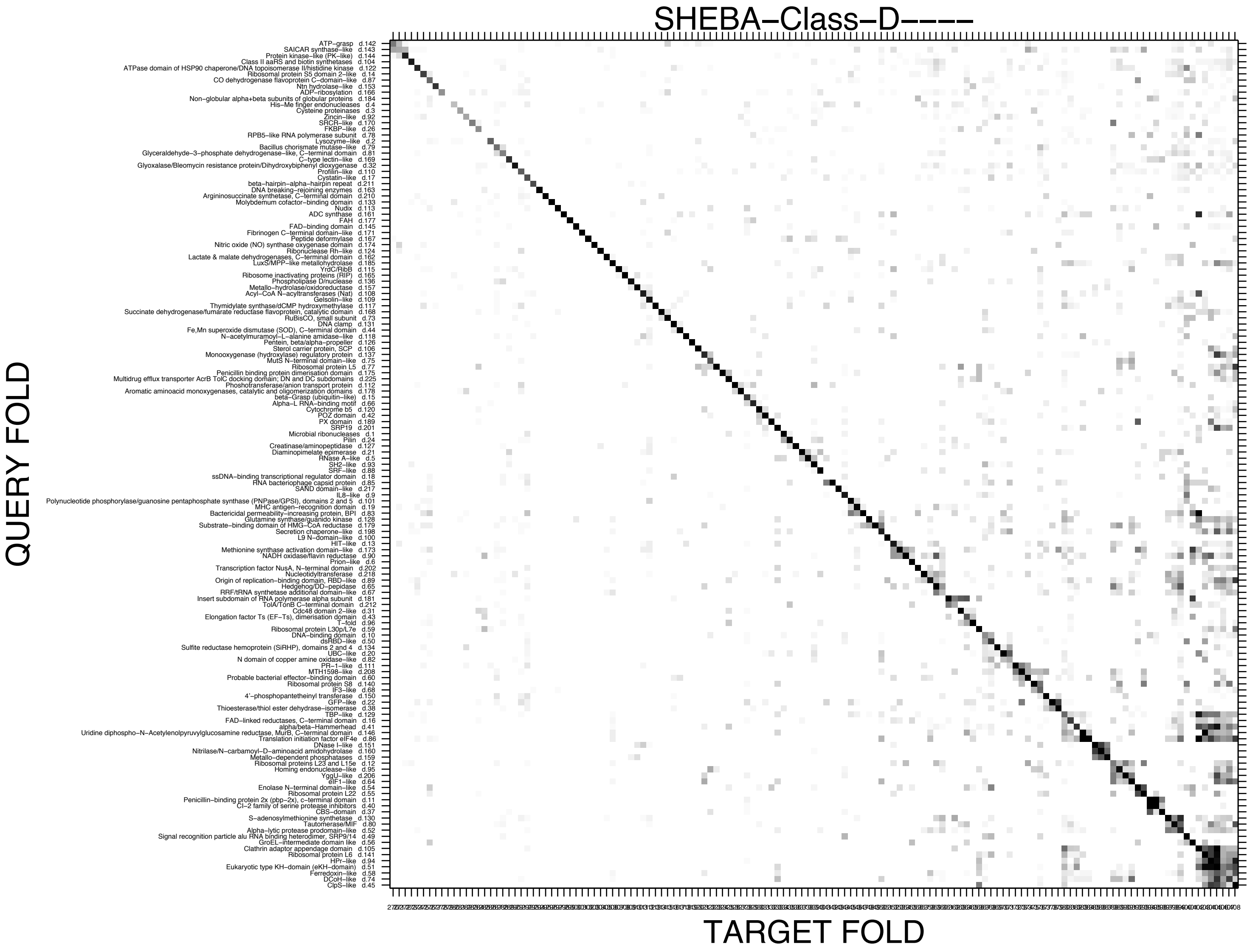
SHEBA−Class−D−−−−
QUERY FOLD
TARGET FOLD
ATP−grasp d.142
SAICAR synthase−like d.143
Protein kinase−like (PK−like) d.144
Class II aaRS and biotin synthetases d.104
ATPase domain of HSP90 chaperone/DNA topoisomerase II/histidine kinase d.122
Ribosomal protein S5 domain 2−like d.14
CO dehydrogenase flavoprotein C−domain−like d.87
Ntn hydrolase−like d.153
ADP−ribosylation d.166
Non−globular alpha+beta subunits of globular proteins d.184
His−Me finger endonucleases d.4
Cysteine proteinases d.3
Zincin−like d.92
SRCR−like d.170
FKBP−like d.26
RPB5−like RNA polymerase subunit d.78
Lysozyme−like d.2
Bacillus chorismate mutase−like d.79
Glyceraldehyde−3−phosphate dehydrogenase−like, C−terminal domain d.81
C−type lectin−like d.169
Glyoxalase/Bleomycin resistance protein/Dihydroxybiphenyl dioxygenase d.32
Profilin−like d.110
Cystatin−like d.17
beta−hairpin−alpha−hairpin repeat d.211
DNA breaking−rejoining enzymes d.163
Argininosuccinate synthetase, C−terminal domain d.210
Molybdemum cofactor−binding domain d.133
Nudix d.113
ADC synthase d.161
FAH d.177
FAD−binding domain d.145
Fibrinogen C−terminal domain−like d.171
Peptide deformylase d.167
Nitric oxide (NO) synthase oxygenase domain d.174
Ribonuclease Rh−like d.124
Lactate & malate dehydrogenases, C−terminal domain d.162
LuxS/MPP−like metallohydrolase d.185
YrdC/RibB d.115
Ribosome inactivating proteins (RIP) d.165
Phospholipase D/nuclease d.136
Metallo−hydrolase/oxidoreductase d.157
Acyl−CoA N−acyltransferases (Nat) d.108
Gelsolin−like d.109
Thymidylate synthase/dCMP hydroxymethylase d.117
Succinate dehydrogenase/fumarate reductase flavoprotein, catalytic domain d.168
RuBisCO, small subunit d.73
DNA clamp d.131
Fe,Mn superoxide dismutase (SOD), C−terminal domain d.44
N−acetylmuramoyl−L−alanine amidase−like d.118
Pentein, beta/alpha−propeller d.126
Sterol carrier protein, SCP d.106
Monooxygenase (hydroxylase) regulatory protein d.137
MutS N−terminal domain−like d.75
Ribosomal protein L5 d.77
Penicillin binding protein dimerisation domain d.175
Multidrug efflux transporter AcrB TolC docking domain; DN and DC subdomains d.225
Phoshotransferase/anion transport protein d.112
Aromatic aminoacid monoxygenases, catalytic and oligomerization domains d.178
beta−Grasp (ubiquitin−like) d.15
Alpha−L RNA−binding motif d.66
Cytochrome b5 d.120
POZ domain d.42
PX domain d.189
SRP19 d.201
Microbial ribonucleases d.1
Pilin d.24
Creatinase/aminopeptidase d.127
Diaminopimelate epimerase d.21
RNase A−like d.5
SH2−like d.93
SRF−like d.88
ssDNA−binding transcriptional regulator domain d.18
RNA bacteriophage capsid protein d.85
SAND domain−like d.217
IL8−like d.9
Polynucleotide phosphorylase/guanosine pentaphosphate synthase (PNPase/GPSI), domains 2 and 5 d.101
MHC antigen−recognition domain d.19
Bactericidal permeability−increasing protein, BPI d.83
Glutamine synthase/guanido kinase d.128
Substrate−binding domain of HMG−CoA reductase d.179
Secretion chaperone−like d.198
L9 N−domain−like d.100
HIT−like d.13
Methionine synthase activation domain−like d.173
NADH oxidase/flavin reductase d.90
Prion−like d.6
Transcription factor NusA, N−terminal domain d.202
Nucleotidyltransferase d.218
Origin of replication−binding domain, RBD−like d.89
Hedgehog/DD−pepidase d.65
RRF/tRNA synthetase additional domain−like d.67
Insert subdomain of RNA polymerase alpha subunit d.181
TolA/TonB C−terminal domain d.212
Cdc48 domain 2−like d.31
Elongation factor Ts (EF−Ts), dimerisation domain d.43
T−fold d.96
Ribosomal protein L30p/L7e d.59
DNA−binding domain d.10
dsRBD−like d.50
Sulfite reductase hemoprotein (SiRHP), domains 2 and 4 d.134
UBC−like d.20
N domain of copper amine oxidase−like d.82
PR−1−like d.111
MTH1598−like d.208
Probable bacterial effector−binding domain d.60
Ribosomal protein S8 d.140
IF3−like d.68
4'−phosphopantetheinyl transferase d.150
GFP−like d.22
Thioesterase/thiol ester dehydrase−isomerase d.38
TBP−like d.129
FAD−linked reductases, C−terminal domain d.16
alpha/beta−Hammerhead d.41
Uridine diphospho−N−Acetylenolpyruvylglucosamine reductase, MurB, C−terminal domain d.146
Translation initiation factor eIF4e d.86
DNase I−like d.151
Nitrilase/N−carbamoyl−D−aminoacid amidohydrolase d.160
Metallo−dependent phosphatases d.159
Ribosomal proteins L23 and L15e d.12
Homing endonuclease−like d.95
YggU−like d.206
eIF1−like d.64
Enolase N−terminal domain−like d.54
Ribosomal protein L22 d.55
Penicillin−binding protein 2x (pbp−2x), c−terminal domain d.11
CI−2 family of serine protease inhibitors d.40
CBS−domain d.37
S−adenosylmethionine synthetase d.130
Tautomerase/MIF d.80
Alpha−lytic protease prodomain−like d.52
Signal recognition particle alu RNA binding heterodimer, SRP9/14 d.49
GroEL−intermediate domain like d.56
Clathrin adaptor appendage domain d.105
Ribosomal protein L6 d.141
HPr−like d.94
Eukaryotic type KH−domain (eKH−domain) d.51
Ferredoxin−like d.58
DCoH−like d.74
ClpS−like d.45

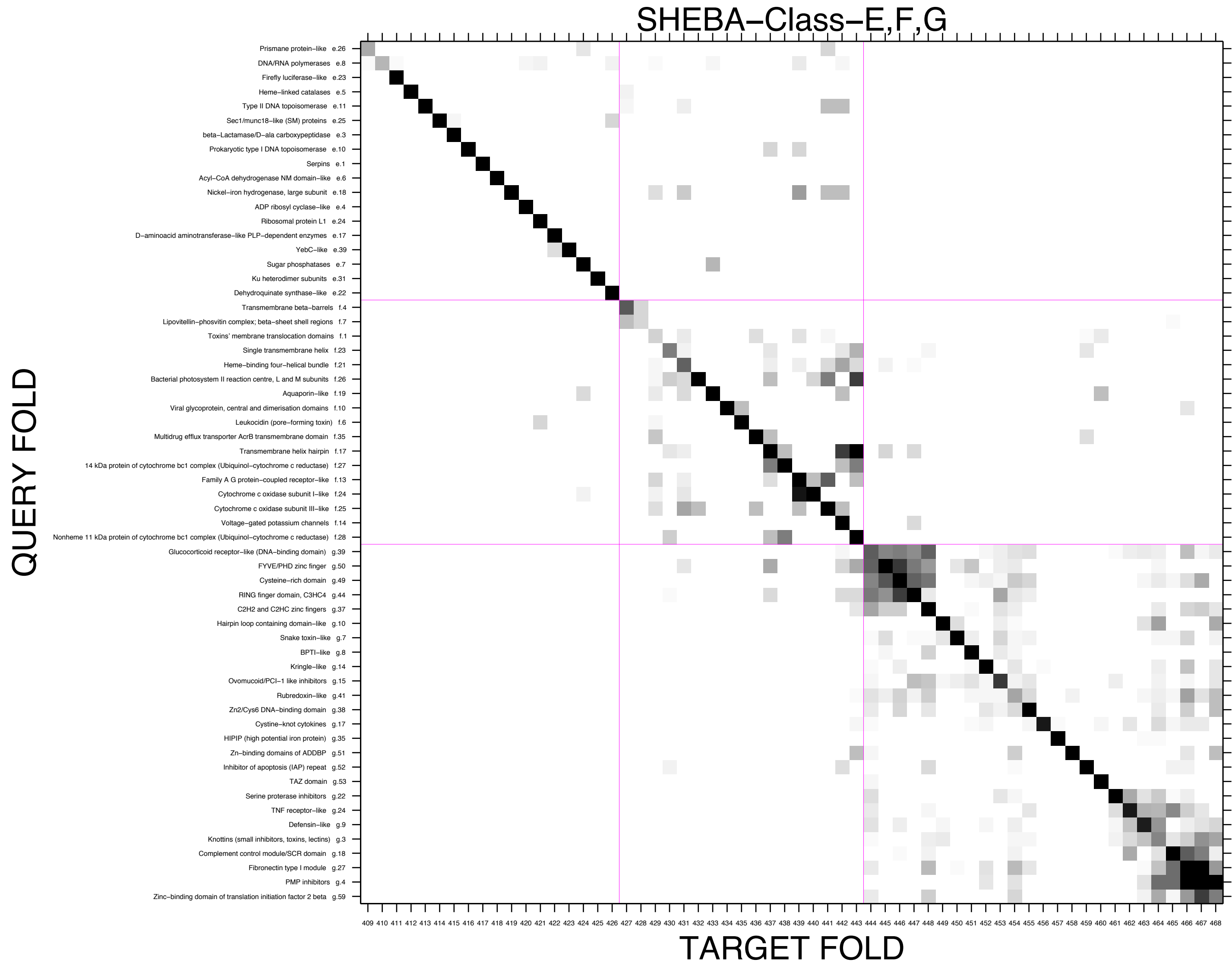

SHEBA−Class−E,F,G
QUERY FOLD
TARGET FOLD
Prismane protein−like e.26
DNA/RNA polymerases e.8
Firefly luciferase−like e.23
Heme−linked catalases e.5
Type II DNA topoisomerase e.11
Sec1/munc18−like (SM) proteins e.25
beta−Lactamase/D−ala carboxypeptidase e.3
Prokaryotic type I DNA topoisomerase e.10
Serpins e.1
Acyl−CoA dehydrogenase NM domain−like e.6
Nickel−iron hydrogenase, large subunit e.18
ADP ribosyl cyclase−like e.4
Ribosomal protein L1 e.24
D−aminoacid aminotransferase−like PLP−dependent enzymes e.17
YebC−like e.39
Sugar phosphatases e.7
Ku heterodimer subunits e.31
Dehydroquinate synthase−like e.22
Transmembrane beta−barrels f.4
Lipovitellin−phosvitin complex; beta−sheet shell regions f.7
Toxins' membrane translocation domains f.1
Single transmembrane helix f.23
Heme−binding four−helical bundle f.21
Bacterial photosystem II reaction centre, L and M subunits f.26
Aquaporin−like f.19
Viral glycoprotein, central and dimerisation domains f.10
Leukocidin (pore−forming toxin) f.6
Multidrug efflux transporter AcrB transmembrane domain f.35
Transmembrane helix hairpin f.17
14 kDa protein of cytochrome bc1 complex (Ubiquinol−cytochrome c reductase) f.27
Family A G protein−coupled receptor−like f.13
Cytochrome c oxidase subunit I−like f.24
Cytochrome c oxidase subunit III−like f.25
Voltage−gated potassium channels f.14
Nonheme 11 kDa protein of cytochrome bc1 complex (Ubiquinol−cytochrome c reductase) f.28
Glucocorticoid receptor−like (DNA−binding domain) g.39
FYVE/PHD zinc finger g.50
Cysteine−rich domain g.49
RING finger domain, C3HC4 g.44
C2H2 and C2HC zinc fingers g.37
Hairpin loop containing domain−like g.10
Snake toxin−like g.7
BPTI−like g.8
Kringle−like g.14
Ovomucoid/PCI−1 like inhibitors g.15
Rubredoxin−like g.41
Zn2/Cys6 DNA−binding domain g.38
Cystine−knot cytokines g.17
HIPIP (high potential iron protein) g.35
Zn−binding domains of ADDBP g.51
Inhibitor of apoptosis (IAP) repeat g.52
TAZ domain g.53
Serine protease inhibitors g.22
TNF receptor−like g.24
Defensin−like g.9
Knottins (small inhibitors, toxins, lectins) g.3
Complement control module/SCR domain g.18
Fibronectin type I module g.27
PMP inhibitors g.4
Zinc−binding domain of translation initiation factor 2 beta g.59
409 410 411 412 413 414 415 416 417 418 419 420 421 422 423 424 425 426 427 428 429 430 431 432 433 434 435 436 437 438 439 440 441 442 443 444 445 446 447 448 449 450 451 452 453 454 455 456 457 458 459 460 461 462 463 464 465 466 467 468
