# Supplementary material for: ROC and confusion analysis of structure comparison methods identify the main causes of divergence from manual protein classification
Source: BMC Bioinformatics. 2006 Apr 13;7:206. doi: 10.1186/1471-2105-7-206 (PMC1513609; doi:10.1186/1471-2105-7-206)
Supplement: Additional File 2 — • Fold-specific True Positive Rates (see Methods) at 1% False Positive Rate, for VAST and SHEBA, for 468 SCOP Folds in the order of the Heat Map. • Rows 1 to 7 correspond respectively to: the row number, the SCOP fold identifier, the number of domains within a fold, TPRi value obtained by the fold with VAST, TPRi value obtained by the fold with SHEBA, SCOP name of the fold, and SCOP description of the fold. [file 1471-2105-7-206-S2.pdf]

Table 3. True Positive Rates at 1% False Positive Rate, for VAST and SHEBA, for 468 SCOP Folds in the order of the Heat Map.

i	Class Fold	N_i	TPR VAST	TPR SHEBA	Fold Name	Fold Description
1	a.137	3	0.000	0.167	Non-globular all-alpha subunits of globular proteins	
2	a.138	17	0.077	0.257	Multiheme cytochromes	variable number of helices and little beta structure; not a true fold
3	a.100	10	0.078	0.400	6-phosphogluconate dehydrogenase C-terminal domain-like	multihelical; common core is formed around two long antiparallel helices related by (pseudo) twofold symmetry
4	a.43	2	0.000	0.500	Met repressor-like	core: 4 helices; array of 2 hairpins, opened
5	a.116	5	0.400	0.650	GTPase activation domain, GAP	multihelical
6	a.118	51	0.082	0.467	alpha-alpha superhelix	multihelical; 2 (curved) layers: alpha/alpha; right-handed superhelix
7	a.93	7	0.714	0.762	Heme-dependent peroxidases	multihelical; consists of two all-alpha domains
8	a.127	6	0.667	0.800	L-aspartase-like	multihelical, consists of three all-alpha domains
9	a.133	5	0.300	0.600	Phospholipase A2, PLA2	common core: 2 helices, disulphide-linked, and a calcium-binding loop
10	a.102	25	0.528	0.960	alpha/alpha toroid	multihelical; up to seven alpha-hairpins are arranged in closed circular array
11	a.126	6	0.667	0.800	Serum albumin-like	multihelical; one domain consists of two similar disulphide-linked subdomains
12	a.3	32	0.158	0.877	Cytochrome c	core: 3 helices; folded leaf, opened
13	a.115	2	0.000	1.000	A virus capsid protein alpha-helical domain	multihelical; three-helical bundle in the core is surrounded by non-conserved helices
14	a.104	12	1.000	1.000	Cytochrome P450	multihelical
15	a.89	2	1.000	1.000	Methyl-coenzyme M reductase alpha and beta chain C-terminal domain	multihelical bundle; contains buried central helix
16	a.96	6	0.800	1.000	DNA-glycosylase	multihelical; consists of two all-alpha domains
17	a.128	7	0.500	0.952	Terpenoid synthases	multihelical; core: 8 helices (C-J) are arranged in 2 parallel layers
18	a.99	3	1.000	1.000	Cryptochrome/photolyase FAD-binding domain	multihelical; consists of two all-alpha subdomains
19	a.153	2	0.000	0.000	Nuclear receptor coactivator interlocking domain	3 helices, non-globular array; forms interlocked heterodimers with its targets
20	a.110	2	1.000	1.000	Aldehyde ferredoxin oxidoreductase, C-terminal domains	multihelical; consists of two all-alpha domains
21	a.113	2	1.000	1.000	DNA repair protein MutS, domain III	multihelical; consists of 2 all-alpha subdomains
22	a.123	11	1.000	1.000	Nuclear receptor ligand-binding domain	multihelical; 3 layers or orthogonally packed helices
23	a.1	31	0.562	0.840	Globin-like	core: 6 helices; folded leaf, partly opened

Table 3. True Positive Rates at 1% False Positive Rate, for VAST and SHEBA, for 468 SCOP Folds in the order of the Heat Map.

i	Class Fold	N_i	TPR VAST	TPR SHEBA	Fold Name	Fold Description
24	a.86	4	0.333	1.000	Di-copper centre-containing domain	multihelical
25	a.111	4	0.167	1.000	Acid phosphatase/Vanadium-dependent haloperoxidase	multihelical; core: 5-helical bundle; binds cofactor at the beginning of third helix
26	a.119	2	1.000	1.000	Lipoxigenase	multihelical
27	a.103	4	1.000	1.000	Citrate synthase	multihelical; consists of two all-alpha domains
28	a.129	2	1.000	1.000	GroEL equatorial domain-like chaperone equatorial domain	multihelical; 8 helices arranged in 2 parallel layers
29	a.124	3	0.667	1.000	Phospholipase C/P1 nuclease	multihelical
30	a.28	9	0.139	0.694	Acyl carrier protein-like	4 helices, bundle; helix 3 is shorter than others; up-and-down
31	a.39	41	0.163	0.548	EF Hand-like	core: 4 helices; array of 2 hairpins, opened
32	a.6	7	0.286	0.595	Putative DNA-binding domain	core: 3 helices; architecture is similar to that of the "winged helix" fold but topology is different
33	a.19	2	1.000	1.000	Fertilization protein	core: 3 helices; bundle, closed, right-handed twist; up-and-down
34	a.69	5	0.000	0.350	Left-handed superhelix	core: 4-5 helices; bundle; left-handed superhelix
35	a.174	2	1.000	1.000	Double Clp-N motif	multihelical; array
36	a.97	2	0.000	1.000	An anticodon-binding domain of class I aminoacyl-tRNA synthetases	multihelical; consists of two all-alpha domains
37	a.41	2	1.000	1.000	Domain of poly(ADP-ribose) polymerase	core: 4 helices: bundle; unusual topology
38	a.146	2	1.000	1.000	Telomeric repeat binding factor (TRF) dimerisation domain	multihelical; can be divided into an alpha-alpha superhelix domain and a long alpha-haipin dimerisation domain
39	a.91	5	0.700	1.000	Regulator of G-protein signalling, RGS	multihelical; consists of two all-alpha subdomains
40	a.121	2	0.000	1.000	Tetracyclin repressor-like, C-terminal domain	multihelical; interlocked (homo)dimer
41	a.130	2	0.000	1.000	Chorismate mutase II	multihelical; core: 6 helices, bundle
42	a.21	7	0.143	1.000	HMG-box	3 helices; irregular array
43	a.22	14	0.242	1.000	Histone-fold	core: 3 helices; long middle helix is flanked at each end with shorter ones
44	a.55	5	0.600	0.750	IHF-like DNA-binding proteins	core: 4 helices; bundle, partly opened, capped with a beta-sheet
45	a.38	2	0.000	1.000	Helix-loop-helix DNA-binding domain	4-helices; bundle, closed, left-handed twist; 2 crossover connections
46	a.105	2	1.000	1.000	FIS-like	multihelical; intertwined dimer of identical 4-helical subunits
47	a.112	5	0.050	0.450	RNA polymerase sigma subunit	multihelical; consists of several all-alpha subdomains
48	a.4	117	0.219	0.560	DNA/RNA-binding 3-helical bundle	core: 3-helices; bundle, closed or partly opened, right-handed twist; up-and down
49	a.60	29	0.074	0.422	SAM domain-like	4-5 helices; bundle of two orthogonally packed alpha-hairpins; involved in the interactions with DNA and proteins

Table 3. True Positive Rates at 1% False Positive Rate, for VAST and SHEBA, for 468 SCOP Folds in the order of the Heat Map.

i	Class Fold	N_i	TPR VAST	TPR SHEBA	Fold Name	Fold Description
50	a.61	6	0.200	0.600	Retroviral matrix proteins	4-5 helices; right-handed superhelix
51	a.74	17	0.787	0.938	Cyclin-like	core: 5 helices; one helix is surrounded by the others
52	a.79	2	1.000	1.000	Antitermination factor NusB	6 helices: bundle; one central helix is surrounded by 5 others
53	a.11	5	0.900	1.000	Acyl-CoA binding protein-like	core: 3 helices; bundle, closed, left-handed twist; up-and-down
54	a.83	2	1.000	1.000	Guanido kinase N-terminal domain	irregular array of 6 short helices
55	a.35	11	0.245	0.836	lambda repressor-like DNA-binding domains	core: 4 helices; folded leaf, closed
56	a.77	12	0.962	0.992	DEATH domain	6 helices: closed bundle; greek-key; internal pseudo twofold symmetry
57	a.140	3	0.333	1.000	LEM/SAP HeH motif	helix-extended loop-helix; parallel helices
58	a.156	5	0.500	1.000	S13-like H2TH domain	core: 3-4 helices
59	a.20	3	0.333	1.000	PGBD-like	core: 3 helices; bundle, closed, left-handed twist; parallel
60	a.40	6	0.667	0.967	CH domain-like	core: 4 helices: bundle
61	a.142	2	1.000	1.000	PTS-regulatory domain, PRD	core: 4 helices; bundle, closed, right-handed twist; 1 crossover connection
62	a.149	2	1.000	1.000	RNase III endonuclease catalytic domain	core: 5 helices; one helix is surrounded by the others
63	a.75	2	1.000	1.000	Ribosomal protein S7	core: 5 helices; contains one more helix and a beta-hairpin outside the core
64	a.144	4	0.167	0.750	PABP domain-like	4 helices; an orthogonal array
65	a.5	10	0.167	0.756	RuvA C-terminal domain-like	3 helices; bundle, right-handed twist
66	a.10	2	1.000	1.000	Protozoan pheromone proteins	3 helices; bundle, closed, left-handed twist, up-and-down
67	a.158	2	0.000	1.000	F-box domain	multihelical; interlocked heterodimer with the Skp1 dimerisation domain
68	a.37	2	0.000	1.000	A DNA-binding domain in eukaryotic transcription factors	4 helices; the long C-terminal helix protrudes from the domain and binds to DNA
69	a.159	2	0.000	1.000	Another 3-helical bundle	topologically similar to the DNA/RNA-binding bundles; distinct packing
70	a.36	2	1.000	1.000	Signal peptide-binding domain	4 helices; orthogonal array
71	a.56	3	1.000	1.000	CO dehydrogenase ISP C-domain like	core: 4 helices, bundle
72	a.132	2	1.000	1.000	Heme oxygenase	multihelical; bundle
73	a.25	14	0.346	0.670	Ferritin-like	core: 4 helices; bundle, closed, left-handed twist; 1 crossover connection
74	a.87	6	0.633	1.000	DBL homology domain (DH-domain)	multihelical; core: 5-helical bundle
75	a.29	12	0.197	0.576	Bromodomain-like	4 helices; bundle; minor mirror variant of up-and-down topology

Table 3. True Positive Rates at 1% False Positive Rate, for VAST and SHEBA, for 468 SCOP Folds in the order of the Heat Map.

i	Class Fold	N_i	TPR VAST	TPR SHEBA	Fold Name	Fold Description
76	a.26	26	0.385	0.878	4-helical cytokines	core: 4 helices; bundle, closed; left-handed twist; 2 crossover connections
77	a.66	2	1.000	1.000	Transducin (alpha subunit), insertion domain	5 helices; folded leaf
78	a.48	3	0.000	0.333	N-cbl like	4 helices; bundle, left-handed twist; left-handed superhelix
79	a.24	31	0.253	0.703	Four-helical up-and-down bundle	core: 4 helices; bundle, closed or partly opened, left-handed twist; up-and-down
80	a.73	3	1.000	1.000	Retrovirus capsid protein, N-terminal core domain	core: 5 helices; bundle
81	a.80	4	1.000	1.000	DNA polymerase III clamp loader subunits, C-terminal domain	core: 5 helices: bundle
82	a.63	2	1.000	1.000	Apolipophorin-III	5 helices; bundle, closed, left-handed twist
83	a.23	5	0.000	0.050	Open three-helical up-and-down bundle	core: 3 helices; bundle, open
84	a.27	9	0.583	0.889	Anticodon-binding domain of a subclass of class I aminoacyl-tRNA synthetases	core: 4 helices; bundle; one loop crosses over one side of the bundle
85	a.45	20	0.779	1.000	Glutathione S-transferase (GST), C-terminal domain	core: 4 helices; bundle, closed, left-handed twist; right-handed superhelix
86	a.46	4	0.500	1.000	Methionine synthase domain-like	4 helices; bundle, left-handed twist; right-handed superhelix
87	a.52	5	0.250	0.950	Bifunctional inhibitor/lipid-transfer protein/seed storage 2S albumin	4 helices; folded leaf; right-handed superhelix
88	a.65	3	1.000	0.667	Annexin	5 helices; folded leaf, closed
89	a.85	2	1.000	1.000	Hemocyanin, N-terminal domain	6 helices; bundle; one central helix is surrounded by 5 others
90	a.64	5	0.300	0.750	Saposin-like	5 helices; folded leaf, closed
91	a.71	3	1.000	1.000	ERP29 C domain-like	5 helices; bundle
92	a.16	2	0.000	1.000	S15/NS1 RNA-binding domain	3 helices; irregular array
93	a.2	17	0.033	0.632	Long alpha-hairpin	2 helices; antiparallel hairpin, left-handed twist
94	a.47	5	0.200	1.000	STAT-like	4 long helices; bundle, left-handed twist (coiled colil); right-handed superhelix
95	a.7	19	0.202	0.854	Spectrin repeat-like	3 helices; bundle, closed, left-handed twist; up-and-down
96	a.44	2	1.000	1.000	Disulphide-bond formation facilitator (DSBA), insertion domain	4 helices; bundle, partly opened, left-handed twist; up-and-down
97	a.8	3	0.667	1.000	immunoglobulin/albumin-binding domain-like	3 helices; bundle, closed, left-handed twist; up-and-down; mirror topology to the spectrin-like fold

Table 3. True Positive Rates at 1% False Positive Rate, for VAST and SHEBA, for 468 SCOP Folds in the order of the Heat Map.

i	Class Fold	N_i	TPR VAST	TPR SHEBA	Fold Name	Fold Description
98	b.68	14	0.912	0.978	6-bladed beta-propeller	consists of six 4-stranded beta-sheet motifs; meander
99	b.69	19	0.772	0.988	7-bladed beta-propeller	consists of seven 4-stranded beta-sheet motifs; meander
100	b.66	5	1.000	1.000	4-bladed beta-propeller	consists of four 4-stranded beta-sheet motifs; meander
101	b.67	2	1.000	1.000	5-bladed beta-propeller	consists of five 4-stranded beta-sheet motifs; meander
102	b.70	5	0.600	0.900	8-bladed beta-propeller	consists of eight 4-stranded beta-sheet motifs; meander
103	b.1	241	0.701	0.680	Immunoglobulin-like beta-sandwich	sandwich; 7 strands in 2 sheets; greek-key
104	b.2	27	0.368	0.425	Common fold of diphtheria toxin/transcription factors/cytochrome f	sandwich; 9 strands in 2 sheet; greek-key; subclass of immunoglobin-like fold
105	b.3	11	0.673	0.582	Prealbumin-like	sandwich; 7 strands in 2 sheets, greek-key
106	b.7	14	0.725	0.758	C2 domain-like	sandwich; 8 strands in 2 sheets; greek-key
107	b.12	4	1.000	1.000	Lipase/lipooxygenase domain (PLAT/LH2 domain)	sandwich; 8 strands in 2 sheets; complex topology
108	b.24	3	1.000	1.000	Hyaluronate lyase-like, C-terminal domain	sandwich, 10 strands in 2 sheets; "folded meander"
109	b.71	21	0.924	0.964	alpha-Amylases, C-terminal beta-sheet domain	folded sheet; greek-key
110	b.106	3	0.000	0.000	Phage tail proteins	core: barrel; n=6, S=10; greek-key; topologically similar to the FMN-binding split barrel
111	b.85	13	0.192	0.250	beta-clip	double-stranded ribbon sharply bent in two places; the ribbon ends form incomplete barrel; jelly-roll
112	b.82	31	0.277	0.359	Double-stranded beta-helix	one turn of helix is made by two pairs of antiparallel strands linked with short turns
113	b.30	11	0.400	0.555	Supersandwich	sandwich; 18 strands in 2 sheets
114	b.21	3	0.333	0.333	Virus attachment protein	sandwich, 10 strands in 2 sheets; greek-key
115	b.83	2	0.000	0.000	Triple beta-spiral	trimer formed by the interlocking beta-hairpin repeat units
116	b.37	2	0.000	0.500	N-terminal domains of the minor coat protein g3p	core: barrel, in some members open; n*=4, S*=8; meander
117	b.53	3	0.333	0.500	Ribosomal protein L25-like	barrel, closed; n=6, S=10; complex topology
118	b.29	33	0.455	0.729	Concanavalin A-like lectins/glucanases	sandwich; 12-14 strands in 2 sheets; complex topology
119	b.92	8	0.411	0.786	Composite domain of metallo-dependent hydrolases	pseudobarrel; mixed sheet of 7 strand folded upon itself and "buckled" by two beta-turns
120	b.45	4	0.833	0.750	FMN-binding split barrel	barrel; n=6, S=10; greek-key
121	b.35	9	0.514	0.708	GroES-like	contains barrel, partly opened; n*=4, S*=8; meander
122	b.40	87	0.414	0.571	OB-fold	barrel, closed or partly opened n=5, S=10 or S=8; greek-key
123	b.84	14	0.275	0.522	Barrel-sandwich hybrid	sandwich of half-barrel shaped beta-sheets
124	b.42	24	0.998	0.835	beta-Trefoil	barrel, closed; n=6, S=12; and a hairpin triplet; meander

Table 3. True Positive Rates at 1% False Positive Rate, for VAST and SHEBA, for 468 SCOP Folds in the order of the Heat Map.

i	Class Fold	N_i	TPR VAST	TPR SHEBA	Fold Name	Fold Description
125	b.88	3	0.333	0.833	Mss4-like	complex fold made of several coiled beta-sheets
126	b.80	11	0.455	0.827	Single-stranded right-handed beta-helix	superhelix with each turn made by 3 strands with short links
127	b.81	9	1.000	0.972	Single-stranded left-handed beta-helix	superhelix with each turn made by 3 strands with short links
128	b.57	3	1.000	1.000	Herpes virus serine proteinase, assemblin	core: barrel, closed; n=7, S=8; complex topology
129	b.47	31	0.920	0.994	Trypsin-like serine proteases	barrel, closed; n=6, S=8; greek-key
130	b.77	5	0.900	1.000	beta-Prism I	consists of 3 4-stranded sheets; strands are parallel to the 3-fold axis
131	b.26	5	0.750	0.950	SMAD/FHA domain	sandwich; 11 strands in 2 sheets; greek-key
132	b.41	2	1.000	1.000	Photosynthetic reaction centre, H-chain, cytoplasmic domain	core: barrel, partly opened; n*=5, S*=8; meander
133	b.87	4	0.667	1.000	LexA/Signal peptidase	complex fold made of several coiled beta-sheets; contains an SH3-like barrel
134	b.62	2	1.000	1.000	Cyclophilin (peptidylprolyl isomerase)	barrel, closed; n=8, S=10; complex topology
135	b.36	15	0.790	1.000	PDZ domain-like	contains barrel, partly opened; n*=4, S*=8; meander; capped by alpha-helix
136	b.118	2	1.000	1.000	FAS1 domain	core: barrel, closed; n=7, S=12; meander
137	b.113	4	1.000	1.000	N-terminal domain of MutM-like DNA repair proteins	pseudobarrel; capped on both ends by alpha-helices
138	b.39	2	1.000	1.000	Ribosomal protein L14	barrel, closed; n=5, S=8, meander
139	b.17	3	1.000	1.000	PEBP-like	sandwich; 8 strands in 2 sheets; greek-key: partial topological similarity to immunoglobulin-like folds
140	b.6	38	0.908	0.922	Cupredoxin-like	sandwich; 7 strands in 2 sheets, greek-key
141	b.55	28	0.927	0.975	PH domain-like	barrel, partly opened; n*=6, S*=12; meander; capped by an alpha-helix
142	b.64	2	1.000	1.000	Mannose 6-phosphate receptor domain	barrel, partly open; n*=8, S*=10; one psi loop
143	b.74	4	1.000	1.000	Carbonic anhydrase	single sheet; 10 strands
144	b.65	2	1.000	1.000	triple barrel	dimer of two non-identical subunits; forms two similar barrels, n=8, S=10 each, that are fused together with the formation of third barrel, n=6, S=8
145	b.46	2	1.000	1.000	FMT C-terminal domain-like	barrel, open; n*=6, S*=10; greek-key
146	b.86	4	1.000	1.000	Hedgehog/intein (Hint) domain	complex fold made of five beta-hairpin units and a b-ribbon arc
147	b.50	12	0.697	0.682	Acid proteases	barrel, closed; n=6, S=10, complex topology
148	b.52	12	0.712	0.886	Double psi beta-barrel	barrel, closed; n=6, S=10; complex topology with crossover (psi) loops
149	b.51	4	0.667	1.000	ValRS/IleRS/LeuRS editing domain	core: barrel, closed; n=6, S=8; topology is similar to that of the acid proteases barrel

Table 3. True Positive Rates at 1% False Positive Rate, for VAST and SHEBA, for 468 SCOP Folds in the order of the Heat Map.

i	Class Fold	N_i	TPR VAST	TPR SHEBA	Fold Name	Fold Description
150	b.76	2	0.000	1.000	open-sided beta-meander	single sheet formed by beta-hairpin repeats; exposed on both sides in the middle
151	b.91	2	1.000	1.000	E2 regulatory, transactivation domain	complex fold made of bifurcated and coiled beta-sheets
152	b.11	11	0.909	0.955	gamma-Crystallin-like	sandwich; 8 strands in 2 sheets; greek-key
153	b.93	2	1.000	1.000	Epsilon subunit of F1F0-ATP synthase N-terminal domain	pseudobarrel: sandwich of two sheets packed at a positive interstrand angle and interconnected with many short turns
154	b.15	3	1.000	1.000	HSP20-like chaperones	sandwich; 8 strands in 2 sheets; greek-key
155	b.63	2	1.000	1.000	Oncogene products	barrel, closed; n=8, S=10; one overside connection
156	b.33	6	1.000	1.000	ISP domain	consists of two all-beta subdomains: conserved small domain has a rubredoxin-like fold; larger domain consists of 6 beta-stands packed in either sandwich of two 3-stranded sheets or closed barrel (n=6; S=8)
157	b.8	3	1.000	1.000	TRAF domain-like	sandwich; 8 strands in 2 sheets; greek-key
158	b.60	21	0.717	0.895	Lipocalins	barrel, closed or opened; n=8, S=12; meander
159	b.61	9	0.917	0.903	Streptavidin-like	barrel, closed; n=8, S=10; meander
160	b.43	27	0.769	0.724	Reductase/isomerase/elongatio n factor common domain	barrel, closed; n=6, S=10; greek-key
161	b.49	4	0.500	0.500	Domain of alpha and beta subunits of F1 ATP synthase-like	barrel, closed; n=6, S=8; greek-key
162	b.58	5	0.700	0.600	PK beta-barrel domain-like	barrel, closed; n=7, S=10; complex topology
163	b.44	3	0.667	1.000	EF-Tu/eEF-1alpha/eIF2-gamma C-terminal domain	barrel, closed; n=6, S=10; greek-key
164	b.107	2	1.000	1.000	Urease metallochaperone UreE, N-terminal domain	barrel, closed; n=6, S=8; a crossover loop topology
165	b.4	2	1.000	1.000	HSP40/DnaJ peptide-binding domain	sandwich; 6 strands in 2 sheets
166	b.34	52	0.407	0.604	SH3-like barrel	barrel, partly opened; n*=4, S*=8; meander
167	b.38	10	0.911	1.000	Sm-like fold	core: barrel, open; n*=4, S*=8; meander; SH3-like topology
168	b.56	2	1.000	1.000	Transcription factor IIA (TFIIA), N-terminal domain	barrel, closed; n=6, S=12; mixed beta-sheet
169	b.72	10	0.322	0.600	WW domain-like	core: 3-stranded meander beta-sheet
170	b.10	41	0.463	0.712	Viral coat and capsid proteins	sandwich; 8 strands in 2 sheets; jelly-roll
171	b.19	6	0.367	0.500	Viral protein domain	sandwich; 9 strands in 2 sheets; jelly-roll; form trimers
172	b.13	11	0.436	0.691	Nucleoplasmin/PNGase F-like	sandwich; 8 strands in 2 sheets; jelly-roll
173	b.18	24	0.601	0.832	Galactose-binding domain-like	sandwich; 9 strands in 2 sheets; jelly-roll
174	b.22	7	1.000	1.000	TNF-like	sandwich, 10 strands in 2 sheets; jelly-roll
175	b.23	2	1.000	1.000	Spermadhesin, CUB domain	sandwich, 10 strands in 2 sheets; jelly-roll

Table 3. True Positive Rates at 1% False Positive Rate, for VAST and SHEBA, for 468 SCOP Folds in the order of the Heat Map.

i	Class Fold	N_i	TPR VAST	TPR SHEBA	Fold Name	Fold Description
176	c.1	182	0.863	0.746	TIM beta/alpha-barrel	contains parallel beta-sheet barrel, closed; n=8, S=8; strand order 12345678
177	c.6	3	1.000	1.000	Cellulases	variant of beta/alpha barrel; parallel beta-sheet barrel, closed, n=7, S=8; strand order 1234567
178	c.10	18	0.546	0.892	Leucine-rich repeat, LRR (right-handed beta-alpha superhelix)	2 curved layers, a/b; parallel beta-sheet; order 1234...N
179	c.14	11	0.827	0.682	ClpP/crotonase	core: 4 turns of (beta-beta-alpha)n superhelix
180	c.12	2	1.000	0.500	Ribosomal proteins L15p and L18e	core: three turns of irregular (beta-beta-alpha)n superhelix
181	c.7	4	0.833	1.000	PFL-like glycyl radical enzymes	contains: barrel, closed; n=10, S=10; accommodates a hairpin loop inside the barrel
182	c.117	3	1.000	1.000	Amidase signature (AS) enzymes	possible duplication: the topologies of N- and C-terminal halves are similar; 3 layers: a/b/a; single mixed beta-sheet of 10 strands, order 213549A867 (A=10); strands from 5 to 9 are antiparallel to the rest
183	c.83	2	1.000	1.000	Aconitase iron-sulfur domain	consists of three similar domains with 3 layers (a/b/a) each; duplication
184	c.43	7	0.714	0.833	CoA-dependent acyltransferases	core: 2 layers, a/b; mixed beta-sheet of 6 strands, order 324561; strands 3 & 6 are antiparallel to the rest
185	c.74	3	1.000	1.000	AraD-like aldolase/epimerase	3 layers: a/b/a; mixed (mostly antiparallel) beta-sheet of 9 strands, order 432159876; left-handed crossover between strands 4 and 5
186	c.107	2	0.000	1.000	DHH phosphoesterases	consists of two non-similar domains
187	c.37	122	0.281	0.283	P-loop containing nucleotide triphosphate hydrolases	3 layers: a/b/a, parallel or mixed beta-sheets of variable sizes
188	c.92	11	0.455	0.664	Chelatase-like	duplication: tandem repeat of two domains; 3 layers (a/b/a); parallel beta-sheet of 4 strands, order 2134
189	c.52	23	0.202	0.294	Restriction endonuclease-like	core: 3 layers, a/b/a; mixed beta-sheet of 5 strands, order 12345; strands 2 &, in some families, 5 are antiparallel to the rest
190	c.55	53	0.279	0.379	Ribonuclease H-like motif	3 layers: a/b/a; mixed beta-sheet of 5 strands, order 32145; strand 2 is antiparallel to the rest
191	c.9	2	0.000	0.000	Barstar-like	2 layers, a/b; parallel beta-sheet of 3 strands, order 123
192	c.94	25	0.443	0.790	Periplasmic binding protein-like II	consists of two similar intertwined domain with 3 layers (a/b/a) each: duplication
193	c.76	6	0.600	1.000	Alkaline phosphatase-like	core:3 layers: a/b/a; mixed beta-sheet of 8 strands, order 43516728, strand 7 is antiparallel to the rest
194	c.97	4	0.833	0.750	Cytidine deaminase-like	core: alpha-beta(2)-(alpha-beta)2; 3 layers (a/b/a); mixed beta-sheet of 4 strands, order 2134; strand 2 is antiparallel to the rest
195	c.47	51	0.908	0.864	Thioredoxin fold	core: 3 layers, a/b/a; mixed beta-sheet of 4 strands, order 4312; strand 3 is antiparallel to the rest

Table 3. True Positive Rates at 1% False Positive Rate, for VAST and SHEBA, for 468 SCOP Folds in the order of the Heat Map.

i	Class Fold	N_i	TPR VAST	TPR SHEBA	Fold Name	Fold Description
196	c.67	35	0.966	1.000	PLP-dependent transferases	main domain: 3 layers: a/b/a, mixed beta-sheet of 7 strands, order 3245671; strand 7 is antiparallel to the rest
197	c.81	5	1.000	1.000	Formate dehydrogenase/DMSO reductase, domains 1-3	contains of two similar intertwined domains related by pseudodyad; duplication
198	c.22	2	1.000	1.000	Ribosomal protein L4	3 layers, a/b/a; core: parallel beta-sheet of 4 strands, order 1423
199	c.82	7	1.000	1.000	ALDH-like	consists of two similar domains with 3 layers (a/b/a) each; duplication
200	c.77	4	1.000	1.000	Isocitrate/Isopropylmalate dehydrogenases	consists of two intertwined (sub)domains related by pseudodyad; duplication
201	c.109	4	1.000	1.000	PEP carboxykinase N-terminal domain	contains mixed beta-sheets; topology is partly similar to that of the catalytic C-terminal domain
202	c.91	4	0.917	1.000	PEP carboxykinase-like	contains a P-loop NTP-binding motif; mixed beta-sheet folds into a barrel-like structure with helices packed on one side
203	c.68	15	0.948	0.981	Nucleotide-diphospho-sugar transferases	3 layers: a/b/a; mixed beta-sheet of 7 strands, order 3214657; strand 6 is antiparallel to the rest
204	c.79	6	1.000	1.000	Tryptophan synthase beta subunit-like PLP-dependent enzymes	consists of two similar domains related by pseudodyad; duplication
205	c.8	10	0.422	0.644	The "swivelling" beta/beta/alpha domain	3 layers: b/b/a; the central sheet is parallel, and the other one is antiparallel; there are some variations in topology
206	c.98	4	0.833	1.000	MurF and HprK N-domain-like	core: 3 layers, a/b/a; parallel beta-sheet of 4 strands, order 1234; structural similarity of the MurF and HprK extends beyond the core.
207	c.86	2	1.000	1.000	Phosphoglycerate kinase	consists of two non-similar domains, 3 layers (a/b/a) each
208	c.45	12	1.000	1.000	(Phosphotyrosine protein) phosphatases II	core: 3 layers, a/b/a; parallel beta-sheet of 4 strands, order 1432
209	c.60	7	0.762	0.976	Phosphoglycerate mutase-like	core: 3 layers, a/b/a; mixed beta-sheet of 6 strands, order 324156; strand 5 is antiparallel to the rest
210	c.71	8	1.000	1.000	Dihydrofolate reductases	3 layers: a/b/a; mixed beta-sheet of 8 strands, order 34251687; strand 8 is antiparallel to the rest
211	c.84	6	1.000	0.767	Phosphoglucomutase, first 3 domains	consists of three similar domains with 3 layers (a/b/a) each; duplication
212	c.95	13	0.885	0.981	Thiolase-like	consists of two similar domains related by pseudodyad; duplication
213	c.101	2	1.000	1.000	Undecaprenyl diphosphate synthase	3 layers: a/b/a; parallel beta-sheet of 6 strands, order 342156
214	c.73	2	1.000	1.000	Carbamate kinase-like	3 layers: a/b/a; mixed (mainly parallel) beta-sheet of 8 strands, order 34215786; strand 8 is antiparallel to the rest
215	c.27	3	1.000	1.000	Nucleoside phosphorylase/phosphoribosyltr ansferase catalytic domain	3 layers: a/b/a; parallel beta-sheet of 5 strands, order 32145; Rossmann-like

Table 3. True Positive Rates at 1% False Positive Rate, for VAST and SHEBA, for 468 SCOP Folds in the order of the Heat Map.

i	Class Fold	N_i	TPR VAST	TPR SHEBA	Fold Name	Fold Description
216	c.66	35	0.707	0.731	S-adenosyl-L-methionine-dependent methyltransferases	core: 3 layers, a/b/a; mixed beta-sheet of 7 strands, order 3214576; strand 7 is antiparallel to the rest
217	c.72	15	0.771	0.714	Ribokinase-like	core: 3 layers: a/b/a; mixed beta-sheet of 8 strands, order 21345678, strand 7 is antiparallel to the rest
218	c.87	6	0.533	0.933	UDP-Glycosyltransferase/glycogen phosphorylase	consists of two non-similar domains with 3 layers (a/b/a) each
219	c.70	2	1.000	1.000	Nucleoside hydrolase	core: 3 layers, a/b/a ; mixed beta-sheet of 8 strands, order 32145687; strand 7 is antiparallel to the rest
220	c.35	3	1.000	0.667	Phosphosugar isomerase	core: 3 layers: a/b/a; parallel beta-sheet of 6 strands, order 321456
221	c.63	5	0.600	0.950	CoA transferase	core: 3 layers: a/b/a; beta-sheet of 7 strands, order 4321567; part of sheet is folded upon itself and forms a barrel-like structure
222	c.106	2	1.000	1.000	SurE-like	3 layers: a/b/a; mixed beta-sheet of 9 strands, order 342156798; strands 3, 8 and 9 are antiparallel to the rest; left-handed crossover connection between strands 6 and 7
223	c.18	2	1.000	1.000	DNA glycosylase	3 layers, a/b/a; core: parallel beta-sheet of 4 strands, order 2134
224	c.51	13	0.718	0.718	Anticodon-binding domain-like	3 layers: a/b/a; mixed beta-sheet of five strands, order 21345; strand 4 is antiparallel to the rest
225	c.39	2	1.000	1.000	Nicotinate mononucleotide:5,6-dimethylbenzimidazole phosphoribosyltransferase (CobT)	3 layers: a/b/a, parallel beta-sheet of 7 strands, order 3214567
226	c.13	3	1.000	0.667	SpoIIaa-like	core: 4 turns of a (beta-alpha)n superhelix
227	c.36	19	0.982	0.933	Thiamin diphosphate-binding fold (THDP-binding)	3 layers: a/b/a; parallel beta-sheet of 6 strands, order 213465
228	c.41	4	1.000	1.000	Subtilisin-like	3 layers: a/b/a, parallel beta-sheet of 7 strands, order 2314567; left-handed crossover connection between strands 2 & 3
229	c.17	3	0.667	0.833	Caspase-like	3 layers, a/b/a; core: parallel beta-sheet of 4 strands, order 2134
230	c.93	13	0.718	1.000	Periplasmic binding protein-like I	consists of two similar intertwined domain with 3 layers (a/b/a) each: duplication
231	c.89	2	1.000	1.000	Phosphofructokinase	consists of two non-similar domains, 3 layers (a/b/a) each
232	c.42	3	1.000	1.000	Arginase/deacetylase	3 layers: a/b/a, parallel beta-sheet of 8 strands, order 21387456
233	c.50	2	1.000	1.000	Leucine aminopeptidase (Aminopeptidase A), N-terminal domain	3 layers: a/b/a; mixed beta-sheet of 5 strands, order 23145; strand 2 is antiparallel to the rest
234	c.56	24	0.757	0.761	Phosphorylase/hydrolase-like	core: 3 layers, a/b/a ; mixed beta-sheet of 5 strands: order 21354; strand 4 is antiparallel to the rest; contains crossover loops
235	c.69	51	0.847	0.892	alpha/beta-Hydrolases	core: 3 layers, a/b/a; mixed beta-sheet of 8 strands, order 12435678, strand 2 is antiparallel to the rest
236	c.15	7	0.571	0.810	BRCT domain	3 layers, a/b/a; core: parallel beta-sheet of 4 strands, order 2134

Table 3. True Positive Rates at 1% False Positive Rate, for VAST and SHEBA, for 468 SCOP Folds in the order of the Heat Map.

i	Class Fold	N_i	TPR VAST	TPR SHEBA	Fold Name	Fold Description
237	c.80	6	0.867	0.833	SIS domain	3 layers: a/b/a; parallel beta-sheet of 5 strands, order 21345
238	c.61	17	0.790	0.960	PRTase-like	core: 3 layers, a/b/a; mixed beta-sheet of 6 strands, order 321456; strand 3 is antiparallel to the rest
239	c.19	3	0.667	0.500	FabD/lysophospholipase-like	core: 3 layers, a/b/a; mixed beta-sheet of 6 strands, order 432156; strand 4 is antiparallel to the rest
240	c.46	7	0.738	0.976	Rhodanese/Cell cycle control phosphatase	3 layers: a/b/a; parallel beta-sheet of 5 strands, order 32451
241	c.26	42	0.563	0.595	Adenine nucleotide alpha hydrolase-like	core: 3 layers, a/b/a ; parallel beta-sheet of 5 strands, order 32145
242	c.53	9	0.750	0.444	Resolvase-like	Core: 3 layers: a/b/a; mixed beta-sheet of 5 strands, order 21345; strand 5 is antiparallel to the rest
243	c.58	11	0.491	0.555	Aminoacid dehydrogenase-like, N-terminal domain	core: 3 layers: a/b/a; parallel beta-sheet of 4 strands; 2134
244	c.100	2	1.000	1.000	Thiamin pyrophosphokinase, catalytic domain	3 layers: a/b/a; parallel beta-sheet of 6 strands, order 432156
245	c.28	4	1.000	1.000	Cryptochrome/photolyase, N-terminal domain	3 layers: a/b/a; parallel beta-sheet of 5 strands, order 32145; Rossmann-like
246	c.25	12	1.000	1.000	Ferredoxin reductase-like, C-terminal NADP-linked domain	3 layers, a/b/a; parallel beta-sheet of 5 strands, order 32145
247	c.24	3	1.000	1.000	Methylglyoxal synthase-like	3 layers, a/b/a; parallel beta-sheet of 5 strands, order 32145
248	c.30	11	0.873	0.991	PreATP-grasp domain	3 layers: a/b/a; parallel or mixed beta-sheet of 4 to 6 strands
249	c.78	12	0.871	0.515	ATC-like	consists of two similar domains related by pseudodyad; duplication
250	c.108	11	0.982	0.836	HAD-like	3 layers: a/b/a; parallel beta-sheet of 6 strands, order 321456
251	c.116	5	1.000	1.000	alpha/beta knot	core: 3 layers: a/b/a, parallel beta-sheet of 5 strands, order 21435; contains a deep trefoil knot
252	c.31	10	0.978	0.822	DHS-like NAD/FAD-binding domain	3 layers: a/b/a; parallel beta-sheet of 6 strands, order 321456; Rossmann-like
253	c.114	2	1.000	1.000	YchN-like	3 layers: a/b/a, core: parallel beta-sheet of 5 strands, order 43215
254	c.3	46	0.969	0.801	FAD/NAD(P)-binding domain	core: 3 layers, b/b/a; central parallel beta-sheet of 5 strands, order 32145; top antiparallel beta-sheet of 3 strands, meander
255	c.4	7	1.000	0.952	Nucleotide-binding domain	3 layers: a/b/a; parallel beta-sheet of 5 strands, order 32145; Rossmann-like
256	c.49	6	0.867	0.633	Pyruvate kinase C-terminal domain-like	3 layers: a/b/a; mixed beta-sheet of 5 strands, order 32145, strand 5 is antiparallel to the rest
257	c.59	5	1.000	1.000	MurD-like peptide ligases, peptide-binding domain	3 layers: a/b/a; mixed beta-sheet of 6 strands, order 126345; strand 1 is antiparallel to the rest
258	c.16	3	1.000	1.000	Lumazine synthase	3 layers, a/b/a; core: parallel beta-sheet of 4 strands, order 2134
259	c.57	3	1.000	1.000	Molybdenum cofactor biosynthesis proteins	3 layers: a/b/a; mixed beta-sheet of 5 strands; order 21354, strand 5 is antiparallel to the rest; permutation of the Phosphorylase/hydrolase-like fold

Table 3. True Positive Rates at 1% False Positive Rate, for VAST and SHEBA, for 468 SCOP Folds in the order of the Heat Map.

i	Class Fold	N_i	TPR VAST	TPR SHEBA	Fold Name	Fold Description
260	c.44	3	1.000	1.000	Phosphotyrosine protein phosphatases I-like	3 layers: a/b/a; parallel beta-sheet of 4 strands, order 2134
261	c.48	5	1.000	1.000	TK C-terminal domain-like	3 layers: a/b/a; mixed beta-sheet of 5 strands, order 13245, strand 1 is antiparallel to the rest
262	c.2	99	0.885	0.741	NAD(P)-binding Rossmann-fold domains	core: 3 layers, a/b/a; parallel beta-sheet of 6 strands, order 321456
263	c.33	3	1.000	1.000	Cysteine hydrolase	3 layers: a/b/a; parallel beta-sheet of 6 strands, order 321456
264	c.32	2	1.000	1.000	Tubulin, GTPase domain	3 layers: a/b/a; parallel beta-sheet of 6 strands, order 321456
265	c.34	2	1.000	1.000	DFP DNA/pantothenate metabolism flavoprotein	3 layers: a/b/a; parallel beta-sheet of 6 strands, order 321456
266	c.23	65	0.830	0.677	Flavodoxin-like	3 layers, a/b/a; parallel beta-sheet of 5 strand, order 21345
267	c.62	8	1.000	0.982	vWA-like	core: 3 layers, a/b/a; mixed beta-sheet of 6 strands, order 321456; strand 3 is antiparallel to the rest
268	c.65	3	1.000	1.000	Formyltransferase	3 layers: a/b/a; mixed beta-sheet of 7 strands, order 3214567; strand 6 is antiparallel to the rest
269	c.5	2	1.000	1.000	MurCD N-terminal domain	3 layers: a/b/a; parallel beta-sheet of 5 strands, order 32145; incomplete Rossmann-like fold; binds UDP group
270	d.142	20	0.482	0.516	ATP-grasp	Consists of two subdomains with different alpha+beta folds
271	d.143	3	0.333	0.333	SAICAR synthase-like	consists of two alpha+beta subdomains
272	d.144	26	0.751	0.886	Protein kinase-like (PK-like)	consists of two alpha+beta domains, C-terminal domain is mostly alpha helical
273	d.104	15	0.810	0.900	Class II aaRS and biotin synthetases	contains large mixed beta-sheet
274	d.122	11	0.709	0.845	ATPase domain of HSP90 chaperone/DNA topoisomerase II/histidine kinase	8 stranded mixed beta-sheet; 2 layers: alpha/beta
275	d.14	16	0.633	0.738	Ribosomal protein S5 domain 2-like	core: beta(3)-alpha-beta-alpha; 2 layers: alpha/beta; left-handed crossover
276	d.87	13	0.615	0.583	CO dehydrogenase flavoprotein C-domain-like	core: beta(3,4)-alpha(3); alpha+beta sandwich
277	d.153	25	0.687	0.788	Ntn hydrolase-like	4 layers: alpha/beta/beta/alpha; has an unusual sheet-to-sheet packing
278	d.166	10	0.400	0.422	ADP-ribosylation	unusual fold
279	d.184	2	0.000	0.000	Non-globular alpha+beta subunits of globular proteins	(
280	d.4	4	0.000	0.250	His-Me finger endonucleases	core: (alpha)-beta-omega_loop-beta-alpha; embeded in larger different structures
281	d.3	20	0.179	0.368	Cysteine proteinases	consists of one alpha-helix and 4 strands of antiparallel beta-sheet and contains the catalytic triad Cys-His-Asn
282	d.92	24	0.259	0.359	Zincin-like	contains mixed beta sheet with connection over free side of the sheet

Table 3. True Positive Rates at 1% False Positive Rate, for VAST and SHEBA, for 468 SCOP Folds in the order of the Heat Map.

i	Class Fold	N_i	TPR VAST	TPR SHEBA	Fold Name	Fold Description
283	d.170	2	0.000	0.500	SRCR-like	unusual fold; disulphide-rich; core: beta-x-alpha-beta-loop-beta
284	d.26	18	0.333	0.438	FKBP-like	core: beta(2)-alpha-beta(2); antiparallel beta-sheet
285	d.78	2	0.000	0.000	RPB5-like RNA polymerase subunit	core: beta-alpha-beta-alpha-beta(2); 2 layers, alpha/beta
286	d.2	12	0.152	0.614	Lysozyme-like	common alpha+beta motif for the active site region
287	d.79	15	0.624	0.552	Bacillus chorismate mutase-like	core: beta-alpha-beta-alpha-beta(2); mixed beta-sheet: order 1423, strand 4 is antiparallel to the rest
288	d.81	15	0.267	0.490	Glyceraldehyde-3-phosphate dehydrogenase-like, C-terminal domain	core: alpha-beta-alpha-beta(3); mixed sheet: 2134, strand 2 is parallel to strand 1
289	d.169	24	0.730	0.797	C-type lectin-like	unusual fold
290	d.32	14	0.473	0.907	Glyoxalase/Bleomycin resistance protein/Dihydroxybiphenyl dioxygenase	beta-alpha-beta(3); 2 layers: alpha/beta
291	d.110	18	0.611	0.637	Profilin-like	core: 2 alpha-helices and 5-stranded antiparallel sheet: order 21543; 3 layers: alpha/beta/alpha
292	d.17	24	0.522	0.685	Cystatin-like	Core: alpha-beta(4); helix packs against coiled antiparallel beta-sheet
293	d.211	12	0.697	0.697	beta-hairpin-alpha-hairpin repeat	multiple repeats of beta(2)-alpha(2) motif
294	d.163	7	0.571	0.976	DNA breaking-rejoining enzymes	core: alpha3-beta3-alpha4; one side of beta-sheet is exposed
295	d.210	2	1.000	1.000	Argininosuccinate synthetase, C-terminal domain	unusual fold
296	d.133	3	1.000	1.000	Molybdemum cofactor-binding domain	beta(2)-alpha-beta-alpha-beta; 2 layers: a/b; mixed sheet: order 1243: crossing loops
297	d.113	6	0.800	1.000	Nudix	beta(2)-alpha-beta(3)-alpha; 3 layers: alpha/beta/alpha; mixed sheet
298	d.161	3	1.000	1.000	ADC synthase	duplication: contains four repeats of alpha-beta(2)-beta motif arranged in a 4 layer core structure: alpha/beta/beta/alpha; orthogonally packed beta-sheets
299	d.177	3	1.000	1.000	FAH	unusual fold; contains 3 layers of beta-sheet structure
300	d.145	8	1.000	1.000	FAD-binding domain	consists of two alpha+beta subdomains
301	d.171	3	1.000	1.000	Fibrinogen C-terminal domain-like	unusual fold
302	d.167	3	1.000	1.000	Peptide deformylase	alpha-beta(5)-alpha; 3 layers: a/b/a; meander beta-sheet wraps around the C-terminal alpha-helix
303	d.174	2	1.000	1.000	Nitric oxide (NO) synthase oxygenase domain	unusual fold
304	d.124	6	1.000	1.000	Ribonuclease Rh-like	alpha+beta fold

Table 3. True Positive Rates at 1% False Positive Rate, for VAST and SHEBA, for 468 SCOP Folds in the order of the Heat Map.

i	Class Fold	N_i	TPR VAST	TPR SHEBA	Fold Name	Fold Description
305	d.162	11	1.000	1.000	Lactate & malate dehydrogenases, C-terminal domain	unusual fold, defines family
306	d.185	13	0.718	0.981	LuxS/MPP-like metallohydrolase	core: beta-alpha-beta(2)-alpha(2); 2 layers: alpha/beta
307	d.115	4	1.000	1.000	YrdC/RibB	core: alpha-beta(2)-alpha-beta-alpha(2)-beta(2)-alpha-beta-alpha-beta; 3 layers; mixed twisted sheet of 7 strands; order 7126354; strands 7 and 1 are parallel to each other
308	d.165	7	1.000	1.000	Ribosome inactivating proteins (RIP)	contains mixed beta-sheet
309	d.136	5	0.800	0.950	Phospholipase D/nuclease	beta-alpha-beta-alpha-beta-alpha-beta(4)-alpha; mixed sheet: order 1765234
310	d.157	7	1.000	1.000	Metallo-hydrolase/oxidoreductase	duplication of beta(4)-alpha-beta-alpha motif; 4 layers a/b/b/a; mixed beta-sheets
311	d.108	15	0.790	0.876	Acyl-CoA N-acyltransferases (Nat)	3 layers: a/b/a; contains mixed beta-sheet
312	d.109	14	0.621	0.934	Gelsolin-like	3 layers: a/b/a; contains mixed beta-sheet
313	d.117	3	1.000	1.000	Thymidylate synthase/dCMP hydroxymethylase	contains large mixed beta-sheet
314	d.168	4	0.833	1.000	Succinate dehydrogenase/fumarate reductase flavoprotein, catalytic domain	unusual fold
315	d.73	2	1.000	1.000	RuBisCO, small subunit	alpha-beta(2)-alpha-beta(2); 2 layers, alpha/beta
316	d.131	13	1.000	1.000	DNA clamp	contains two helices and two beta sheets
317	d.44	3	0.667	1.000	Fe,Mn superoxide dismutase (SOD), C-terminal domain	alpha-beta(2)-alpha-beta-alpha(2); 3 strands of antiparallel sheet: 213
318	d.118	2	0.000	1.000	N-acetylmuramoyl-L-alanine amidase-like	contains mixed beta-sheet
319	d.126	5	0.900	1.000	Pentein, beta/alpha-propeller	duplication: composed of 5 alpha-beta(2)-alpha-beta units arranged around pseudo fivefold axis
320	d.106	2	1.000	1.000	Sterol carrier protein, SCP	alpha-beta(3)-(crossover)-beta-(alpha)-beta; 3 layers: a/b/a; antiparallel beta-sheet of 5 strands; order 32145
321	d.137	3	0.333	0.833	Monooxygenase (hydroxylase) regulatory protein	corner-like structure formed by two sheets and filled in with 2-3 helices
322	d.75	3	1.000	0.667	MutS N-terminal domain-like	beta(2)-alpha-beta(2)-alpha; 2 layers, alpha/beta
323	d.77	2	1.000	1.000	Ribosomal protein L5	beta-alpha-beta(2)-alpha-beta(3)-alpha; 2 layers, alpha/beta; antiparallel beta-sheet: order 231654
324	d.175	2	0.000	1.000	Penicillin binding protein dimerisation domain	unusual fold

Table 3. True Positive Rates at 1% False Positive Rate, for VAST and SHEBA, for 468 SCOP Folds in the order of the Heat Map.

i	Class Fold	N_i	TPR VAST	TPR SHEBA	Fold Name	Fold Description
325	d.225	2	1.000	1.000	Multidrug efflux transporter AcrB TolC docking domain; DN and DC subdomains	Intertwined pseudo hexamer of an alpha+beta motif
326	d.112	3	1.000	1.000	Phoshotransferase/anion transport protein	beta-alpha(2)-beta(3)-alpha(3); 3 layers, alpha/beta/alpha; mixed sheet: order 1342; loop crossing
327	d.178	2	1.000	1.000	Aromatic aminoacid monoxygenases, catalytic and oligomerization domains	unusual fold
328	d.15	56	0.535	0.775	beta-Grasp (ubiquitin-like)	core: beta(2)-alpha-beta(2); mixed beta-sheet 2143
329	d.66	5	0.550	0.850	Alpha-L RNA-binding motif	alpha(2)-beta(2)-loop-beta; 2 layers: alpha/beta
330	d.120	4	1.000	1.000	Cytochrome b5	small, heme-binding fold
331	d.42	6	0.867	1.000	POZ domain	core: beta(2)-alpha(2)-beta(2)-alpha(2); 2 layers a/b; mixed sheet: 2143
332	d.189	3	0.333	1.000	PX domain	beta(3)-alpha(4); meander beta-sheet packed against array of helices; contains Pro-rich stretch
333	d.201	3	1.000	1.000	SRP19	beta-alpha-beta(2)-alpha; 2 layers: alpha/beta
334	d.1	5	0.400	1.000	Microbial ribonucleases	single helix packs against antiparallel beta-sheet
335	d.24	3	0.833	1.000	Pilin	contains very long N-terminal helix, which end is packed against beta-sheet
336	d.127	5	1.000	1.000	Creatinase/aminopeptidase	duplication: composed of two very similar alpha+beta folds
337	d.21	2	1.000	1.000	Diaminopimelate epimerase	mixed beta-sheet folds into a barrel (n=8, S=14) around the central helix
338	d.5	4	1.000	1.000	RNase A-like	contains long curved beta-sheet and 3 helices
339	d.93	15	0.738	1.000	SH2-like	3 layers: a/b/a; antiparallel beta-sheet of 5 strands is flaked by two helices
340	d.88	2	0.000	1.000	SRF-like	alpha-beta(2)-alpha; dimer; 3 layers a/b/a; antiparallel beta-sheet
341	d.18	2	0.000	0.000	ssDNA-binding transcriptional regulator domain	helix-swapped dimer of beta(4)-alpha motifs
342	d.85	3	0.667	1.000	RNA bacteriophage capsid protein	6-standed beta-sheet followed with 2 helices; meander
343	d.217	2	1.000	1.000	SAND domain-like	core: three short helices packed against a barrel-like beta-sheet; some similarity to the SH3-like fold
344	d.9	17	0.592	0.904	IL8-like	beta(3)-alpha
345	d.101	2	0.000	1.000	Polynucleotide phosphorylase/guanosine pentaphosphate synthase (PNPase/GPSI), domains 2 and 5	beta(2)-alpha-beta(2)-alpha; 3 layers: alpha/beta/alpha; antiparallel sheet: order 2134
346	d.19	15	0.881	0.971	MHC antigen-recognition domain	dimeric

Table 3. True Positive Rates at 1% False Positive Rate, for VAST and SHEBA, for 468 SCOP Folds in the order of the Heat Map.

i	Class Fold	N_i	TPR VAST	TPR SHEBA	Fold Name	Fold Description
347	d.83	2	0.000	1.000	Bactericidal permeability-increasing protein, BPI	beta-alpha-beta(5)-alpha-beta2; 2 layers: alpha/beta; antiparallel sheet
348	d.128	3	0.333	1.000	Glutamine synthase/guanido kinase	
349	d.179	2	1.000	1.000	Substrate-binding domain of HMG-CoA reductase	unusual fold
350	d.198	7	0.548	0.881	Secretion chaperone-like	alpha-beta(3)-alpha-beta(2)-alpha; 2 layers: alpha/beta
351	d.100	2	1.000	1.000	L9 N-domain-like	beta(2)-alpha-beta-alpha; 3 layers: alpha/beta/alpha
352	d.13	5	0.800	0.900	HIT-like	alpha-beta(3)-alpha-beta(2); 3 layers: alpha/beta/alpha
353	d.173	2	1.000	1.000	Methionine synthase activation domain-like	unusual fold; core: beta-alpha(2)-beta(2)-alpha(2)-beta-alpha-beta; antiparallel beta-sheet: order 12354
354	d.90	4	1.000	1.000	NADH oxidase/flavin reductase	core: (alpha-beta-alpha-beta)2; 3 layers a/b/a; antiparallel beta-sheet: 1243
355	d.6	2	0.000	1.000	Prion-like	beta-alpha-beta-alpha(2); antiparallel beta-ribbon
356	d.202	2	1.000	1.000	Transcription factor NusA, N-terminal domain	alpha-beta(3)-alpha-beta-alpha; bifurcated coiled beta-sheet
357	d.218	6	0.733	1.000	Nucleotidyltransferase	core: alpha-beta-turn-beta-X-beta-(alpha); mixed beta-sheet, order 123
358	d.89	4	1.000	0.917	Origin of replication-binding domain, RBD-like	alpha-beta-alpha-beta(2)-alpha-beta-alpha-beta; 3 layers:a/b/a; antiparallel sheet 41325
359	d.65	2	1.000	1.000	Hedgehog/DD-pepidase	alpha-beta(2)-alpha-beta(2); 2 layers: alpha/beta
360	d.67	5	0.500	0.250	RRF/tRNA synthetase additional domain-like	core:alpha-beta(2)-alpha-beta(2); 2 layers: alpha/beta
361	d.181	2	1.000	1.000	Insert subdomain of RNA polymerase alpha subunit	unusual fold; contains a left-handed beta-alpha-beta unit
362	d.212	3	0.333	0.333	TolA/TonB C-terminal domain	beta(2)-alpha-beta; 2 layers, alpha/beta; left-handed beta-alpha-beta unit in unswapped monomer
363	d.31	4	0.333	1.000	Cdc48 domain 2-like	beta-alpha-beta(3); 2 layers: alpha/beta
364	d.43	3	1.000	0.667	Elongation factor Ts (EF-Ts), dimerisation domain	beta(2)-alpha(n)-beta; 2 layers a/b; antiparallel sheet: 123
365	d.96	7	1.000	1.000	T-fold	beta(2)-alpha(2)-beta(2); 2 layers: alpha/beta; antiparallel sheet 1234
366	d.59	2	1.000	1.000	Ribosomal protein L30p/L7e	core: beta-alpha-beta-alpha-beta; antiparallel beta-sheet: order 312; some similarity with the ferredoxin-like fold
367	d.10	5	0.600	0.450	DNA-binding domain	beta(3)-alpha; 2 layers: alpha/beta
368	d.50	9	0.611	0.778	dsRBD-like	alpha-beta(3)-alpha; 2 layers: alpha/beta
369	d.134	2	1.000	1.000	Sulfite reductase hemoprotein (SiRHP), domains 2 and 4	beta-alpha-beta-alpha-beta(3)-alpha(2,3); mixed sheet: order 12345; left-handed crossover connection bettween strands 2 and 3
370	d.20	8	0.857	1.000	UBC-like	alpha-beta(4)-alpha(3); core: meander beta-sheet plus one helix 2
371	d.82	3	0.667	0.333	N domain of copper amine oxidase-like	alpha-beta(5)-alpha; 2 layers: alpha/beta; meander antiparallel sheet

Table 3. True Positive Rates at 1% False Positive Rate, for VAST and SHEBA, for 468 SCOP Folds in the order of the Heat Map.

i	Class Fold	N_i	TPR VAST	TPR SHEBA	Fold Name	Fold Description
372	d.111	2	1.000	1.000	PR-1-like	alpha-beta-alpha-beta-alpha(2)-beta(2); 3 layers, alpha/beta/alpha; mixed sheet: order 1342
373	d.208	2	1.000	1.000	MTH1598-like	beta(2)-alpha-beta-alpha-beta(4); 3 layers: beta/alpha/beta; some similarity to the Hsp33 fold
374	d.60	3	1.000	1.000	Probable bacterial effector-binding domain	duplication of beta-alpha-beta(2) motif: antiparallel beta sheet forms barrel (n=6, S=12)
375	d.140	2	1.000	1.000	Ribosomal protein S8	consists of 2 different alpha+beta subdomains arranged in a 4-layer structure: b/a/b/a
376	d.68	10	0.422	0.611	IF3-like	beta-alpha-beta-alpha-beta(2); 2 layers; mixed sheet 1243, strand 4 is antiparallel to the rest
377	d.150	3	1.000	1.000	4'-phosphopantetheinyl transferase	beta-alpha(3)-beta(2) motif
378	d.22	3	1.000	1.000	GFP-like	beta-sheet folds into a barrel (n=11, S=14) around the central helix
379	d.38	5	0.600	1.000	Thioesterase/thiol ester dehydrase-isomerase	core: beta-alpha-beta(4); 2 layers: alpha/beta
380	d.129	14	0.560	0.588	TBP-like	beta-alpha-beta(4)-alpha
381	d.16	14	0.418	0.753	FAD-linked reductases, C-terminal domain	alpha+beta sandwich
382	d.41	10	0.711	0.800	alpha/beta-Hammerhead	core: beta-BETA-alpha-beta-BETA-beta-alpha; contains a beta-hammerhead motif similar to that in barrel-sandwich hybrids
383	d.146	2	1.000	1.000	Uridine diphospho-N-Acetylenolpyruvylglucosamine reductase, MurB, C-terminal domain	consists of two alpha+beta subdomains
384	d.86	2	1.000	1.000	Translation initiation factor eIF4e	beta(2)-alpha-beta(2)-alpha-beta(2)-alpha-beta-2; 2 layers: alpha/beta; antiparallel sheet: 21356478
385	d.151	4	1.000	1.000	DNase I-like	contains beta-sandwich; duplication of alpha+beta motif
386	d.160	3	1.000	1.000	Nitrilase/N-carbamoyl-D-aminoacid amidohydrolase	4 layers: alpha/beta/beta/alpha; mixed beta sheets; contains duplication
387	d.159	8	0.643	0.982	Metallo-dependent phosphatases	4 layers: alpha/beta/beta/alpha; mixed beta sheets; contains duplication
388	d.12	2	0.000	0.500	Ribosomal proteins L23 and L15e	beta-(alpha)-beta-alpha-beta(2); 3 layers: alpha/beta/alpha; antiparallel beta-sheet: order 1243
389	d.95	8	0.679	0.946	Homing endonuclease-like	alpha-beta(2)-alpha-beta(2)-alpha; 2 layers: a/b; antiparallel sheet 1243
390	d.206	2	1.000	1.000	YggU-like	beta(2)-loop-alpha-beta(2)-alpha; 2 layers: a/b; antiparallel sheet 1243; some similarity to the Homing endonuclease-like fold
391	d.64	2	1.000	1.000	eIF1-like	beta(2)-alpha-beta(2)-alpha-beta; 2 layers: alpha/beta; mixed beta-sheet: order 51243

Table 3. True Positive Rates at 1% False Positive Rate, for VAST and SHEBA, for 468 SCOP Folds in the order of the Heat Map.

i	Class Fold	N_i	TPR VAST	TPR SHEBA	Fold Name	Fold Description
392	d.54	8	0.964	1.000	Enolase N-terminal domain-like	beta(3)-alpha(3); meander and up-and-down bundle
393	d.55	2	1.000	1.000	Ribosomal protein L22	beta-alpha(3)-beta(2); 2 layers: alpha/beta; related to the enolase/MLE N-domain fold by a circular permutation
394	d.11	2	1.000	1.000	Penicillin-binding protein 2x (pbp-2x), c-terminal domain	alpha1-beta3; 2 layers: alpha/beta; order 132
395	d.40	3	1.000	1.000	CI-2 family of serine protease inhibitors	alpha+beta sandwich; loop across free side of beta-sheet
396	d.37	3	0.667	1.000	CBS-domain	core: beta-alpha-beta(4); 2 layers: alpha/beta
397	d.130	3	0.667	1.000	S-adenosylmethionine synthetase	duplication: consists of 3 similar intertwined domains
398	d.80	6	0.600	0.967	Tautomerase/MIF	(beta-alpha-beta)2; 2 layers: alpha/beta; mixed beta-sheet
399	d.52	10	0.622	0.833	Alpha-lytic protease prodomain-like	core: alpha-beta(2)-(alpha)-beta; 2 layers: alpha/beta
400	d.49	2	1.000	1.000	Signal recognition particle alu RNA binding heterodimer, SRP9/14	(beta)-alpha-beta(3)-alpha; 2 layers, alpha/beta
401	d.56	2	1.000	1.000	GroEL-intermediate domain like	3-helical bundle packed against 3-stranded mixed beta-sheet
402	d.105	2	1.000	1.000	Clathrin adaptor appendage domain	beta-alpha-beta-alpha-beta(4)-alpha; 3 layers: a/b/a; bifurcated antiparallel beta-sheet
403	d.141	4	1.000	1.000	Ribosomal protein L6	consists of two beta-sheets and one alpha-helix packed around single core
404	d.94	4	1.000	1.000	HPr-like	beta-alpha-beta(2)-alpha-beta-alpha; 2 layers: a/b; antiparallel sheet 1423
405	d.51	7	1.000	1.000	Eukaryotic type KH-domain (eKH-domain)	beta-alpha(2)-beta(2)-alpha; 2 layers: alpha/beta
406	d.58	133	0.530	0.512	Ferredoxin-like	alpha+beta sandwich with antiparallel beta-sheet; (beta-alpha-beta)x2
407	d.74	8	0.679	0.750	DCoH-like	beta(2)-alpha-beta(2)-alpha; 2 layers, alpha/beta
408	d.45	2	1.000	1.000	ClpS-like	beta-alpha(2)-beta-alpha-beta; 2 layers, alpha/beta
409	e.26	3	0.333	0.333	Prismane protein-like	3 domains: (1) spectrin repeat-like 3-helical bundle; (2 and 3) alpha/beta: Rossmann-fold topology
410	e.8	14	0.077	0.297	DNA/RNA polymerases	(
411	e.23	3	1.000	1.000	Firefly luciferase-like	4 domains: (1&2) duplication: share the same alpha/beta fold; (3) beta-barrel; (4) alpha+beta
412	e.5	3	1.000	1.000	Heme-linked catalases	contains an (8,10) beta-barrel and an all-alpha domain
413	e.11	2	1.000	1.000	Type II DNA topoisomerase	4 domains: (1) toprim alpha/beta; (2) "winged helix"-like; (3) alpha+beta; (4) all-alpha

Table 3. True Positive Rates at 1% False Positive Rate, for VAST and SHEBA, for 468 SCOP Folds in the order of the Heat Map.

i	Class Fold	N_i	TPR VAST	TPR SHEBA	Fold Name	Fold Description
414	e.25	2	1.000	1.000	Sec1/munc18-like (SM) proteins	3 domains: (1 and 2) alpha+beta; (3) mostly alpha, inserted in domain 2
415	e.3	14	1.000	1.000	beta-Lactamase/D-ala carboxypeptidase	contains a cluster of helices and an alpha+beta sandwich
416	e.10	3	1.000	1.000	Prokaryotic type I DNA topoisomerase	4 domains: (1) Toprim alpha/beta; (2&4) "winged helix"-like; (3) barrel: n=6, S=8
417	e.1	6	1.000	1.000	Serpins	contains a cluster of helices and a beta-sandwich
418	e.6	5	1.000	1.000	Acyl-CoA dehydrogenase NM domain-like	2 domains: (1) all-alpha: 5 helices; (2) contains an open beta-sheet barrel: n*=5, S*=8; complex topology
419	e.18	2	1.000	1.000	Nickel-iron hydrogenase, large subunit	3 domains: (1) all-alpha; (2&3) alpha+beta
420	e.4	2	1.000	1.000	ADP ribosyl cyclase-like	contains a cluster of helices and an alpha/beta domain
421	e.24	3	0.667	1.000	Ribosomal protein L1	2 domains: (1) alpha+beta; (2) alpha/beta (interrupts domain 1)
422	e.17	4	1.000	1.000	D-aminoacid aminotransferase-like PLP-dependent enzymes	2 domains: (1) alpha+beta: beta3-alpha2-beta2; (2) alpha/beta, a part of its mixed sheet forms barrel: n=6, S=8
423	e.39	2	1.000	1.000	YebC-like	3 domains: (1) 3-helical bundle; (2 and 3) alpha+beta of different folds: domain 3 has a ferredoxin-like fold and is inserted in domain 2
424	e.7	7	1.000	1.000	Sugar phosphatases	N-terminal domain is an alpha+beta, C-terminal domain is an alpha/beta with mixed beta-sheet
425	e.31	2	1.000	1.000	Ku heterodimer subunits	3 domains: (1) integrin A-like alpha/beta; (2) beta-barrel (n=7, s=10); (3) helical arm with an HTH motif
426	e.22	3	1.000	1.000	Dehydroquinate synthase-like	2 domains: (1) alpha/beta of a Rossmann-fold topology, binds NAD (2) multihelical array
427	f.4	14	0.192	0.654	Transmembrane beta-barrels	not a true fold, gathers together transmembrane barrels of different (n,S)
428	f.7	3	0.000	0.167	Lipovitellin-phosvitin complex; beta-sheet shell regions	
429	f.1	11	0.145	0.182	Toxins' membrane translocation domains	multi-helical domains of various folds which is thought to unfold in the membrane
430	f.23	5	0.000	0.500	Single transmembrane helix	not a true fold
431	f.21	7	0.143	0.619	Heme-binding four-helical bundle	core: four transmembrane helices, up-and-down bundle, binds one or two heme groups in between the helices
432	f.26	2	1.000	1.000	Bacterial photosystem II reaction centre, L and M subunits	five transmembrane helices forming a sheet-like structure
433	f.19	2	1.000	1.000	Aquaporin-like	core: 8 helices, 2 short helices are surrounded by 6 long transmembrane helices
434	f.10	2	0.000	1.000	Viral glycoprotein, central and dimerisation domains	2 intertwined domains; all-beta and alpha+beta

Table 3. True Positive Rates at 1% False Positive Rate, for VAST and SHEBA, for 468 SCOP Folds in the order of the Heat Map.

i	Class Fold	N_i	TPR VAST	TPR SHEBA	Fold Name	Fold Description
435	f.6	2	1.000	1.000	Leukocidin (pore-forming toxin)	subunit fold contains beta-sandwich of Ig-like (grek-key) topology
436	f.35	2	1.000	1.000	Multidrug efflux transporter AcrB transmembrane domain	12 transmembrane helices; duplication: the N- and C-terminal halves of the whole proteins are structurally similar
437	f.17	2	0.000	1.000	Transmembrane helix hairpin	two antiparallel transmembrane helices
438	f.27	2	1.000	1.000	14 kDa protein of cytochrome bc1 complex (Ubiquinol-cytochrome c reductase)	membrane-associated alpha-helical protein; no transmembrane helices
439	f.13	4	0.500	1.000	Family A G protein-coupled receptor-like	core: up-and-down bundle of seven transmembrane helices tilted 20 degrees with respect to the plane of the membrane
440	f.24	3	1.000	1.000	Cytochrome c oxidase subunit I-like	12 transmembrane helices in an approximate threefold rotational symmetric arrangement
441	f.25	2	1.000	1.000	Cytochrome c oxidase subunit III-like	core: 7 transmembrane helices organised into two bundles, one formed by the first two helices and the other by the rest
442	f.14	2	0.000	1.000	Voltage-gated potassium channels	oligomeric transmembrane alpha-helical proteins
443	f.28	2	1.000	1.000	Nonheme 11 kDa protein of cytochrome bc1 complex (Ubiquinol-cytochrome c reductase)	membrane-associated alpha-helical protein; no transmembrane helices
444	g.39	12	0.152	0.636	Glucocorticoid receptor-like (DNA-binding domain)	alpha+beta metal(zinc)-bound fold
445	g.50	3	0.000	1.000	FYVE/PHD zinc finger	dimetal(zinc)-bound alpha+beta fold
446	g.49	3	0.000	1.000	Cysteine-rich domain	dimetal(zinc)-bound alpha+beta fold
447	g.44	7	0.429	1.000	RING finger domain, C3HC4	dimetal(zinc)-bound alpha+beta motif; structurally diverse
448	g.37	5	0.400	1.000	C2H2 and C2HC zinc fingers	alpha+beta metal(zinc)-bound fold: beta-hairpin + alpha-helix
449	g.10	3	1.000	1.000	Hairpin loop containing domain-like	alpha+beta fold with two crossing loops
450	g.7	10	0.722	1.000	Snake toxin-like	disulphide-rich fold: nearly all-beta
451	g.8	9	0.944	1.000	BPTI-like	disulphide-rich alpha+beta fold
452	g.14	5	0.100	1.000	Kringle-like	disulphide-rich fold; nearly all-beta
453	g.15	10	0.633	0.767	Ovomucoid/PCI-1 like inhibitors	disulphide-rich small alpha+beta fold
454	g.41	21	0.117	0.350	Rubredoxin-like	metal(zinc or iron)-bound fold; sequence contains two CX(n)C motifs, in most cases n = 2
455	g.38	2	0.000	1.000	Zn2/Cys6 DNA-binding domain	all-alpha dimetal(zinc)-bound fold
456	g.17	12	0.129	0.902	Cystine-knot cytokines	disulphide-rich fold; common core is all-beta
457	g.35	4	0.500	1.000	HIPIP (high potential iron protein)	folds around 4Fe-4S cluster

Table 3. True Positive Rates at 1% False Positive Rate, for VAST and SHEBA, for 468 SCOP Folds in the order of the Heat Map.

i	Class Fold	N_i	TPR VAST	TPR SHEBA	Fold Name	Fold Description
458	g.51	2	1.000	1.000	Zn-binding domains of ADDBP	metal(zinc)-bound alpha+beta fold
459	g.52	4	1.000	1.000	Inhibitor of apoptosis (IAP) repeat	metal(zinc)-bound alpha+beta fold
460	g.53	2	0.000	1.000	TAZ domain	all-alpha fold; Zn-binding sites are in the loops connecting helices
461	g.22	3	0.000	1.000	Serine proterase inhibitors	disulphide-rich; nearly all-beta
462	g.24	5	0.000	0.900	TNF receptor-like	duplication: consists of three similar disulphide-rich domains
463	g.9	9	0.361	0.889	Defensin-like	Disulphide-rich fold, nearly all-beta
464	g.3	38	0.238	0.472	Knottins (small inhibitors, toxins, lectins)	disulphide-bound fold; contains beta-hairpin with two adjacent disulphides
465	g.18	16	0.563	0.992	Complement control module/SCR domain	disulphide-rich all-beta fold
466	g.27	5	0.900	1.000	Fibronectin type I module	disulphide-rich, all-beta
467	g.4	2	1.000	1.000	PMP inhibitors	disulphide-rich fold; all-beta: 3 antiparallel strands
468	g.59	2	0.000	0.500	Zinc-binding domain of translation initiation factor 2 beta	zinc-bound beta-ribbon motif
